# Supplementary material for: Societal and organisational influences on implementation of mental health peer support work in low-income and high-income settings: a qualitative focus group study
Source: BMJ Open. 2023 Aug 23;13(8):e058724. doi: 10.1136/bmjopen-2021-058724 (PMC10450133; doi:10.1136/bmjopen-2021-058724)
Supplement: Supplementary data [file bmjopen-2021-058724supp001.pdf]

UPSIDES

Instructions:

This guide is to be used to facilitate focus group discussion with (1.1) health workers who are using or plan to recruit peer support workers. (Psychiatric nurses, clinicians working in mental health ward / departments) and (1.2) local stakeholders with relevant expertise relating to implementation of peer work (including clinicians and managers who currently, previously or in the future may employ peer support workers, and people who currently, previously or in the future may work as peer support workers).

Interviewer ID ___ ___ ___ ___

Interview date (DD/MM/YYYY): __ __ / __ __ / __ __ __ __

Location of the interview ____________________________________

Country: ____________________________________

Introduction:

Ensure that the participants are comfortable.

Hi and welcome to this session. My name is ___ and my colleague is___. Thank you for taking your time to talk to us about your experiences with people with severe mental illness and peer support workers. We will discuss topics related to the key characteristics of peer support workers and the challenges they may face. The discussion will take around 60minutes. The interview will be recorded using an audio recorder as we will not have to write down all your answers and we will not miss any of your useful comments. Informed consent will be obtained from all the participants and confidentiality will be assured to all the participants. Before we begin, I would like to know if you have any questions.

Explain you are starting the audio recorder.

Time interview started:

Theme 1: Socio-demographic information of the respondents

Collect data on name, age, education, position and number of years working in this position and give a code to each participant.

Explain again to the participant that:

- I want to learn about your experience, thoughts and perspective on this topic of using peer support workers for improving mental health conditions in your country.
- There is no 'right' or 'wrong' answer.

Say: We are planning to recruit and train peer support workers. Peer providers in mental health are individuals with severe mental illness who are further along in their recovery, who support others with similar conditions by role modelling that recovery is possible, sharing knowledge from experience and using reciprocal empathic relationships. We are thinking of asking these peer support workers to identify and visit individuals with severe mental illness. We would like to get your opinion on how best to implement PSWs intervention in this region. We would like to know key characteristics for PSWs and challenges that could hinder them performing their

Ramesh M, et al. BMJ Open 2023; 13:e058724. doi: 10.1136/bmjopen-2021-058724

tasks. Please be frank as we want the information to be the right ones for this community.

Theme 2: Social cultural factors and acceptability of PSWs

1. Based on your experiences, how does the community care for people with SMIs? Where do they seek care? Are there other places apart from the hospital? Describe all the places people seek care for SMI.

2. What are your main roles in supporting people with SMIs? What are the main challenges that you are facing in doing your job? What are the solutions to those challenges?

   - Probe to know if mental health programmes are given a priority?
   - Is there a national policy or guideline for mental health conditions? Are these accessible?
   - What has been the main challenge your department has been facing in managing/meeting the needs of people with Severe Mental Illness? Probe for various challenges the department has been facing in regard to SMI programmes? For each challenge mentioned, ask how the department could handle it i.e. any potential solution and/or suggestion on how to address it?

3. With your experience working in this area, could you please tell us what do you know about PSWs and how do they perform their duties?

   - Probe for, how they are recruited, who recruits them, what are they exactly doing, etc?
   - Please, describe any systems for linking people with peers who can serve as role models in this facility or community? (e.g. through contact with local user-run groups).

4. What key qualities would you want a peer support worker to have or NOT to have? We want to find out what sort of person the respondents would trust and value, perform the work well and responsibly.

5. Could you please tell us what are the specific things, you would want a peer support worker to do?

   Probe for specific terminologogies, main activities, duration of activities. care planning

Theme 3: Institution / facility readiness to incorporate PSW

6. In your opinion – for using peer support workers, how could it be implemented / improved at this facility?

Ramesh M, *et al. BMJ Open* 2023; 13:e058724. doi: 10.1136/bmjopen-2021-058724

UPSIDES

- Probe: training, logistics, management, supervision, monitoring, capacity development, resources etc.

7. What are the facilitators and barriers in providing peer support to people with SMI in your facility or in your region?

    - Probe for resources, skilled personnel, infrastructure, systems support?

8. Is there anything more you would like to add about your experiences with, or views on, using peer support workers?

Time interview end:

Thank the participant for his / her time. Remind them that the information will be kept confidential.

Interviewer comments on how the interview went:
