# Supplementary material for: Societal and organisational influences on implementation of mental health peer support work in low-income and high-income settings: a qualitative focus group study
Source: BMJ Open. 2023 Aug 23;13(8):e058724. doi: 10.1136/bmjopen-2021-058724 (PMC10450133; doi:10.1136/bmjopen-2021-058724)
Supplement: Author's manuscript [file bmjopen-2021-058724.draft_revisions.pdf]

BMJ Open

BMJ Open is committed to open peer review. As part of this commitment we make the peer review history of every article we publish publicly available.

When an article is published we post the peer reviewers' comments and the authors' responses online. We also post the versions of the paper that were used during peer review. These are the versions that the peer review comments apply to.

The versions of the paper that follow are the versions that were submitted during the peer review process. They are not the versions of record or the final published versions. They should not be cited or distributed as the published version of this manuscript.

BMJ Open is an open access journal and the full, final, typeset and author-corrected version of record of the manuscript is available on our site with no access controls, subscription charges or pay-per-view fees (http://bmjopen.bmj.com).

If you have any questions on BMJ Open's open peer review process please email info.bmjopen@bmj.com

BMJ Open

Societal and organisational influences on implementation of mental health peer support work in low-income and high-income settings: qualitative focus group study

Journal:	*BMJ Open*
Manuscript ID	bmjopen-2021-058724
Article Type:	Original research
Date Submitted by the Author:	26-Oct-2021

Complete List of Authors:	Ramesh, Mary; Ifakara Health Institute, Department of Health System, Impact Evaluation and Policy<br>Charles, Ashleigh; University of Nottingham, School of Health Sciences<br>Grayzman, Alina; Ben-Gurion University of the Negev Faculty of Health Sciences, Department of Social Work, Ben Gurion University of the Negev<br>Hiltensperger, Ramona; Department of Psychiatry II<br>Kalha, Jasmine; Indian Law Society<br>Kulkarni, Arti; Indian Law Society<br>Mahlke, Candelaria ; University Medical Cente Hamburg, Candelaria Irene Mahlke<br>Moran, Galia; Ben-Gurion University of the Negev, Department of Social Work<br>Mpango, Richard; Butabika National Referral Hospital; Soroti University<br>Stierlin, Annabel; Ulm University, Institute of Epidemiology and Medical Biometry<br>Nixdorf, Rebecca ; University Medical Centre Hamburg-Eppendorf, Department of Psychiatry<br>Ryan, Grace; London School of Hygiene and Tropical Medicine, Population Health<br>Shamba, Donat ; Ifakara Health Institute, Department of Health Systems, Impact Evaluation and Policy<br>Slade, Mike; University of Nottingham School of Health Sciences

Keywords:	MENTAL HEALTH, Adult psychiatry < PSYCHIATRY, QUALITATIVE RESEARCH, PUBLIC HEALTH

SCHOLARONE™
Manuscripts

I, the Submitting Author has the right to grant and does grant on behalf of all authors of the Work (as defined in the below author licence), an exclusive licence and/or a non-exclusive licence for contributions from authors who are: i) UK Crown employees; ii) where BMJ has agreed a CC-BY licence shall apply, and/or iii) in accordance with the terms applicable for US Federal Government officers or employees acting as part of their official duties; on a worldwide, perpetual, irrevocable, royalty-free basis to BMJ Publishing Group Ltd ("BMJ") its licensees and where the relevant Journal is co-owned by BMJ to the co-owners of the Journal, to publish the Work in this journal and any other BMJ products and to exploit all rights, as set out in our licence.

The Submitting Author accepts and understands that any supply made under these terms is made by BMJ to the Submitting Author unless you are acting as an employee on behalf of your employer or a postgraduate student of an affiliated institution which is paying any applicable article publishing charge ("APC") for Open Access articles. Where the Submitting Author wishes to make the Work available on an Open Access basis (and intends to pay the relevant APC), the terms of reuse of such Open Access shall be governed by a Creative Commons licence – details of these licences and which Creative Commons licence will apply to this Work are set out in our licence referred to above.

Other than as permitted in any relevant BMJ Author's Self Archiving Policies, I confirm this Work has not been accepted for publication elsewhere, is not being considered for publication elsewhere and does not duplicate material already published. I confirm all authors consent to publication of this Work and authorise the granting of this licence.

For peer review only

Societal and organisational influences on implementation of mental health peer support work in low-income and high-income settings: qualitative focus group study

Mary Ramesh [1 a]
Ashleigh Charles [2 a]
Alina Grayzman [3]
Ramona Hiltensperger [4]
Jasmine Kalha [5]
Arti Kulkarni[5]
Candelaria Mahlke [6]
Galia Moran[3]
Richard Mpango [7 8 9]
Annabel Sandra Müller-Stierlin [4]
Rebecca Nixdorf [6]
Grace Ryan [10]
Donat Shamba [1 b *]
Mike Slade [2 b]

[1] Department of Health Systems, Impact Evaluation and Policy, Ifakara Health Institute, Dar es Salaam, Tanzania
[2] School of Health Sciences, Institute of Mental Health, University of Nottingham, UK
[3] Department of Social Work, Ben Gurion University of the Negev, Be'er Sheva, Israel
[4] Department of Psychiatry II, Ulm University, Ulm, Germany
[5] Centre for Mental Health Law and Policy, Indian Law Society, Pune, India
[6] Department of Psychiatry, University Medical Center Hamburg-Eppendorf, Germany
[7] Butabika National Referral Hospital, Uganda
[8] School of Health Sciences, Soroti University, Soroti, Uganda
[9] MRC/UVRI and LSHTM Uganda Research Unit, Entebbe, Uganda
[10] Centre for Global Mental Health, London School of Hygiene and Tropical Medicine, UK

[a] Ramesh and Charles contributed equally and are joint first authors
[b] Shamba and Slade contributed equally and are joint last authors

* Correspondence to: Donat Shamba: dshamba@ihi.or.tz

ORCID iDs
MR: 0000-0002-4942-3923; AC: 0000-0003-2222-4358; AG 0000-0002-8192-7697; RH: 0000-0003-2544-4188; JK: 0000-0001-7357-2366; AK: 0000-0002-3281-4388; CM: 0000-0001-9573-6106;GM: 0000-0001-9718-1773; RM: 0000-0001-6960-3174; AMS: 0000-0003-2812-5115; RN: 0000-0002-3064-8380; GR: 0000-0002-9310-3513; DS: 0000-0001-7431-7199; MS: 0000-0001-7020-3434

Word count
3,988 words.

For peer review only
For peer review only - http://bmjopen.bmj.com/site/about/guidelines.xhtml

ABSTRACT

Objectives
Despite the established evidence base for mental health peer support work, widespread implementation remains a challenge. This study aimed to explore societal and organisational influences on the implementation of peer support work in low and high income settings.

Design
Study sites conducted two focus groups in local languages at each site, using a topic guide based on a conceptual framework describing eight peer support worker principles and five implementation issues. Transcripts were translated into English and an inductive thematic analysis was conducted to characterise implementation influences.

Setting
The study took place in five secondary mental health care sites as part of the Using Peer Support in Developing Empowering Mental Health Services (UPSIDES) study, comprising three high-income sites (Hamburg and Ulm, Germany; Be'er Sheva, Israel) and two low-income sites (Dar es Salaam, Tanzania; Kampala, Uganda) chosen for diversity both in region and in experience of peer support work.

Participants
84 participants (56 female, 30 male) met inclusion criteria: aged over 18 years; actual or potential peer support worker (n=26) or mental health clinician (n=10) or hospital / community manager or regional / national policy-maker (n=10); able to give informed consent.

Results
Seven themes relating to implementation influences were identified: community attitudes; resource availability; organisational culture; staff attitudes; role definition; training and support; and peer support network.

Conclusions
This is the first multi-country study to explore societal attitudes and organisational culture influences on the implementation of peer support. Addressing community-level discrimination and developing a recovery orientation in mental health systems can contribute to effective implementation of peer support work. The UPSIDES randomised controlled trial will investigate implementation of peer support work in different resource setting. The relationship between societal stigma about mental health and resource allocation decisions warrants future investigation.

Trial registration
UPSIDES RCT: ISRCTN26008944

For peer review only

For peer review only - http://bmjopen.bmj.com/site/about/guidelines.xhtml

ARTICLE SUMMARY

Strengths and limitations of this study

- The sample size (n=84) and sampling across two low-income and three high-income sites increases the credibility of the findings and their relevance for both low- and high-income settings.
- All focus groups were held in the local language with translation of topics guides focused on conceptual equivalence, to increase accessibility to people without high proficiency in English.
- Independent coding by multiple analysts from different cultures enhances trustworthiness.
- Sociodemographic characteristics were not sufficiently collected to be reported, limiting transferability of findings.
- Credibility could be further enhanced by involving people with lived experience as co-analysts in collaborative data analysis, and by member checking of the coding framework.

For peer review only

For peer review only - http://bmjopen.bmj.com/site/about/guidelines.xhtml

Introduction

Many people living with severe mental illness do not receive adequate care. For example, in Europe the EuroPoPP-MH study found that a comprehensive range of community-based services existed in only eight of 29 countries.[1] The resulting gap between demand and supply is called the treatment gap, or care gap.[2] While mental health has been identified as a global priority,[3] the mental health treatment gap remains, and is largest in low-resource settings.[4]

One reason for the treatment gap in low-resource settings is that global mental health initiatives do not sufficiently address contextual aspects, such as up-stream social determinants, geographic and linguistic differences, and sociodemographic influences such as ethnicity, caste and tribe.[5] This leads to barriers in receiving mental health treatment, including stigma, social exclusion and differing availability of resources.

Mental health peer support is an established intervention involving a person with lived experience of mental health problems and recovery employed to offer support to others with mental health problems. Peer support workers (PSWs) act as credible role models of recovery,[6] instilling hope through positive self-disclosure, modelling the use of experiential knowledge for self-care, and offering supportive relationships based on intentional mutuality.[7] There is a strong empirical evidence base for PSWs.[8-11] The most recent systematic review identified 19 randomised controlled trials,[12] all from high income countries. This review found PSW was associated with beneficial outcomes in relation to supporting recovery, empowerment and social networks. However, heterogeneity in the implementation of peer support was identified as an important knowledge gap.

Most research on mental health peer support work has been conducted in high-resource settings, including creation of core PSW principles[13,14] and evaluation.[8] However, PSW roles are increasingly being developed, formalised, and implemented in more diverse settings, such as China,[15] India,[16] Israel,[17] Singapore[18] and Uganda.[19] An important knowledge gap therefore exists in relation to PSW implementation influences across settings with different resource levels.[20] A recent systematic review synthesised 53 studies to identify 14 influences on implementation of mental health PSW.[21] The most commonly influence was organisational culture, identified in 53% of studies. training, and role definition. Societal influences were also identified, including PSW access to a peer network, resource availability and financial arrangements.

The Grand Challenges for Mental Health initiative identified the importance of research along the translation continuum including implementation, and emphasised that implementation is a challenge not just in low and middle income countries.[3] In other words, the focus should be on implementation research including both lower and higher resource settings. To our knowledge, no study has explored PSW implementation across multiple countries. The aim of this study was to explore and characterise the societal and organisational influences on the implementation of mental health peer support work in lower and higher income settings.

Methods

This study was conducted as part of UPSIDES (Using Peer Support in Developing Empowering Mental Health Services), a 5-year (2018-2022) European Union–funded multinational study that aims to replicate and scale up peer support interventions for people with severe mental illness.[22] UPSIDES involves a randomised controlled trial (ISRCTN26008944)[23] evaluating the implementation of a PSW intervention.[24]

Design

For peer review only - http://bmjopen.bmj.com/site/about/guidelines.xhtml

A qualitative research design informed by a critical realist perspective was used. A critical realist approach was chosen as it can help in identifying some of the underlying organisational and societal influences of PSW implementation. Focus groups were chosen over other data collection approaches to maximise breadth of data coverage.

Setting

Data were collected from five UPSIDES study sites. Sites were based in two high-income countries (Hamburg and Ulm sites, Germany; Be'er Sheva, Israel) and two low-income countries (Dar es Salaam, Tanzania [low resource setting at the time of data collection, re-banded in 2020 to lower-middle resource setting]; Kampala, Uganda), ensuring regional diversity (Europe, Eastern Mediterranean, sub-Saharan Africa). Sites were classified as low-resource settings because they are based in low-resource countries. As previously reported,[21] sites were also diverse in terms of their experience with peer support work, with two sites (Dar es Salaam, Ulm) having no or very little previous experience.

Participants

Participants were purposively selected to include stakeholders with different perspectives on PSW implementation: actual or potential PSWs, mental health clinicians or managers from hospitals or community services; and regional or national policy-makers. To be included, participants had to be over 18 years of age and capable of providing informed consent.

Participants came from a range of community-based, outpatient and inpatient mental health services (Ulm, Germany), University Medical Centre Hamburg-Eppendorf (Hamburg, Germany), Butabika Hospital (Uganda), the Psychiatry and Mental Health Department at Muhimbili National Hospital (Tanzania), and from a range of community mental health rehabilitation services in Israel. In all sites, multidisciplinary in-patient and out-patient care involves psychotherapy, psychosocial rehabilitation and psychiatric clinics, with some sites also offering family intervention, vocational skills training, cognitive enhancement therapy, psychoeducation, pre-discharge social interventions and physical health care.

Procedures

A conceptual framework – a network of interlinked concepts together providing a comprehensive understanding of a phenomenon[25] – was developed to capture the key elements and implementation influences on the PSW role. The conceptual framework comprised (a) PSW principles and (b) societal and organisational implementation influences. The PSW principles were derived from a researcher-led integration of established core principles from high-resource settings.[13,14,26] At the time of development (2017) there was an absence of integrated evidence, so a systematic review was subsequently undertaken[21], but for the current study the implementation influences were developed through consultation with experts in the UPSIDES consortium.

The conceptual framework informed the development of a topic guide (Supplement 1), comprising open-ended conversational prompts to explore the cultural applicability of PSW principles and to identify societal and organisational implementation influences. Exploration of areas of disagreement was encouraged, as was speculation about potential implementation influences in sites with no experience of PSW. The topic guide was developed in English, commented on by all sites, and then finalised and translated into Kiswahili (Dar es Salaam), German (Hamburg/Ulm), Hebrew (Be'er Sheva) and Luganda (Kampala).

Focus groups were conducted at each site between September and December 2018. In each site, potential participants were identified by mental health clinicians and UPSIDES research

For peer review only - http://bmjopen.bmj.com/site/about/guidelines.xhtml

workers. Two mixed focus groups were conducted per site, apart from Kampala where four focus groups (two for PSWs, two for other stakeholders) with a total of 29 participants and three individual interviews were conducted. All focus groups were conducted in the local language and held in a health service or community venue.

Each focus group comprised five to nine participants, and lasted up to 60 minutes. Facilitators were UPSIDES research workers from the site, who were bilingual in the local language and English, and came from psychology, sociology, health sciences, social work and nursing backgrounds. All facilitators were experienced in qualitative data collection, and actively managed group dynamics to ensure full participation from all participants. Focus groups were recorded using an audio recorder and researchers took field notes during the discussions. After the focus groups, local language transcripts were made, with pseudonymisation of identifying information about participants and third parties. Each local language transcript was translated into English by the local UPSIDES researcher, and checked by the UPSIDES translation leads (Nottingham, UK and Pune, India) for data integrity, identifying points for site checking if needed. Finalised transcripts were password protected and uploaded to a restricted area on the UPSIDES website.

Sampling

To manage complexity across this multi-national study, a pre-defined sampling strategy of two focus groups per site rather than sampling saturation was used.

Ethics approval

Ethical approval was obtained by each site: Ulm University Ethics Commission (Application nr. 195/18), Mengo IRB Uganda (MH: 360; MH/REC/141/8/2018), National Institute for Medical Research Tanzania (NIMR/HQ/R.8a/Vol.IX/2982), Institutional Review Board, Ifakara Health Institute, Tanzania (IHI/IRB/No. 28 – 2018), Ärztekammer Hamburg, Germany (MC-230/18), Indian Council of Medical Research (Indo-foreign/66/M/2017-NCD-1), Indian Law Society (ILS/37/2018), and Human Subjects Research Committee of Ben-Gurion University (ref: 1621-2). Each potential participant was given an information sheet in the local language, and the opportunity to ask questions. All participants gave written informed consent before participation.

Patient and public involvement

Individuals with lived experience are involved at multiple levels of the UPSIDES Study, including as part of the site team, as advisory board members, as peer support workers and as authors on some papers.

Analysis

A combination of deductive and Inductive thematic analysis was conducted.[27] The two primary coders were UPSIDES research workers in Dar es Salaam (MR: background in public health) and Nottingham (AC: mental health nursing, sociology). MR and AC independently read all transcripts to familiarise themselves with the content, and start the process of creating preliminary codes and categories. Coding was then discussed with site leads in Dar es Salaam (DS: social science) and Nottingham (MS: clinical psychology), following which a preliminary coding framework for implementation influences was developed. The codebook was then transferred into NVivo 12 software for coding. MR and AC independently coded the same four transcripts, and then discussed and reviewed any differences or discrepancies and any additional themes that emerged from the data. Following review, refinement, and defining of themes, an agreement was reached and new codes were incorporated into the final coding framework. The remaining transcripts were then coded with repeated discussion between coders. The finalised coding framework was iteratively discussed amongst the four primary analysts (AC, MR, MS, and DS) and the wider author team until a consensus was reached.

For peer review only - http://bmjopen.bmj.com/site/about/guidelines.xhtml

Results
The conceptual framework is shown in Table 1.

Insert Table 1 here

A total of 84 individuals participated, as shown in Table 2.

Insert Table 2 here

The codebook used for thematic analysis is shown in Table 3.

Insert Table 3 here

The finalised coding framework comprised seven implementation influence themes organised into societal (community attitudes; resource availability), organisational (organisational culture; staff attitudes; role definition) and PSW level (training and support; and peer support network).

Theme 1: Community attitudes
Community attitudes toward mental illness were perceived to be both a barrier and also a facilitator for PSWs to perform their roles. Some participants reported that people with mental health conditions are considered inferior and are also rejected, thus making it difficult for the PSWs to perform their roles effectively.

> *When it comes to class, mental health patients are considered second hand, third hand or fourth hand citizens. So we are marginalised among the marginalised. We take the lowest rank status point in the community.* [#9, Kampala, PSW]

Sometimes the PSWs are rejected when they go to visit service users.

> *We are rejected, you can go to that person's place who may not wish to see you and they don't welcome you and you can't insist. Sometimes they just avoid you.* [#12, Kampala, PSW]

Religious beliefs can also act as a barrier in implementing peer support work.

> *There are so many religious leaders who believe that God doesn't fail. They interfere with our work. They stop our patients from taking medicine and they say that God is going to perform miracles then in the end they relapse. The traditional healers believe that mental illness is caused by traditional issues and they don't need Western medicine, they need herbs.* [#26, Kampala, PSW]

Positive community initiatives have facilitated implementation. These initiatives have enabled the PSWs to be known as role models in the community and have inspired hope to others. Additionally, the notion of knowledge from experience adds value to the potential contribution of the PSW and helps transform and enhance the value of lived experience.

> *Now that we are role-models in the community people inquire from us about the things which I did to enable me to stabilize while at first they were stigmatizing me. They were beating me but now it is in their families and they are having issues worse than mine. They*

For peer review only - http://bmjopen.bmj.com/site/about/guidelines.xhtml

are like 'you see that mentally sick lady who was here? She is now stable. Let us go and inquire from her so that she can help us'…So we have become brokers in the village. [#14, Kampala, PSW]

Theme 2: Resource availability

Resource availability was an implementation barrier. Participants in lower resource settings described a lack of funding or limited amount of fund to enable PSWs to effectively implement their roles.

Sometimes the money we get is not enough to cater for these costs such as airtime and transport, you also need money to cater for yourself when you are in the field but sometimes it is little. [#14, Kampala, PSW]

According to the inflation in the country the money can't be enough to move to a community. Sometimes you need to buy airtime to call a peer, you have to fix something or food in the community, you have to get something to drink or eat in the community. Sometimes you find that this peer you are visiting is far away from where you stay, so the money we are paid is not enough. [#10, Kampala, PSW]

Participants from higher resource settings also reported that, whilst PSWs are an important component of mental health services, there is a limited budget set for them and there is a particular challenge in relation to funding arrangements for the peer support program.

The current policy needs to change. The policy says peer specialists have to be everywhere…and it requires resources which we don't have. I am not sure if I can raise the issue but we don't have budgets like the welfare…We need to get a special budget for the program. [#6, Be'er Sheva, Mental health clinician]

Then there is always the question of who finances it. For example the peer support workers on the ground floor (acute ward), are they financed by the ward budget or hospital budget or are they somehow cross-financed by other projects? [#3, Hamburg, Mental health clinician]

The facilities available to enable PSWs to perform their roles can be inadequate. It was noted that the working environment in lower-resource health facilities is very poor, exposing the PSWs, mental health workers and services users to many risks.

Our buildings do not have emergency exits, for example at the inpatient when the patient comes out it might be dangerous for the nurse and the nurse can get killed…The…Outpatient department can only accommodate four people while there are almost a hundred or ninety people per day so you find that people are just standing. [#1, Dar es Salaam, Mental health clinician]

We don't have enough facilities within the hospital, nurses face some challenges also. When you go to [name] ward, some peers are sleeping down and even divide blankets. You may find that one blanket is divided among 2 to 4 patients. [#13, Kampala, PSW]

Theme 3: Organisational culture

The culture, including goals, attitudes, role assumptions and values, of the organisation employing PSWs was a barrier to implementation. Participants reported that working inside structured and

For peer review only - http://bmjopen.bmj.com/site/about/guidelines.xhtml

hierarchal systems can create a feeling of indebtedness to the organisation which can impact on PSWs autonomy in decision-making and contribute towards feelings of disempowerment.

> *When you enter a job as a consumer provider, at least in the beginning, there is part of you that feels like they are doing you a favour that they hired you. That you have to do what the organisation tells you to do in order to gain experience etc.* [#8, Be'er Sheva, PSW]

Participants highlighted that the formalisation of the PSW role in such defined systems raises the question of how much of the role should remain informal versus formal in order for PSWs to fit in.

> *I also think that what especially happens with peer specialists, is some sort of formalization of this thing, and how much do we really actually want to formalize it. And how much of it do we have to keep informal, which is one of the worries or dilemmas. The ideals that are really inside of this system that is so formal, and is hierarchical and clear.* [#3, Be'er Sheva, PSW]

Participants reported that the support can be very limited due to the lack of psychological and social support resources available.

> *In summary, there is diagnosis, treatment mostly pharmacological using medical treatment. [We] do our best to try to provide psychological and social support but those are very limited most of the time.* [#30, Kampala, Manager]

Theme 4: Staff attitudes
Some staff have shown acceptance of PSW roles, whereas others are not willing to work with them as they consider them to be 'mad'.

> *Actually it has bridged a gap between service users and service providers. There is some kind of mutual understanding that we have built up. We are treated like staff.* [#14, Kampala, PSW]

> *And even here at [name] hospital some of the professionals say 'who can work with those mad ones?'. But some of them, those who accepted us are happy to work with us, they even smile at us and talk to us but there are others who think that mental illness is contagious.* [#5, Kampala, PSW]

Theme 5: Role definition
Having a clear role definition and expectations were reported as important, because without this reference point it can lead to potential role confusion and uncertainty.

> *I don't think it's that easy. They often don't know what they can do themselves. That they also have ideas, what can I actually do now? And I don't think there were enough guidelines or terms of reference.* [#11, Ulm, Mental health clinician]

In addition, the wide variety of tasks PSWs can perform means it can be difficult to construct a role description that accurately fits with real-life peer support practice.

> *The task fields of peer support workers are totally different. That is always person-dependent. We tried to create a kind of job description already and that was very, very*

For peer review only - http://bmjopen.bmj.com/site/about/guidelines.xhtml

difficult. Because we didn't want to restrict the peer support workers too much. Since the tasks always depend on the personality. [#2, Ulm, Mental health clinician]

Theme 6: Training and Support

The availability of training enables PSWs to know what is expected from them and also understand their needs.

> *The first thing is that they are supposed to receive is training. If they receive training they will know their job description and the techniques of going to the families because there are families which don't want people to know that they have a mental patient.* [#3, Dar es Salaam, Mental health clinician]

> *I can say that the PSW program that I was part of had PSWs who first of all received training especially to understand their needs, making sure they are dealing with mental illness of others and also how they work with PSWs.* [#30, Kampala, Manager]

Participants described how PSWs need support to maintain their wellbeing and carry out their roles.

> *They shouldn't always work in isolation; they should be supervised.* [#24, Kampala, Mental health clinician]

> *Peer support workers can't be independent, they need professional community nurses to guide them so they can go out in the field, they can be together.* [#30, Kampala, Manager]

> *Coming from a person who is currently dealing with very active symptoms with varying levels of force, a person needs…regulatory capacity, the ability to manage workloads, the ability to receive help, to be helped and to defend oneself.* [#1, Be'er Sheva, Mental health clinician]

Participants identified the need for initial and further training in boundaries, code of conduct, and levels of disclosure.

> *Peer support workers need more training, continuous training. Even if the training is a one-off. So this should be happening. It shouldn't be a big deal.* [#30, Kampala, Manager]

Theme 7: Peer support network

Through peer support networks, PSWs felt connected as they shared their experiences and they felt stronger together.

> *I can see how a group of peers impact each other non-stop and advances processes almost as if it is a race, but not in a bad way. That is to say, not from a place where you feel that they are forcing you to run, but from a place where a lot of people who are together all the time, are shattering stigmas about one another, I think that a group is stronger.* [#3, Be'er Sheva, PSW]

Through these networks PSWs get to know each other well, and can identify if another PSW was facing a problem.

For peer review only - http://bmjopen.bmj.com/site/about/guidelines.xhtml

We peers have what they call buddies. A buddy is a person who knows more about you whereby in case you show signs of relapse that buddy will say '(name) is getting a relapse, (name) do this and this'. He will help you and bring you medical personnel and overcome the situation. [#11, Kampala, PSW]

Discussion

Our study identified seven influences on PSW implementation. At the societal level, community stigma and lay beliefs about mental conditions were influential. At the organisational level, the inter-linked themes of resource allocation and organisational culture were identified, as well as staff attitudes and the challenge of role clarity. At the PSW level, both adequate training /support and a strong peer support network were facilitators of implementation.

The strengths of the study include the sample size, the use of multiple informants, conducting local language topic guides to avoid excluding non-English speaking participants, and the multi-national sampling frame. Credibility of the findings was enhanced by independent coding and the use of multiple analysts. Several limitations can be identified. One significant shortcoming is that sociodemographic characteristics of participants were not collected in a standardised way across all sites, limiting the transferability of findings. Whilst the sample is large for a qualitative study, the findings are complex and nuanced, so our analysis focused on semantic rather than latent coding[27]. Future analysis might explore the relationship between the identified implementation influences, such as how community attitudes may distally impact on resource allocation. Whilst the use of analysts with different professional backgrounds reduced researcher influence on findings, the credibility of the findings could be enhanced by member checking, and including people with lived experience as co-analysts.[28] Finally, the relatively small number of policy-maker participants may account for the limited mention of national and regional policy as an influence.

Two aspects of our findings are noteworthy in relation to other studies. First, the conceptual framework was developed on the basis of research almost exclusively from high-income countries, and identified PSW and organisational influences. The implementation influences identified by participants had a stronger emphasis on societal aspects, including attitudes and role assumptions. Our findings are consistent with the previously-discussed systematic review,[21] published since the conceptual framework was developed. This validates the importance of considering organisational and specifically societal aspects when implementing PSW in different resource settings. This involves developing community awareness regarding the value of peer support, to gain the support of family and community members.[29] Second, the PSW-level influences indicate the need to modify how PSW is provided in different settings. A systematic review of 39 studies, only one from a lower income setting, identified seven types of modification to the PSW role,[30] including recruitment processes, role expectations, training and support. Recent research is expanding to also consider staff attitudes,[31] organisational integration of PSWs,[32,33] organisational climate[34] and context.[35,36]

The primary implication is that more attention needs to be paid to societal attitudes and organisational culture in developing and implementing PSW programmes. Discrimination and stigma relating to mental health are global challenges,[4] but our findings suggest that there is a relationship between community attitudes and the ability to involve people with lived experience in the mental health workforce as PSWs. In terms of organisational culture, the findings reinforce existing evidence[37] that organisational culture impacts on recovery support, so organisational transformation may be needed.[38] Approaches to supporting culture change within mental health services include the introduction of pro-recovery interventions,[39,40] development of adjunctive

For peer review only - http://bmjopen.bmj.com/site/about/guidelines.xhtml

services such as Recovery Colleges,[41] working with teams[42] and introducing co-production[43] and growth-oriented approaches.[44]

A better understanding of the relationship between the identified influences is needed. In UPSIDES, the theory of change technique is being used to map out different steps in the implementation of the PSW intervention, and to articulate the connections between these steps. The impact of societal and organisational influences on PSW effectiveness will be illuminated in the multi-national UPSIDES randomised controlled trial (ISRCTN26008944) which is currently underway.[23]

For peer review only

For peer review only - http://bmjopen.bmj.com/site/about/guidelines.xhtml

Acknowledgements
The Using Peer Support In Developing Empowering Mental Health Services (UPSIDES) Study is a multicentre collaboration between the Department for Psychiatry and Psychotherapy II at Ulm University, Germany (Bernd Puschner, coordinator); the Institute of Mental Health at University of Nottingham, UK (Mike Slade); the Department of Psychiatry at University Hospital Hamburg-Eppendorf, Germany (Candelaria Mahlke); Butabika National Referral Hospital, Uganda (David Basangwa); the Centre for Global Mental Health at London School of Hygiene and Tropical Medicine, UK (Grace Ryan); the Department of Health Systems, Impact Evaluation and Policy, Ifakara Health Institute, Dar es Salaam, Tanzania (Donat Shamba); the Department of Social Work at Ben Gurion University of the Negev, Be'er Sheva, Israel (Galia Moran); and the Centre for Mental Health Law and Policy, Pune, India (Jasmine Kalha).

Contributors
MR, AC contributed to data collection, analysis and interpretation, and drafting the work. AG, RH contributed to interpretation. JK, CM, RM, GR, DS and MS contributed to the design of the work and data collection, acquisition, analysis and interpretation, and critically revising the work for important intellectual content. DS and MS contributed to the design of the work and data collection, acquisition, analysis and interpretation, and drafting the work. All authors gave final approval of the version to be published, and are accountable for all aspects of the work.

Funding
This work was supported by the European Union Horizon 2020 Research and Innovation Programme under Grant Agreement No 779263. MS acknowledges the support of the Center for Mental Health and Substance Abuse, University of South-Eastern Norway and the NIHR Nottingham Biomedical Research Centre. This publication reflects only the authors' views. The Commission is not responsible for any use that may be made of the information it contains. The funding body had no role in the design of the study and in writing the manuscript.

Competing Interests
None declared.

Data sharing statement
Full transcripts are not publicly available due to their containing information that could compromise the privacy of research participants.

For peer review only

References

1. Samele C, Frew, S., Urquia, N. European profile of prevention and promotion of mental health (EuroPoPP-MH): EU Health Programme [(EAHC/2010Health/04)]; 2013.
2. Pathare S, Brazinova A, Levav I. Care gap: a comprehensive measure to quantify unmet needs in mental health. *Epidemiology and Psychiatric Sciences* 2018; **27**: 463-7.
3. Collins PY, Patel V, Joestl SS, March D, Insel TR, Daar AS. Grand challenges in global mental health. *Nature* 2011; **475**(7354): 27-30.
4. Patel V, Saxena S, Lund C, et al. The Lancet Commission on global mental health and sustainable development. *Lancet* 2018; **392**: 1553-98.
5. Misra S, Stevenson A, Haroz E, de Menil V, Koenen K. 'Global mental health': systematic review of the term and its implicit priorities. *Br J Psychiatry* 2019; **5**: e47.
6. Davidson L, Bellamy C, Guy K, Miller R. Peer support among persons with severe mental illnesses: a review of evidence and experience. *World Psychiatry* 2012; **11**: 123-8.
7. Mead S. Intentional Peer Support: an alternative approach. Plainfield, NH: Shery Mead Consulting; 2005.
8. Pitt V, Lowe D, Hill S, et al. Consumer-providers of care for adult clients of statutory mental health services. *Cochrane Database of Systematic Reviews* 2013; **3**: CD004807.
9. Lloyd-Evans B, Mayo-Wilson E, Harrison B, et al. A systematic review and meta-analysis of randomised controlled trials of peer support for people with severe mental illness. *BMC Psychiatry* 2014; **14**: 39.
10. Shalaby R, Agyapong V. Peer Support in Mental Health: Literature Review. *JMIR Mental Health* 2020; **7**: e15572.
11. Chinman M, George, P., Dougherty, R., Daniels, A., Ghose, S., Swift, A., Delphin-Rittmon, M. Peer Support Services for Individuals With Serious Mental Illnesses: Assessing the Evidence. *Psychiatr Serv* 2014; **65**: 429-41.
12. White S, Foster R, Marks J, et al. The effectiveness of one-to-one peer support in mental health services: a systematic review and meta-analysis. *BMC Psychiatry* 2020; **20**: 534.
13. Repper J. ImROC Briefing Paper 5. Peer Support Workers: Theory and Practice. London: Centre for Mental Health; 2013.
14. Mead S, Hilton, D., Curtis, L. Peer support: A theoretical perspective. *Psychiatric Rehabilitation Journal* 2001; **25**: 134–41.
15. Fan Y, Ma N, Ma L, Xu W, Lamberti JS, Caine E. A community-based peer support service for persons with severe mental illness in China. *BMC Psychiatry* 2018; **18**: 170.
16. Pathare S, Kalha J, Krishnamoorthy S. Peer support for mental illness in India: An underutilised resource. *Epidemiology and Psychiatric Sciences* 2018; **27**: 415-9.
17. Moran GS. The mental health consumer movement and peer providers in Israel. *Epidemiology and Psychiatric Sciences* 2018; **27**: 420-6.
18. Lee YY, Ang S, Chua HC, Subramaniam M. Peer Support in Mental Health: A Growing Movement in Singapore. *Annals of Academy of Medicine Singapore* 2019; **48**: 95-7.
19. Ryan G, Kamuhiirwa M, Mugisha J, et al. Peer support for frequent users of inpatient mental health care in Uganda: protocol of a quasi-experimental study. *BMC Psychiatry* 2019; **19**: 374.
20. Piat M, Sofouli E, Sabetti J, et al. Protocol for a mixed studies systematic review on the implementation of the recovery approach in adult mental health services. *BMJ Open* 2017; **7**(8).
21. Ibrahim N, Thompson D, Nixdorf R, et al. A systematic review of influences on implementation of peer support work for adults with mental health problems. *Soc Psychiatry Psychiatr Epidemiol* 2020; **55**: 285-93.
22. Puschner B, Repper J, Mahlke C, et al. Using Peer Support in Developing Empowering Mental Health Services (UPSIDES): Background, rationale and methodology. *Annals of Global Health* 2019; **85**(53): 1-10.

For peer review only - http://bmjopen.bmj.com/site/about/guidelines.xhtml

23. Moran G, Kalha J, Mueller-Stierlin A, et al. Peer support for people with severe mental illness versus usual care: study protocol for a pragmatic multicentre randomised controlled trial (UPSIDES-RCT). *Trials* 2020; **21**: 371.
24. Mahlke C, Nixdorf R, Repper J, et al. UPSIDES Peer Support Worker Training Manual and Workbook: UPSIDES Study; 2020.
25. Jabareen Y. Building a Conceptual Framework: Philosophy, Definitions, and Procedure. *International Journal of Qualitative Methods* 2009; **8**: 49-62.
26. Repper J, Perkins R. ImROC Briefing Paper 6. The Team Recovery Implementation Plan: a framework for creating recovery-focused services. London: Centre for Mental Health; 2013.
27. Braun V, Clarke V, Hayfield N, Terry G. Thematic analysis. In: Laiamputtong L, ed. Handbook of Research Methods in Social Sciences. Singapore: Springer; 2019: 843-60.
28. Jennings H, Slade M, Bates P, Munday E, Toney R. Best practice framework for Patient and Public Involvement (PPI) in collaborative data analysis of qualitative mental health research: methodology development and refinement. *BMC Psychiatry* 2018; **18**: 213.
29. Pearson H, Adams B, Eaton J, Illingworth M. Community Mental Health Good Practice Guide: Peer Support: CBM Global Disability Inclusion; 2021.
30. Charles A, Thompson D, Nixdorf R, et al. A typology of modifications to peer support work for adults with mental health problems: systematic review. *Br J Psychiatry* 2020; **216**: 301-7.
31. Moore T, Zeeman L. More ‘milk’ than ‘psychology or tablets’: Mental health professionals’ perspectives on the value of peer support workers. *Health Expectations* DOI: 10.1111/hex.13151.
32. Charles A, Nixdorf R, Ibrahim N, et al. Initial training for mental health peer support workers: international Delphi Consultation. *JMIR Mental Health* 2021; **5**: e25528.
33. Otte I, Werning A, Nossek A, Vollmann J, Juckel G, Gather J. Challenges faced by peer support workers during the integration into hospital-based mental health-care teams: Results from a qualitative interview study. *Int J Soc Psychiatry* 2020; **66**: 263-9.
34. Jones N, Teague G, Wolf J, Rosen C. Organizational Climate and Support Among Peer Specialists Working in Peer-Run, Hybrid and Conventional Mental Health Settings. *Administration and Policy in Mental Health and Mental Health Services Research* 2020; **47**: 150-67.
35. Adams W. Unintended consequences of institutionalizing peer support work in mental healthcare. *Soc Sci Med* 2020; **262**: https://doi.org/10.1016/j.socscimed.2020.113249.
36. Urichuk L, Hrabok M, Hay K, et al. Enhancing peer support experience for patients discharged from acute psychiatric care: protocol for a randomised controlled pilot trial. *BMJ Open* 2018; **8**: e022433.
37. Clossey L, Rheinheimer D. Exploring the effect of organizational culture on consumer perceptions of agency support for mental health recovery. *Community Ment Health J* 2014; **50**(4): 427-34.
38. Le Boutillier C, Chevalier A, Lawrence V, et al. Staff understanding of recovery-orientated mental health practice: a systematic review and narrative synthesis. *Implementation Science* 2015; **10**: 87.
39. Meadows G, Brophy L, Shawyer F, et al. REFOCUS-PULSAR recovery-oriented practice training in specialist mental health care: a stepped-wedge cluster randomised controlled trial. *Lancet Psychiatry* 2019; **6**: 103-14.
40. Slade M, Bird V, Clarke E, et al. Supporting recovery in patients with psychosis through care by community-based adult mental health teams (REFOCUS): a multisite, cluster, randomised, controlled trial. *Lancet Psychiatry* 2015; **2**: 503-14.
41. Crowther A, Taylor A, Toney R, et al. The impact of Recovery Colleges on mental health staff, services and society. *Epidemiology and Psychiatric Sciences* 2019; **28**: 481-8.

For peer review only - http://bmjopen.bmj.com/site/about/guidelines.xhtml

42. Vacher G. Utilising Team Recovery Implementation Plan (TRIP): embedding recovery-focused practice in rehabilitation services. *Mental Health and Social Inclusion* 2017; **21**: 240-7.
43. Lewis A, King T, Herbert L, Repper J. Co-Production – Sharing Our Experiences, Reflecting On Our Learning. Nottingham: ImROC; 2017.
44. Slade M, Rennick-Egglestone S, Blackie L, et al. Post-traumatic growth in mental health recovery: qualitative study of narratives. *BMJ Open* 2019; **9**: e029342.

For peer review only

For peer review only - http://bmjopen.bmj.com/site/about/guidelines.xhtml

Table 1: Conceptual framework for PSW principles (n=8) and societal / organisational implementation influences (n=5)

Principle	Definition
1. Mutual	Peer support workers have similar experiences to peer support users
2. Reciprocal	Peer support workers and peer support users both give and receive in the relationship
3. Non-directive	Peer support workers develop solutions together with the peer support user, instead of dictating solutions
4. Recovery focused	Peer support workers support the peer support user on his/her path towards overcoming the problems that they experience
5. Strengths-based	Peer support workers show a positive attitude and identify and build on the strengths and recovery progress of peer support users
6. Inclusive	Peer support workers do not exclude people on the basis of the nature of their problems or beliefs about their level of ability, and help peer support users to find their place in society
7. Progressive	Peer support workers and users advance together towards recovery, this is not a befriending relationship that aims to maintain current progress
8. Safe	Peer support workers and users develop a common basis of trust and safety, which is central to the planning of the service and training of peer workers
**Implementation influence**	**Description of societal / organisational influence**
1. Group versus individual	Peer support can be offered in single sessions and in a group setting
2. Extent to which both parties choose to enter the relationship	Peer support pairs and groups can be formed by the organisation, but also by the peers themselves
3. Extent to which rules govern the relationship	There can be implicit and explicit rules underpinning how the peer support work is conducted
4. Extent to which the parties involved are in the same place in their recovery journey	Depending on the state of recovery, peer support users can become peer support workers and vice versa
5. Extent to which the peer support workers focus on peer support users	Peer support workers can support recovery for peer support users and/or promote a recovery orientation for the staff they work with, the institution they work in, and the society they live in

For peer review only

For peer review only - http://bmjopen.bmj.com/site/about/guidelines.xhtml

Table 2: Focus group participants (n=84)

Site	Focus groups	N	Participant				Gender	
			Potential or actual PSW	Mental health worker	Mental health manager	Policy-maker	Male	Female
Ulm	2	12	1	10	0	1	4	8
Hamburg	2	12	7	5	0	0	4	8
Kampala	4	32	16	13	1	0	10	22
Dar es Salaam	2	16	0	12	4	0	7	9
Be'er Sheva	2	14	2	8	4	0	5	9
**Total**	**12**	**86**	**26**	**48**	**9**	**1**	**30**	**56**

For peer review only - http://bmjopen.bmj.com/site/about/guidelines.xhtml

Table 3: Final codebook for implementation influences

Code	Definition
**1. Societal level**	**Political, cultural, and social factors influencing the implementation of mental health (MH) peer support work (PSW) interventions, including income levels**
1.1 MH policy	The national structures, guidelines, health and social care policy and strategic importance placed on MH services
1.2 PSW policy	Local and national PSW policies and structures, including the strategic importance attached to PSW roles
1.3 Positive community initiatives	Approaches which raise community awareness and educate people about MH and PSW, including through arts, education, media, community projects and religious institutions
1.4 Negative community attitudes	The negative ways in which individuals with MH conditions are perceived by others, and the implications of stigma such as feelings of isolation, lack of self-esteem, and the need for strong advocacy
1.5 Help-seeking	Culturally-sanctioned help-seeking behaviour in the community, including accessing the MH system but also traditional healers, shrines, religious institutions (e.g. churches, mosques) and informal peer networks such as WhatsApp groups
**2. Organisational level**	**Service and organisational level factors influencing the implementation of PSW interventions in the MH system**
2.1 Organisational culture	The goals, attitudes, role assumptions and values held in an organisation about PSW, and the relationship between the PSW and the organisation
2.2 Financial resources	The financial resources available in the organisation for PSWs to perform their role, including remuneration, transport, and catering
2.3 Non-financial resources	The facilities, working environment, and infrastructure resources available in the organisation for PSWs to perform their role, including built environment, staffing, translators, medicine, community services, tracing systems
2.4 Care processes	Availability of MH care within services, including medical and psychosocial interventions, management processes, health education, admission and discharge processes
2.5 Staff attitudes	Non-peer MH worker willingness, acceptance, and ability to work with PSWs
2.6 PSW Recruitment	The recruitment processes used by organisations when selecting prospective individuals for a PSW role, including group interviews, courses, reviewing individual files, conducting tests, and checking urine for substance use
2.7 PSW initial training	PSW initial training availability and content, including wellbeing, crisis management, working with service users in distress, their families, other MH workers, PSWs, and opportunities for ongoing training
2.8 Role support	The support needed for PSWs to maintain their wellbeing and carry out their role, including supervision, guidelines, and support from other MH workers
**3. PSW level**	**PSW-level factors influencing role performance, including job description, expectations, knowledge, and interpersonal skills**
3.1 Role definition	The extent to which there is a clearly-defined PSW role, and other MH workers understanding of the role. In addition, PSWs have training about boundaries, code of conduct, and levels of self-disclosure
3.2 PSW support networks	PSW access to support from other peers and informal networks

For peer review only

Instructions:

This guide is to be used to facilitate focus group discussion with (1.1) health workers who are using or plan to recruit peer support workers. (Psychiatric nurses, clinicians working in mental health ward / departments) and (1.2) local stakeholders with relevant expertise relating to implementation of peer work (including clinicians and managers who currently, previously or in the future may employ peer support workers, and people who currently, previously or in the future may work as peer support workers).

Interviewer ID ___ ___ ___ ___

Interview date (DD/MM/YYYY): __ __ / __ __ / __ __ __

Location of the interview ____________________________________

Country: ____________________________________

Introduction:

Ensure that the participants are comfortable.

Hi and welcome to this session. My name is ___ and my colleague is___. Thank you for taking your time to talk to us about your experiences with people with severe mental illness and peer support workers. We will discuss topics related to the key characteristics of peer support workers and the challenges they may face. The discussion will take around 60minutes. The interview will be recorded using an audio recorder as we will not have to write down all your answers and we will not miss any of your useful comments. Informed consent will be obtained from all the participants and confidentiality will be assured to all the participants. Before we begin, I would like to know if you have any questions.

Explain you are starting the audio recorder.

Time interview started:

Theme 1: Socio-demographic information of the respondents

Collect data on name, age, education, position and number of years working in this position and give a code to each participant.

Explain again to the participant that:

- I want to learn about your experience, thoughts and perspective on this topic of using peer support workers for improving mental health conditions in your country.
- There is no 'right' or 'wrong' answer.

Say: We are planning to recruit and train peer support workers. Peer providers in mental health are individuals with severe mental illness who are further along in their recovery, who support others with similar conditions by role modelling that recovery is possible, sharing knowledge from experience and using reciprocal empathic relationships. We are thinking of asking these peer support workers to identify and visit individuals with severe mental illness. We would like to get your opinion on how best to implement PSWs intervention in this region. We would like to know key characteristics for PSWs and challenges that could hinder them performing their

For peer review only

UPSIDES

tasks. Please be frank as we want the information to be the right ones for this community.

Theme 2: Social cultural factors and acceptability of PSWs

1. Based on your experiences, how does the community care for people with SMIs? Where do they seek care? Are there other places apart from the hospital? Describe all the places people seek care for SMI.

2. What are your main roles in supporting people with SMIs? What are the main challenges that you are facing in doing your job? What are the solutions to those challenges?

   - Probe to know if mental health programmes are given a priority?

   - Is there a national policy or guideline for mental health conditions? Are these accessible?

   - What has been the main challenge your department has been facing in managing/meeting the needs of people with Severe Mental Illness? Probe for various challenges the department has been facing in regard to SMI programmes? For each challenge mentioned, ask how the department could handle it i.e. any potential solution and/or suggestion on how to address it?

3. With your experience working in this area, could you please tell us what do you know about PSWs and how do they perform their duties?

   - Probe for, how they are recruited, who recruits them, what are they exactly doing, etc?

   - Please, describe any systems for linking people with peers who can serve as role models in this facility or community? (e.g. through contact with local user-run groups).

4. What key qualities would you want a peer support worker to have or NOT to have? We want to find out what sort of person the respondents would trust and value, perform the work well and responsibly.

5. Could you please tell us what are the specific things, you would want a peer support worker to do?

   Probe for specific terminologogies, main activities, duration of activities. care planning

Theme 3: Institution / facility readiness to incorporate PSW

6. In your opinion – for using peer support workers, how could it be implemented / improved at this facility?

For peer review only

- Probe: training, logistics, management, supervision, monitoring, capacity development, resources etc.

7. What are the facilitators and barriers in providing peer support to people with SMI in your facility or in your region?

   - Probe for resources, skilled personnel, infrastructure, systems support?

8. Is there anything more you would like to add about your experiences with, or views on, using peer support workers?

Time interview end:

Thank the participant for his / her time. Remind them that the information will be kept confidential.

Interviewer comments on how the interview went:

For peer review only

For peer review only - http://bmjopen.bmj.com/site/about/guidelines.xhtml

Standards for Reporting Qualitative Research Checklist

Item	Topic	Where addressed
**Title and abstract**		
S1	**Title** Concise description of the nature and topic of the study Identifying the study as qualitative or indicating the approach (e.g., ethnography, grounded theory) or data collection methods (e.g., interview, focus group) is recommended	Title
S2	**Abstract** Summary of key elements of the study using the abstract format of the intended publication; typically includes background, purpose, methods, results, and conclusions	Abstract
**Introduction**		
S3	**Problem formulation** Description and significance of the problem/phenomenon studied; review of relevant theory and empirical work; problem statement	Introduction
S4	Purpose or research question Purpose of the study and specific objectives or questions	Introduction final paragraph
**Methods**		
S5	**Qualitative approach and research paradigm** Qualitative approach (e.g., ethnography, grounded theory, case study, phenomenology, narrative research) and guiding theory if appropriate; identifying the research paradigm (e.g., postpositivist, constructivist/interpretivist) is also recommended; rationale	Methods Design Method Procedures paragraph 2 Methods Analysis
S6	**Researcher characteristics and reflexivity** Researchers' characteristics that may influence the research, including personal attributes, qualifications/experience, relationship with participants, assumptions, and/or presuppositions; potential or actual interaction between researchers' characteristics and the research questions, approach, methods, results, and/or transferability	Methods Analysis Discussion paragraph 2
S7	**Context**	Methods Setting

For peer review only

	Setting/site and salient contextual factors; rationale	
S8	**Sampling strategy**<br>How and why research participants, documents, or events were selected; criteria for deciding when no further sampling was necessary (e.g., sampling saturation); rationale	Methods Participants<br>Methods Procedures final paragraph<br>Methods Sampling
S9	**Ethical issues pertaining to human subjects** Documentation of approval by an appropriate ethics review board and participant consent, or explanation for lack thereof; other confidentiality and data security issues	Methods Ethics approval
S10	**Data collection methods** Types of data collected; details of data collection procedures including (as appropriate) start and stop dates of data collection and analysis, iterative process, triangulation of sources/methods, and modification of procedures in response to evolving study findings; rationale. The rationale should briefly discuss the justification for choosing that theory, approach, method, or technique rather than other options available, the assumptions and limitations implicit in those choices, and how those choices influence study conclusions and transferability. As appropriate, the rationale for several items might be discussed together.	Methods Procedures<br>Methods Analysis
S11	**Data collection instruments and technologies**<br>Description of instruments (e.g., interview guides, questionnaires) and devices (e.g., audio recorders) used for data collection; if/how the instrument(s) changed over the course of the study	Methods Procedures paragraphs 1 and 2
S12	**Units of study**<br>Number and relevant characteristics of participants, documents, or events included in the study; level of participation (could be reported in results)	Results Table 1
S13	**Data processing**<br>Methods for processing data prior to and during analysis, including transcription, data entry, data management and security, verification of data integrity, data coding, and anonymization/deidentification of excerpts	Methods Procedures final paragraph
S14	**Data analysis**<br>Process by which inferences, themes, etc., were identified and developed, including the researchers involved in data analysis; usually references a specific paradigm or approach; rationale	Methods Analysis

For peer review only

S15	**Techniques to enhance trustworthiness**<br>Techniques to enhance trustworthiness and credibility of data analysis (e.g., member checking, audit trail, triangulation); rationale	Methods Analysis Discussion strengths and limitations paragraph
**Results/findings**		
S16	**Synthesis and interpretation**<br>Main findings (e.g., interpretations, inferences, and themes); might include development of a theory or model, or integration with prior research or theory	Results
S17	**Links to empirical data** Evidence (e.g., quotes, field notes, text excerpts, photographs) to substantiate analytic findings	Results
**Discussion**		
S18	**Integration with prior work, implications, transferability, and contribution(s) to the field**<br>Short summary of main findings; explanation of how findings and conclusions connect to, support, elaborate on, or challenge conclusions of earlier scholarship; discussion of scope of application/generalizability; identification of unique contribution(s) to scholarship in a discipline or field	Discussion
S19	**Limitations**<br>Trustworthiness and limitations of findings	Discussion paragraph 2
**Other**		
S20	**Conflicts of interest**<br>Potential sources of influence or perceived influence on study conduct and conclusions; how these were managed	Competing Interests statement
S21	**Funding**<br>Sources of funding and other support; role of funders in data collection, interpretation, and reporting	Funding statement

For peer review only

BMJ Open

Societal and organisational influences on implementation of mental health peer support work in low-income and high-income settings: qualitative focus group study

Journal:	*BMJ Open*
Manuscript ID	bmjopen-2021-058724.R1
Article Type:	Original research
Date Submitted by the Author:	19-Apr-2022
Complete List of Authors:	Ramesh, Mary; Ifakara Health Institute, Department of Health System, Impact Evaluation and Policy<br>Charles, Ashleigh; University of Nottingham, School of Health Sciences<br>Grayzman, Alina; Ben-Gurion University of the Negev Faculty of Health Sciences, Department of Social Work, Ben Gurion University of the Negev<br>Hiltensperger, Ramona; Department of Psychiatry II<br>Kalha, Jasmine; Indian Law Society<br>Kulkarni, Arti; Indian Law Society<br>Mahlke, Candelaria ; University Medical Cente Hamburg, Candelaria Irene Mahlke<br>Moran, Galia; Ben-Gurion University of the Negev, Department of Social Work<br>Mpango, Richard; Butabika National Referral Hospital; Soroti University<br>Stierlin, Annabel; Ulm University, Institute of Epidemiology and Medical Biometry<br>Nixdorf, Rebecca ; University Medical Centre Hamburg-Eppendorf, Department of Psychiatry<br>Ryan, Grace; London School of Hygiene and Tropical Medicine, Population Health<br>Shamba, Donat ; Ifakara Health Institute, Department of Health Systems, Impact Evaluation and Policy<br>Slade, Mike; University of Nottingham School of Health Sciences
**Primary Subject Heading**:	Global health
Secondary Subject Heading:	Global health, Mental health, Qualitative research
Keywords:	MENTAL HEALTH, QUALITATIVE RESEARCH, PUBLIC HEALTH

SCHOLARONE™
Manuscripts

I, the Submitting Author has the right to grant and does grant on behalf of all authors of the Work (as defined in the below author licence), an exclusive licence and/or a non-exclusive licence for contributions from authors who are: i) UK Crown employees; ii) where BMJ has agreed a CC-BY licence shall apply, and/or iii) in accordance with the terms applicable for US Federal Government officers or employees acting as part of their official duties; on a worldwide, perpetual, irrevocable, royalty-free basis to BMJ Publishing Group Ltd ("BMJ") its licensees and where the relevant Journal is co-owned by BMJ to the co-owners of the Journal, to publish the Work in this journal and any other BMJ products and to exploit all rights, as set out in our licence.

The Submitting Author accepts and understands that any supply made under these terms is made by BMJ to the Submitting Author unless you are acting as an employee on behalf of your employer or a postgraduate student of an affiliated institution which is paying any applicable article publishing charge ("APC") for Open Access articles. Where the Submitting Author wishes to make the Work available on an Open Access basis (and intends to pay the relevant APC), the terms of reuse of such Open Access shall be governed by a Creative Commons licence – details of these licences and which Creative Commons licence will apply to this Work are set out in our licence referred to above.

Other than as permitted in any relevant BMJ Author's Self Archiving Policies, I confirm this Work has not been accepted for publication elsewhere, is not being considered for publication elsewhere and does not duplicate material already published. I confirm all authors consent to publication of this Work and authorise the granting of this licence.

For peer review only

Societal and organisational influences on implementation of mental health peer support work in low-income and high-income settings: qualitative focus group study

Mary Ramesh [1 a]
Ashleigh Charles [2 a]
Alina Grayzman [3]
Ramona Hiltensperger [4]
Jasmine Kalha [5]
Arti Kulkarni[5]
Candelaria Mahlke [6]
Galia Moran[3]
Richard Mpango [7 8 9]
Annabel Sandra Müller-Stierlin [4]
Rebecca Nixdorf [6]
Grace Ryan [10]
Donat Shamba [1 b *]
Mike Slade [2 b]

[1] Department of Health Systems, Impact Evaluation and Policy, Ifakara Health Institute, Dar es Salaam, Tanzania
[2] School of Health Sciences, Institute of Mental Health, University of Nottingham, UK
[3] Department of Social Work, Ben Gurion University of the Negev, Be'er Sheva, Israel
[4] Department of Psychiatry II, Ulm University, Ulm, Germany
[5] Centre for Mental Health Law and Policy, Indian Law Society, Pune, India
[6] Department of Psychiatry, University Medical Center Hamburg-Eppendorf, Germany
[7] Butabika National Referral Hospital, Uganda
[8] School of Health Sciences, Soroti University, Soroti, Uganda
[9] MRC/UVRI and LSHTM Uganda Research Unit, Entebbe, Uganda
[10] Centre for Global Mental Health, London School of Hygiene and Tropical Medicine, UK

[a] Ramesh and Charles contributed equally and are joint first authors
[b] Shamba and Slade contributed equally and are joint last authors

* Correspondence to: Donat Shamba: dshamba@ihi.or.tz

ORCID iDs
MR: 0000-0002-4942-3923; AC: 0000-0003-2222-4358; AG 0000-0002-8192-7697; RH: 0000-0003-2544-4188; JK: 0000-0001-7357-2366; AK: 0000-0002-3281-4388; CM: 0000-0001-9573-6106;GM: 0000-0001-9718-1773; RM: 0000-0001-6960-3174; AMS: 0000-0003-2812-5115; RN: 0000-0002-3064-8380; GR: 0000-0002-9310-3513; DS: 0000-0001-7431-7199; MS: 0000-0001-7020-3434

Word count
4,000 words.

For peer review only - http://bmjopen.bmj.com/site/about/guidelines.xhtml

ABSTRACT

Objectives

Despite the established evidence base for mental health peer support work, widespread implementation remains a challenge. This study aimed to explore societal and organisational influences on the implementation of peer support work in low and high income settings.

Design

Study sites conducted two focus groups in local languages at each site, using a topic guide based on a conceptual framework describing eight peer support worker principles and five implementation issues. Transcripts were translated into English and an inductive thematic analysis was conducted to characterise implementation influences.

Setting

The study took place in five secondary mental health care sites as part of the Using Peer Support in Developing Empowering Mental Health Services (UPSIDES) study, comprising three high-income sites (Hamburg and Ulm, Germany; Be'er Sheva, Israel) and two low-income sites (Dar es Salaam, Tanzania; Kampala, Uganda) chosen for diversity both in region and in experience of peer support work.

Participants

86 participants (56 females, 30 male) met inclusion criteria: aged over 18 years; actual or potential peer support worker (n=26) or mental health clinician (n=49) or hospital / community manager or regional / national policy-maker (n=11); able to give informed consent.

Results

Six themes relating to implementation influences were identified: community and staff attitudes; resource availability; organisational culture; staff attitudes; role definition; training and support; and peer support network.

Conclusions

This is the first multi-country study to explore societal attitudes and organisational culture influences on the implementation of peer support. Addressing community-level discrimination and developing a recovery orientation in mental health systems can contribute to effective implementation of peer support work. The relationship between societal stigma about mental health and resource allocation decisions warrants future investigation.

Trial registration

UPSIDES RCT: ISRCTN26008944

Strengths and limitations of this study

- The sample size (n=86) and sampling across two low-income and three high-income sites increases the credibility of the findings and their relevance for both low- and high-income settings.
- Independent coding by multiple analysts from different cultures enhances trustworthiness.
- Sociodemographic characteristics were not sufficiently collected to be reported, limiting transferability of findings.
- Saturation may not have been reached with two FGDs per site, there is a need to conduct further studies with service users or recipients of peer support services.

For peer review only

Introduction

Many people living with severe mental illness do not receive adequate care. For example, in Europe the EuroPoPP-MH study found that a comprehensive range of community-based services existed in only eight of 29 countries.[1] The resulting gap between demand and supply is called the treatment gap, or care gap.[2] While mental health has been identified as a global priority,[3] the mental health treatment gap remains, and is largest in low-resource settings.[4]

One reason for the treatment gap in low-resource settings is that global mental health initiatives do not sufficiently address contextual aspects, such as up-stream social determinants, geographic and linguistic differences, and sociodemographic influences such as ethnicity, caste and tribe.[5] This leads to barriers in receiving mental health treatment, including stigma, social exclusion and differing availability of resources.

Mental health peer support is an established intervention involving a person with lived experience of mental health problems and recovery employed to offer support to others with mental health problems. Peer support workers (PSWs) act as credible role models of recovery,[6] instilling hope through positive self-disclosure, modelling the use of experiential knowledge for self-care, and offering supportive relationships based on intentional mutuality.[7] There is a strong empirical evidence base for PSWs.[8-11] The most recent systematic review identified 19 randomised controlled trials,[12] all from high income countries. This review found PSW was associated with beneficial outcomes in relation to supporting recovery, empowerment and social networks. However, heterogeneity in the implementation of peer support was identified as an important knowledge gap.

Most research on mental health peer support work has been conducted in high-resource settings, including creation of core PSW principles[13,14] and evaluation.[8] However, PSW roles are increasingly being developed, formalised, and implemented in more diverse settings, such as China,[15] India,[16] Israel,[17] Singapore[18] and Uganda.[19] An important knowledge gap therefore exists in relation to PSW implementation influences across settings with different resource levels.[20] A recent systematic review synthesised 53 studies to identify 14 influences on implementation of mental health PSW.[21] The most commonly influence was organisational culture, identified in 53% of studies, training, and role definition. Societal influences were also identified, including PSW access to a peer network, resource availability and financial arrangements.

The Grand Challenges for Mental Health initiative identified the importance of research along the translation continuum including implementation, and emphasised that implementation is a challenge not just in low and middle income countries.[3] In other words, the focus should be on implementation research including both lower and higher resource settings. To our knowledge, no study has explored PSW implementation across multiple countries. The aim of this study was to explore and characterise the societal and organisational influences on the implementation of mental health peer support work in lower income and higher income settings.

Methods

This study was conducted as part of UPSIDES (Using Peer Support in Developing Empowering Mental Health Services), a 5-year (2018-2022) European Union–funded multinational study that aims to replicate and scale up peer support interventions for people with severe mental illness.[22]

Design

A qualitative research design informed by a critical realist perspective was used. A critical realist approach was chosen as it can help in identifying some of the underlying organisational and

For peer review only - http://bmjopen.bmj.com/site/about/guidelines.xhtml

societal influences of PSW implementation. Focus groups were chosen over other data collection approaches to maximise breadth of data coverage.

Setting

Data were collected from five UPSIDES study sites. Sites were based in two high-income countries (Hamburg and Ulm sites, Germany; Be'er Sheva, Israel) and two low-income countries (Dar es Salaam, Tanzania [low resource setting at the time of data collection, re-banded in 2020 to lower-middle resource setting]; Kampala, Uganda), ensuring regional diversity (Europe, Eastern Mediterranean, sub-Saharan Africa). Sites were classified as low-resource settings because they are based in low-resource countries. As previously reported,[21] sites were also diverse in terms of their experience with peer support work, with two sites (Dar es Salaam, Ulm) having no or very little previous experience.

Participants

Participants were purposively selected to include stakeholders with different perspectives on PSW implementation: actual or potential PSWs, mental health clinicians or managers from hospitals or community services; and regional or national policy-makers. To be included, participants had to be over 18 years of age and capable of providing informed consent.

Participants came from a range of community-based, outpatient and inpatient mental health services in Germany, Uganda and Tanzania, and from a range of community mental health rehabilitation services in Israel. In all sites, multidisciplinary in-patient and out-patient care involves psychotherapy, psychosocial rehabilitation and psychiatric clinics, with some sites also offering family intervention, vocational skills training, cognitive enhancement therapy, psychoeducation, pre-discharge social interventions and physical health care.

Procedures

A conceptual framework – a network of interlinked concepts together providing a comprehensive understanding of a phenomenon[23] – was developed to capture the key elements and implementation influences on the PSW role. The conceptual framework comprised (a) PSW principles and (b) societal and organisational implementation influences. The PSW principles were derived from a researcher-led integration of established core principles from high-resource settings.[13,14,24] At the time of development (2017) there was an absence of integrated evidence, so a systematic review was subsequently undertaken[21], but for the current study the implementation influences were developed through consultation with experts in the UPSIDES consortium.

The conceptual framework informed the development of a topic guide (Supplement 1), comprising open-ended conversational prompts to explore the cultural applicability of PSW principles and to identify societal and organisational implementation influences. Exploration of areas of disagreement was encouraged, as was speculation about potential implementation influences in sites with no experience of PSW. The topic guide was developed in English, commented on by all sites, and then finalised and translated into Kiswahili (Dar es Salaam), German (Hamburg/Ulm), Hebrew (Be'er Sheva) and Luganda (Kampala).

Focus groups were conducted at each site between September and December 2018. In each site, potential participants were identified by mental health clinicians and UPSIDES research workers. Two focus groups were conducted per site, apart from Kampala where four focus groups and three individual interviews were conducted. All focus groups were conducted in the local language and held in a health service or community venue.

For peer review only - http://bmjopen.bmj.com/site/about/guidelines.xhtml

Each focus group comprised five to nine participants, and lasted up to 60 minutes. Facilitators were UPSIDES research workers from the site, who were bilingual in the local language and English, and came from psychology, sociology, health sciences, social work and nursing backgrounds. All facilitators were experienced in qualitative data collection, and actively managed group dynamics to ensure full participation from all participants. Focus groups were recorded using an audio recorder and researchers took field notes during the discussions. After the focus groups, local language transcripts were made, with pseudonymisation of identifying information about participants and third parties. Each local language transcript was translated into English by the local UPSIDES researcher, and checked by the UPSIDES translation leads (Nottingham, UK and Pune, India) for data integrity, identifying points for site checking if needed. Finalised transcripts were password protected and uploaded to a restricted area on the UPSIDES website.

Ethics approval

Ethical approval was obtained by each site: Ulm University Ethics Commission (Application nr. 195/18), Mengo IRB Uganda (MH: 360; MH/REC/141/8/2018), National Institute for Medical Research Tanzania (NIMR/HQ/R.8a/Vol.IX/2982), Institutional Review Board, Ifakara Health Institute, Tanzania (IHI/IRB/No. 28 – 2018), Ärztekammer Hamburg, Germany (MC-230/18), Indian Council of Medical Research (Indo-foreign/66/M/2017-NCD-1), Indian Law Society (ILS/37/2018), and Human Subjects Research Committee of Ben-Gurion University (ref: 1621-2). Each potential participant was given an information sheet in the local language, and the opportunity to ask questions. All participants gave written informed consent before participation.

Patient and public involvement

Individuals with lived experience are involved at multiple levels of the UPSIDES Study, including as part of the site team, as advisory board members, as peer support workers and as authors on some papers.

Analysis

A combination of deductive and Inductive thematic analysis was conducted.[25] The two primary coders were UPSIDES research workers in Dar es Salaam (MR: background in public health) and Nottingham (AC: mental health nursing, sociology). MR and AC independently read all transcripts to familiarise themselves with the content, and start the process of creating preliminary codes and categories. Coding was then discussed with site leads in Dar es Salaam (DS: social science) and Nottingham (MS: clinical psychology), following which a preliminary coding framework for implementation influences was developed. The codebook was then transferred into NVivo 12 software for coding. MR and AC independently coded the same four transcripts, and then discussed and reviewed any differences or discrepancies and any additional themes that emerged from the data. Following review, refinement, and defining of themes, an agreement was reached and new codes were incorporated into the final coding framework. The remaining transcripts were then coded with repeated discussion between coders. The finalised coding framework was iteratively discussed amongst the four primary analysts (AC, MR, MS, and DS) and the wider author team until a consensus was reached.

For peer review only - http://bmjopen.bmj.com/site/about/guidelines.xhtml

Results

A total of 86 individuals participated, as shown in Table 1.

Insert Table 1 here

Theme 1: Community and staff attitudes

Community and staff attitudes toward mental illness were perceived to be both a barrier and also a facilitator for PSWs to perform their roles. Participants reported that people with mental health conditions are considered inferior and rejected, thus making it difficult for the PSWs to perform their roles effectively.

> *When it comes to class, mental health patients are considered second hand, third hand or fourth hand citizens. So we are marginalised among the marginalised. We take the lowest rank status point in the community.* [#9, Kampala, PSW]

Sometimes the PSWs are rejected when they go to visit service users as some family members do not want the mental health status of their relative to be revealed.

> *We are rejected, you can go to that person's place who may not wish to see you and they don't welcome you and you can't insist. Sometimes they just avoid you.* [#12, Kampala, PSW]

Religious beliefs can also act as a barrier in implementing peer support work.

> *There are so many religious leaders who believe that God doesn't fail. They interfere with our work. They stop our patients from taking medicine and they say that God is going to perform miracles then in the end they relapse. The traditional healers believe that mental illness is caused by traditional issues and they don't need Western medicine, they need herbs.* [#26, Kampala, PSW]

PSWs are reported to be role models in the community and have inspired hope to others. The notion of knowledge from experience adds value to the potential contribution of the PSW and helps transform and enhance the value of lived experience.

> *Now that we are role-models in the community people inquire from us about the things which I did to enable me to stabilize while at first they were stigmatizing me. They were beating me but now it is in their families and they are having issues worse than mine. They are like 'you see that mentally sick lady who was here? She is now stable. Let us go and inquire from her so that she can help us'…So we have become brokers in the village.* [#14, Kampala, PSW]

PSWs are an asset to both health service providers and the recipients of peer support services.

For peer review only - http://bmjopen.bmj.com/site/about/guidelines.xhtml

> *Actually it has bridged a gap between service users and service providers. There is some kind of mutual understanding that we have built up. We are treated like staff.* [#14, Kampala, PSW]

Theme 2: Resource availability

Resource availability was an implementation barrier. Participants from in lower resource settings described limited resources to enable PSWs to effectively implement their roles. Peer support workers reported to receive limited remuneration which do not cater for their daily needs.

> *Sometimes the money we get is not enough to cater for these costs such as airtime and transport, you also need money to cater for yourself when you are in the field but sometimes it is little.* [#14, Kampala, PSW]

> *According to the inflation in the country the money can't be enough to move to a community. Sometimes you need to buy airtime to call a peer, you have to fix something or food in the community, you have to get something to drink or eat in the community. Sometimes you find that this peer you are visiting is far away from where you stay, so the money we are paid is not enough.* [#10, Kampala, PSW]

Participants from higher resource settings also reported that, whilst PSWs are an important component of mental health services, there is a limited budget set for them and there is a particular challenge in relation to funding arrangements for the peer support program.

> *The current policy needs to change. The policy says peer specialists have to be everywhere…and it requires resources which we don't have. I am not sure if I can raise the issue but we don't have budgets like the welfare…We need to get a special budget for the program.* [#6, Be'er Sheva, Mental health clinician]

> *Then there is always the question of who finances it. For example, the peer support workers on the ground floor (acute ward), are they financed by the ward budget or hospital budget or are they somehow cross-financed by other projects?* [#3, Hamburg, Mental health clinician]

The facilities available to enable PSWs to perform their roles can be inadequate. It was noted that the working environment in lower-resource health facilities is very poor, exposing the PSWs, mental health workers and services users to many risks.

> *The…Outpatient department can only accommodate four people while there are almost a hundred or ninety people per day so you find that people are just standing.* [#1, Dar es Salaam, Mental health clinician]

> *We don't have enough facilities within the hospital, nurses face some challenges also. When you go to [name] ward, some peers are sleeping down and even divide blankets. You may find that one blanket is divided among 2 to 4 patients.* [#13, Kampala, PSW]

For peer review only - http://bmjopen.bmj.com/site/about/guidelines.xhtml

Theme 3: Organisational culture
Participants reported that working inside structured and hierarchal systems can create a feeling of indebtedness to the organisation which can impact on PSWs autonomy in decision-making and contribute towards feelings of disempowerment.

> *When you enter a job as a consumer provider, at least in the beginning, there is part of you that feels like they are doing you a favour that they hired you. That you have to do what the organisation tells you to do in order to gain experience, etc.* [#8, Be'er Sheva, PSW]

Participants highlighted that the formalisation of the PSW role in such defined systems raises the question of how much of the role should remain informal versus formal in order for PSWs to fit in.

> *I also think that what especially happens with peer specialists, is some sort of formalization of this thing, and how much do we really actually want to formalize it. And how much of it do we have to keep informal, which is one of the worries or dilemmas. The ideals that are really inside of this system that is so formal, and is hierarchical and clear.* [#3, Be'er Sheva, PSW]

Participants reported that the support can be very limited due to the lack of psychological and social support resources available.

In summary, there is diagnosis, treatment mostly pharmacological using medical treatment. [We] do our best to try to provide psychological and social support but those are very limited most of the time. [#30, Kampala, Manager]

Theme 4: Role definition
Having a clear role definition and expectations were reported as important, because without this reference point it can lead to potential role confusion and uncertainty.

> *I don't think it's that easy. They often don't know what they can do themselves. That they also have ideas, what can I actually do now? And I don't think there were enough guidelines or terms of reference.* [#11, Ulm, Mental health clinician]

In addition, the wide variety of tasks PSWs can perform means it can be difficult to construct a role description that accurately fits with real-life peer support practice.

> *The task fields of peer support workers are totally different. That is always person-dependent. We tried to create a kind of job description already and that was very, very difficult. Because we didn't want to restrict the peer support workers too much. Since the tasks always depend on the personality.* [#2, Ulm, Mental health clinician]

Theme 5: Training and Support
Participants described that peer support service is something which is new to other mental health professionals, some of them lack knowledge on what peer support work is. Training health care workers could help in reducing uncertainties among professionals in high and lower resource settings.

For peer review only - http://bmjopen.bmj.com/site/about/guidelines.xhtml

Peer support work is something new in other countries, it needs to be introduced to other staff members in a larger scale. There is also a simple lack of knowledge, not just supervision, but knowledge of what it is all about, and this lack of knowledge also leads to uncertainty among professionals. What, how do we deal with it now, what do we trust them to do, what do we take away from them because it is not the right thing to do? [#11, ULM, Mental health clinician]

The availability of training enables PSWs to know what is expected from them and also understand their needs.

> *The first thing is that they are supposed to receive is training. If they receive training they will know their job description and the techniques of going to the families because there are families which don't want people to know that they have a mental patient.* [#3, Dar es Salaam, Mental health clinician]

> *I can say that the PSW program that I was part of had PSWs who first of all received training especially to understand their needs, making sure they are dealing with mental illness of others and also how they work with PSWs.* [#30, Kampala, Manager]

Participants described how PSWs need support to maintain their wellbeing and carry out their roles. Guidance and supervision from health care workers is very important for PSWs.

> *They shouldn't always work in isolation; they should be supervised.* [#24, Kampala, Mental health clinician]

> *Peer support workers can't be independent, they need professional community nurses to guide them so they can go out in the field, they can be together.* [#30, Kampala, Manager]

> *Coming from a person who is currently dealing with very active symptoms with varying levels of force, a person needs…regulatory capacity, the ability to manage workloads, the ability to receive help, to be helped and to defend oneself.* [#1, Be'er Sheva, Mental health clinician]

Participants identified the need for initial and further training in boundaries, code of conduct, and levels of disclosure.

> *Peer support workers need more training, continuous training. Even if the training is a one-off. So this should be happening. It shouldn't be a big deal.* [#30, Kampala, Manager]

Theme 6: Peer support network

Participants described that having peer support workers access to a peer network enables them to address their potential challenges, made them to feel connected through sharing their experiences and also enabled them to feel stronger together.

> *I can see how a group of peers impact each other non-stop and advances processes almost as if it is a race, but not in a bad way. That is to say, not from a place where you feel that they are forcing you to run, but from a place where a lot of people who are together all the time, are shattering stigmas about one another, I think that a group is stronger.* [#3, Be'er Sheva, PSW]

For peer review only - http://bmjopen.bmj.com/site/about/guidelines.xhtml

Through these networks PSWs get to know each other well, and can identify if another PSW was facing a problem, such networks enabled PSWs to improve their well-being.

> *We peers have what they call buddies. A buddy is a person who knows more about you whereby in case you show signs of relapse that buddy will say '(name) is getting a relapse, (name) do this and this'. He will help you and bring you medical personnel and overcome the situation.* [#11, Kampala, PSW]

Discussion

Our study identified six influences on PSW implementation. At the societal level, community stigma and lay beliefs about mental conditions were influential. At the organisational level, the inter-linked themes of resource allocation and organisational culture were identified, as well as staff attitudes and the challenge of role clarity. At the PSW level, both adequate training /support and a strong peer support network were facilitators of implementation.

The strengths of the study include the sample size, the use of multiple informants, conducting local language topic guides to avoid excluding non-English speaking participants, and the multi-national sampling frame. Credibility of the findings was enhanced by independent coding and the use of multiple analysts. Several limitations can be identified. One significant shortcoming is that sociodemographic characteristics of participants were not collected in a standardised way across all sites, limiting the transferability of findings. Whilst the sample is large for a qualitative study, the findings are complex and nuanced, so our analysis focused on semantic rather than latent coding[25]. Future analysis might explore the relationship between the identified implementation influences, such as how community attitudes may distally impact on resource allocation. Whilst the use of analysts with different professional backgrounds reduced researcher influence on findings, the credibility of the findings could be enhanced by member checking, and including people with lived experience as co-analysts.[26] Finally, the relatively small number of policy-maker participants may account for the limited mention of national and regional policy as an influence.

Two aspects of our findings are noteworthy in relation to other studies. First, the conceptual framework was developed on the basis of research almost exclusively from high-income countries, and identified PSW and organisational influences. The implementation influences identified by participants had a stronger emphasis on societal aspects, including attitudes and role assumptions. Our findings are consistent with the previously-discussed systematic review,[21] published since the conceptual framework was developed. This validates the importance of considering organisational and specifically societal aspects when implementing PSW in different resource settings. This involves developing community awareness regarding the value of peer support, to gain the support of family and community members.[27] Second, the PSW-level influences indicate the need to modify how PSW is provided in different settings. A systematic review of 39 studies, only one from a lower income setting, identified seven types of modification to the PSW role,[28] including recruitment processes, role expectations, training and support. Recent research is expanding to also consider staff attitudes,[29] organisational integration of PSWs,[30,31] organisational climate[32] and context.[33,34]

The primary implication is that more attention needs to be paid to societal attitudes and organisational culture in developing and implementing PSW programmes. Discrimination and stigma relating to mental health are global challenges,[4] but our findings suggest that there is a relationship between community attitudes and the ability to involve people with lived experience in the mental health workforce as PSWs. In terms of organisational culture, the findings reinforce

For peer review only - http://bmjopen.bmj.com/site/about/guidelines.xhtml

existing evidence[35] that organisational culture impacts on recovery support, so organisational transformation may be needed.[36] Approaches to supporting culture change within mental health services include the introduction of pro-recovery interventions,[37,38] development of adjunctive services such as Recovery Colleges,[39] working with teams[40] and introducing co-production[41] and growth-oriented approaches.[42]

Conclusions

A better understanding of the relationship between the identified influences such as societal stigma about mental health and resource allocation decisions is needed. In UPSIDES, the theory of change technique is being used to map out different steps in the implementation of the PSW intervention, and to articulate the connections between these steps.

Acknowledgements

The Using Peer Support In Developing Empowering Mental Health Services (UPSIDES) Study is a multicentre collaboration between the Department for Psychiatry and Psychotherapy II at Ulm University, Germany (Bernd Puschner, coordinator); the Institute of Mental Health at University of Nottingham, UK (Mike Slade); the Department of Psychiatry at University Hospital Hamburg-Eppendorf, Germany (Candelaria Mahlke); Butabika National Referral Hospital, Uganda (David Basangwa); the Centre for Global Mental Health at London School of Hygiene and Tropical Medicine, UK (Grace Ryan); the Department of Health Systems, Impact Evaluation and Policy, Ifakara Health Institute, Dar es Salaam, Tanzania (Donat Shamba); the Department of Social Work at Ben Gurion University of the Negev, Be'er Sheva, Israel (Galia Moran); and the Centre for Mental Health Law and Policy, Pune, India (Jasmine Kalha).

Contributors statement

MR, AC contributed to data collection, analysis and interpretation, and drafting the work. AG, RH contributed to interpretation. JK, CM, RM, GR, DS and MS contributed to the design of the work and data collection, acquisition, analysis and interpretation, and critically revising the work for important intellectual content. DS and MS contributed to the design of the work and data collection, acquisition, analysis and interpretation, and drafting the work. All authors gave final approval of the version to be published, and are accountable for all aspects of the work.

Funding statement

This work was supported by the European Union Horizon 2020 Research and Innovation Programme under Grant Agreement No 779263. MS acknowledges the support of the Center for Mental Health and Substance Abuse, University of South-Eastern Norway and the NIHR Nottingham Biomedical Research Centre. This publication reflects only the authors' views. The Commission is not responsible for any use that may be made of the information it contains. The funding body had no role in the design of the study and in writing the manuscript.

Competing Interests

None declared.

Data sharing statement

Data are available upon reasonable request.

For peer review only

For peer review only - http://bmjopen.bmj.com/site/about/guidelines.xhtml

References

1. Samele C, Frew, S., Urquia, N. European profile of prevention and promotion of mental health (EuroPoPP-MH): EU Health Programme [(EAHC/2010Health/04)]; 2013.
2. Pathare S, Brazinova A, Levav I. Care gap: a comprehensive measure to quantify unmet needs in mental health. *Epidemiology and Psychiatric Sciences* 2018; **27**: 463-7.
3. Collins PY, Patel V, Joestl SS, March D, Insel TR, Daar AS. Grand challenges in global mental health. *Nature* 2011; **475**(7354): 27-30.
4. Patel V, Saxena S, Lund C, et al. The Lancet Commission on global mental health and sustainable development. *Lancet* 2018; **392**: 1553-98.
5. Misra S, Stevenson A, Haroz E, de Menil V, Koenen K. 'Global mental health': systematic review of the term and its implicit priorities. *Br J Psychiatry* 2019; **5**: e47.
6. Davidson L, Bellamy C, Guy K, Miller R. Peer support among persons with severe mental illnesses: a review of evidence and experience. *World Psychiatry* 2012; **11**: 123-8.
7. Mead S. Intentional Peer Support: an alternative approach. Plainfield, NH: Shery Mead Consulting; 2005.
8. Pitt V, Lowe D, Hill S, et al. Consumer-providers of care for adult clients of statutory mental health services. *Cochrane Database of Systematic Reviews* 2013; **3**: CD004807.
9. Lloyd-Evans B, Mayo-Wilson E, Harrison B, et al. A systematic review and meta-analysis of randomised controlled trials of peer support for people with severe mental illness. *BMC Psychiatry* 2014; **14**: 39.
10. Shalaby R, Agyapong V. Peer Support in Mental Health: Literature Review. *JMIR Mental Health* 2020; **7**: e15572.
11. Chinman M, George, P., Dougherty, R., Daniels, A., Ghose, S., Swift, A., Delphin-Rittmon, M. Peer Support Services for Individuals With Serious Mental Illnesses: Assessing the Evidence. *Psychiatr Serv* 2014; **65**: 429-41.
12. White S, Foster R, Marks J, et al. The effectiveness of one-to-one peer support in mental health services: a systematic review and meta-analysis. *BMC Psychiatry* 2020; **20**: 534.
13. Repper J. ImROC Briefing Paper 5. Peer Support Workers: Theory and Practice. London: Centre for Mental Health; 2013.
14. Mead S, Hilton, D., Curtis, L. Peer support: A theoretical perspective. *Psychiatric Rehabilitation Journal* 2001; **25**: 134–41.
15. Fan Y, Ma N, Ma L, Xu W, Lamberti JS, Caine E. A community-based peer support service for persons with severe mental illness in China. *BMC Psychiatry* 2018; **18**: 170.
16. Pathare S, Kalha J, Krishnamoorthy S. Peer support for mental illness in India: An underutilised resource. *Epidemiology and Psychiatric Sciences* 2018; **27**: 415-9.
17. Moran GS. The mental health consumer movement and peer providers in Israel. *Epidemiology and Psychiatric Sciences* 2018; **27**: 420-6.
18. Lee YY, Ang S, Chua HC, Subramaniam M. Peer Support in Mental Health: A Growing Movement in Singapore. *Annals of Academy of Medicine Singapore* 2019; **48**: 95-7.
19. Ryan G, Kamuhiirwa M, Mugisha J, et al. Peer support for frequent users of inpatient mental health care in Uganda: protocol of a quasi-experimental study. *BMC Psychiatry* 2019; **19**: 374.
20. Piat M, Sofouli E, Sabetti J, et al. Protocol for a mixed studies systematic review on the implementation of the recovery approach in adult mental health services. *BMJ Open* 2017; **7**(8).
21. Ibrahim N, Thompson D, Nixdorf R, et al. A systematic review of influences on implementation of peer support work for adults with mental health problems. *Soc Psychiatry Psychiatr Epidemiol* 2020; **55**: 285-93.
22. Puschner B, Repper J, Mahlke C, et al. Using Peer Support in Developing Empowering Mental Health Services (UPSIDES): Background, rationale and methodology. *Annals of Global Health* 2019; **85**(53): 1-10.

For peer review only - http://bmjopen.bmj.com/site/about/guidelines.xhtml

23. Jabareen Y. Building a Conceptual Framework: Philosophy, Definitions, and Procedure. *International Journal of Qualitative Methods* 2009; **8**: 49-62.
24. Repper J, Perkins R. ImROC Briefing Paper 6. The Team Recovery Implementation Plan: a framework for creating recovery-focused services. London: Centre for Mental Health; 2013.
25. Braun V, Clarke V, Hayfield N, Terry G. Thematic analysis. In: Laiamputtong L, ed. Handbook of Research Methods in Social Sciences. Singapore: Springer; 2019: 843-60.
26. Jennings H, Slade M, Bates P, Munday E, Toney R. Best practice framework for Patient and Public Involvement (PPI) in collaborative data analysis of qualitative mental health research: methodology development and refinement. *BMC Psychiatry* 2018; **18**: 213.
27. Pearson H, Adams B, Eaton J, Illingworth M. Community Mental Health Good Practice Guide: Peer Support: CBM Global Disability Inclusion; 2021.
28. Charles A, Thompson D, Nixdorf R, et al. A typology of modifications to peer support work for adults with mental health problems: systematic review. *Br J Psychiatry* 2020; **216**: 301-7.
29. Moore T, Zeeman L. More 'milk' than 'psychology or tablets': Mental health professionals' perspectives on the value of peer support workers. *Health Expectations* DOI: 10.1111/hex.13151.
30. Charles A, Nixdorf R, Ibrahim N, et al. Initial training for mental health peer support workers: international Delphi Consultation. *JMIR Mental Health* 2021; **5**: e25528.
31. Otte I, Werning A, Nossek A, Vollmann J, Juckel G, Gather J. Challenges faced by peer support workers during the integration into hospital-based mental health-care teams: Results from a qualitative interview study. *Int J Soc Psychiatry* 2020; **66**: 263-9.
32. Jones N, Teague G, Wolf J, Rosen C. Organizational Climate and Support Among Peer Specialists Working in Peer-Run, Hybrid and Conventional Mental Health Settings. *Administration and Policy in Mental Health and Mental Health Services Research* 2020; **47**: 150-67.
33. Adams W. Unintended consequences of institutionalizing peer support work in mental healthcare. *Soc Sci Med* 2020; **262**: https://doi.org/10.1016/j.socscimed.2020.113249.
34. Urichuk L, Hrabok M, Hay K, et al. Enhancing peer support experience for patients discharged from acute psychiatric care: protocol for a randomised controlled pilot trial. *BMJ Open* 2018; **8**: e022433.
35. Clossey L, Rheinheimer D. Exploring the effect of organizational culture on consumer perceptions of agency support for mental health recovery. *Community Ment Health J* 2014; **50**(4): 427-34.
36. Le Boutillier C, Chevalier A, Lawrence V, et al. Staff understanding of recovery-orientated mental health practice: a systematic review and narrative synthesis. *Implementation Science* 2015; **10**: 87.
37. Meadows G, Brophy L, Shawyer F, et al. REFOCUS-PULSAR recovery-oriented practice training in specialist mental health care: a stepped-wedge cluster randomised controlled trial. *Lancet Psychiatry* 2019; **6**: 103-14.
38. Slade M, Bird V, Clarke E, et al. Supporting recovery in patients with psychosis through care by community-based adult mental health teams (REFOCUS): a multisite, cluster, randomised, controlled trial. *Lancet Psychiatry* 2015; **2**: 503-14.
39. Crowther A, Taylor A, Toney R, et al. The impact of Recovery Colleges on mental health staff, services and society. *Epidemiology and Psychiatric Sciences* 2019; **28**: 481-8.
40. Vacher G. Utilising Team Recovery Implementation Plan (TRIP): embedding recovery-focused practice in rehabilitation services. *Mental Health and Social Inclusion* 2017; **21**: 240-7.
41. Lewis A, King T, Herbert L, Repper J. Co-Production – Sharing Our Experiences, Reflecting On Our Learning. Nottingham: ImROC; 2017.

For peer review only

For peer review only - http://bmjopen.bmj.com/site/about/guidelines.xhtml

42. Slade M, Rennick-Egglestone S, Blackie L, et al. Post-traumatic growth in mental health recovery: qualitative study of narratives. *BMJ Open* 2019; **9**: e029342.

For peer review only

For peer review only - http://bmjopen.bmj.com/site/about/guidelines.xhtml

Table 1: Focus group participants (n=86)

Site	Focus groups	n	Participant				Gender	
			Potential or actual PSW	Mental health worker	Mental health manager	Policy-maker	Male	Female
Ulm	2	12	1	10	0	1	4	8
Hamburg	2	12	7	5	0	0	4	8
Kampala	4	32	16	14	1	1	10	22
Dar es Salaam	2	16	0	12	4	0	7	9
Be'er Sheva	2	14	2	8	4	0	5	9
**Total**	**12**	**86**	**26**	**49**	**9**	**2**	**30**	**56**

For peer review only - http://bmjopen.bmj.com/site/about/guidelines.xhtml

Instructions:

This guide is to be used to facilitate focus group discussion with (1.1) health workers who are using or plan to recruit peer support workers. (Psychiatric nurses, clinicians working in mental health ward / departments) and (1.2) local stakeholders with relevant expertise relating to implementation of peer work (including clinicians and managers who currently, previously or in the future may employ peer support workers, and people who currently, previously or in the future may work as peer support workers).

Interviewer ID ___ ___ ___ ___

Interview date (DD/MM/YYYY): __ __ / __ __ / __ __ __

Location of the interview ____________________________________

Country: ____________________________________

Introduction:

Ensure that the participants are comfortable.

Hi and welcome to this session. My name is ___ and my colleague is___. Thank you for taking your time to talk to us about your experiences with people with severe mental illness and peer support workers. We will discuss topics related to the key characteristics of peer support workers and the challenges they may face. The discussion will take around 60minutes. The interview will be recorded using an audio recorder as we will not have to write down all your answers and we will not miss any of your useful comments. Informed consent will be obtained from all the participants and confidentiality will be assured to all the participants. Before we begin, I would like to know if you have any questions.

Explain you are starting the audio recorder.

Time interview started:

Theme 1: Socio-demographic information of the respondents

Collect data on name, age, education, position and number of years working in this position and give a code to each participant.

Explain again to the participant that:

- I want to learn about your experience, thoughts and perspective on this topic of using peer support workers for improving mental health conditions in your country.
- There is no ‘right’ or ‘wrong’ answer.

Say: We are planning to recruit and train peer support workers. Peer providers in mental health are individuals with severe mental illness who are further along in their recovery, who support others with similar conditions by role modelling that recovery is possible, sharing knowledge from experience and using reciprocal empathic relationships. We are thinking of asking these peer support workers to identify and visit individuals with severe mental illness. We would like to get your opinion on how best to implement PSWs intervention in this region. We would like to know key characteristics for PSWs and challenges that could hinder them performing their

For peer review only - http://bmjopen.bmj.com/site/about/guidelines.xhtml

tasks. Please be frank as we want the information to be the right ones for this community.

Theme 2: Social cultural factors and acceptability of PSWs

1. Based on your experiences, how does the community care for people with SMIs? Where do they seek care? Are there other places apart from the hospital? Describe all the places people seek care for SMI.

2. What are your main roles in supporting people with SMIs? What are the main challenges that you are facing in doing your job? What are the solutions to those challenges?

   - Probe to know if mental health programmes are given a priority?
   - Is there a national policy or guideline for mental health conditions? Are these accessible?
   - What has been the main challenge your department has been facing in managing/meeting the needs of people with Severe Mental Illness? Probe for various challenges the department has been facing in regard to SMI programmes? For each challenge mentioned, ask how the department could handle it i.e. any potential solution and/or suggestion on how to address it?

3. With your experience working in this area, could you please tell us what do you know about PSWs and how do they perform their duties?

   - Probe for, how they are recruited, who recruits them, what are they exactly doing, etc?
   - Please, describe any systems for linking people with peers who can serve as role models in this facility or community? (e.g. through contact with local user-run groups).

4. What key qualities would you want a peer support worker to have or NOT to have? We want to find out what sort of person the respondents would trust and value, perform the work well and responsibly.

5. Could you please tell us what are the specific things, you would want a peer support worker to do?

   Probe for specific terminologogies, main activities, duration of activities. care planning

Theme 3: Institution / facility readiness to incorporate PSW

6. In your opinion – for using peer support workers, how could it be implemented / improved at this facility?

For peer review only - http://bmjopen.bmj.com/site/about/guidelines.xhtml

- Probe: training, logistics, management, supervision, monitoring, capacity development, resources etc.

7. What are the facilitators and barriers in providing peer support to people with SMI in your facility or in your region?

- Probe for resources, skilled personnel, infrastructure, systems support?

8. Is there anything more you would like to add about your experiences with, or views on, using peer support workers?

Time interview end:

Thank the participant for his / her time. Remind them that the information will be kept confidential.

Interviewer comments on how the interview went:

For peer review only

Standards for Reporting Qualitative Research Checklist

Item	Topic	Where addressed
**Title and abstract**		
S1	**Title**<br>Concise description of the nature and topic of the study Identifying the study as qualitative or indicating the approach (e.g., ethnography, grounded theory) or data collection methods (e.g., interview, focus group) is recommended	Title
S2	**Abstract**<br>Summary of key elements of the study using the abstract format of the intended publication; typically includes background, purpose, methods, results, and conclusions	Abstract
**Introduction**		
S3	**Problem formulation** Description and significance of the problem/phenomenon studied; review of relevant theory and empirical work; problem statement	Introduction
S4	Purpose or research question Purpose of the study and specific objectives or questions	Introduction final paragraph
**Methods**		
S5	**Qualitative approach and research paradigm**<br>Qualitative approach (e.g., ethnography, grounded theory, case study, phenomenology, narrative research) and guiding theory if appropriate; identifying the research paradigm (e.g., postpositivist, constructivist/interpretivist) is also recommended; rationale	Methods Design<br>Method Procedures paragraph 2<br>Methods Analysis
S6	**Researcher characteristics and reflexivity**<br>Researchers' characteristics that may influence the research, including personal attributes, qualifications/experience, relationship with participants, assumptions, and/or presuppositions; potential or actual interaction between researchers' characteristics and the research questions, approach, methods, results, and/or transferability	Methods Analysis<br>Discussion paragraph 2
S7	**Context**	Methods Setting

For peer review only

	Setting/site and salient contextual factors; rationale	
S8	**Sampling strategy**<br>How and why research participants, documents, or events were selected; criteria for deciding when no further sampling was necessary (e.g., sampling saturation); rationale	Methods Participants<br>Methods Procedures final paragraph<br>Methods Sampling
S9	**Ethical issues pertaining to human subjects** Documentation of approval by an appropriate ethics review board and participant consent, or explanation for lack thereof; other confidentiality and data security issues	Methods Ethics approval
S10	**Data collection methods** Types of data collected; details of data collection procedures including (as appropriate) start and stop dates of data collection and analysis, iterative process, triangulation of sources/methods, and modification of procedures in response to evolving study findings; rationale. The rationale should briefly discuss the justification for choosing that theory, approach, method, or technique rather than other options available, the assumptions and limitations implicit in those choices, and how those choices influence study conclusions and transferability. As appropriate, the rationale for several items might be discussed together.	Methods Procedures<br>Methods Analysis
S11	**Data collection instruments and technologies**<br>Description of instruments (e.g., interview guides, questionnaires) and devices (e.g., audio recorders) used for data collection; if/how the instrument(s) changed over the course of the study	Methods Procedures paragraphs 1 and 2
S12	**Units of study**<br>Number and relevant characteristics of participants, documents, or events included in the study; level of participation (could be reported in results)	Results Table 1
S13	**Data processing**<br>Methods for processing data prior to and during analysis, including transcription, data entry, data management and security, verification of data integrity, data coding, and anonymization/deidentification of excerpts	Methods Procedures final paragraph
S14	**Data analysis**<br>Process by which inferences, themes, etc., were identified and developed, including the researchers involved in data analysis; usually references a specific paradigm or approach; rationale	Methods Analysis

For peer review only

S15	**Techniques to enhance trustworthiness** Techniques to enhance trustworthiness and credibility of data analysis (e.g., member checking, audit trail, triangulation); rationale	Methods Analysis Discussion strengths and limitations paragraph
**Results/findings**		
S16	**Synthesis and interpretation** Main findings (e.g., interpretations, inferences, and themes); might include development of a theory or model, or integration with prior research or theory	Results
S17	**Links to empirical data** Evidence (e.g., quotes, field notes, text excerpts, photographs) to substantiate analytic findings	Results
**Discussion**		
S18	**Integration with prior work, implications, transferability, and contribution(s) to the field** Short summary of main findings; explanation of how findings and conclusions connect to, support, elaborate on, or challenge conclusions of earlier scholarship; discussion of scope of application/generalizability; identification of unique contribution(s) to scholarship in a discipline or field	Discussion
S19	**Limitations** Trustworthiness and limitations of findings	Discussion paragraph 2
**Other**		
S20	**Conflicts of interest** Potential sources of influence or perceived influence on study conduct and conclusions; how these were managed	Competing Interests statement
S21	**Funding** Sources of funding and other support; role of funders in data collection, interpretation, and reporting	Funding statement

For peer review only

BMJ Open

Societal and organisational influences on implementation of mental health peer support work in low-income and high-income settings: qualitative focus group study

Journal:	*BMJ Open*
Manuscript ID	bmjopen-2021-058724.R2
Article Type:	Original research
Date Submitted by the Author:	06-Sep-2022
Complete List of Authors:	Ramesh, Mary; Ifakara Health Institute, Department of Health System, Impact Evaluation and Policy<br>Charles, Ashleigh; University of Nottingham, School of Health Sciences<br>Grayzman, Alina; Ben-Gurion University of the Negev Faculty of Health Sciences, Department of Social Work, Ben Gurion University of the Negev<br>Hiltensperger, Ramona; Department of Psychiatry II<br>Kalha, Jasmine; Indian Law Society<br>Kulkarni, Arti; Indian Law Society<br>Mahlke, Candelaria ; University Medical Cente Hamburg, Candelaria Irene Mahlke<br>Moran, Galia; Ben-Gurion University of the Negev, Department of Social Work<br>Mpango, Richard; Butabika National Referral Hospital; Soroti University<br>Stierlin, Annabel; Ulm University, Institute of Epidemiology and Medical Biometry<br>Nixdorf, Rebecca ; University Medical Centre Hamburg-Eppendorf, Department of Psychiatry<br>Ryan, Grace; London School of Hygiene and Tropical Medicine, Population Health<br>Shamba, Donat ; Ifakara Health Institute, Department of Health Systems, Impact Evaluation and Policy<br>Slade, Mike; University of Nottingham School of Health Sciences
**Primary Subject Heading**:	Global health
Secondary Subject Heading:	Global health, Mental health, Qualitative research
Keywords:	MENTAL HEALTH, QUALITATIVE RESEARCH, PUBLIC HEALTH

SCHOLARONE™
Manuscripts

I, the Submitting Author has the right to grant and does grant on behalf of all authors of the Work (as defined in the below author licence), an exclusive licence and/or a non-exclusive licence for contributions from authors who are: i) UK Crown employees; ii) where BMJ has agreed a CC-BY licence shall apply, and/or iii) in accordance with the terms applicable for US Federal Government officers or employees acting as part of their official duties; on a worldwide, perpetual, irrevocable, royalty-free basis to BMJ Publishing Group Ltd ("BMJ") its licensees and where the relevant Journal is co-owned by BMJ to the co-owners of the Journal, to publish the Work in this journal and any other BMJ products and to exploit all rights, as set out in our licence.

The Submitting Author accepts and understands that any supply made under these terms is made by BMJ to the Submitting Author unless you are acting as an employee on behalf of your employer or a postgraduate student of an affiliated institution which is paying any applicable article publishing charge ("APC") for Open Access articles. Where the Submitting Author wishes to make the Work available on an Open Access basis (and intends to pay the relevant APC), the terms of reuse of such Open Access shall be governed by a Creative Commons licence – details of these licences and which Creative Commons licence will apply to this Work are set out in our licence referred to above.

Other than as permitted in any relevant BMJ Author's Self Archiving Policies, I confirm this Work has not been accepted for publication elsewhere, is not being considered for publication elsewhere and does not duplicate material already published. I confirm all authors consent to publication of this Work and authorise the granting of this licence.

For peer review only

Societal and organisational influences on implementation of mental health peer support work in low-income and high-income settings: qualitative focus group study

Mary Ramesh [1 a]
Ashleigh Charles [2 a]
Alina Grayzman [3]
Ramona Hiltensperger [4]
Jasmine Kalha [5]
Arti Kulkarni[5]
Candelaria Mahlke [6]
Galia Moran[3]
Richard Mpango [7 8 9]
Annabel Sandra Müller-Stierlin [4]
Rebecca Nixdorf [6]
Grace Ryan [10]
Donat Shamba [1 b *]
Mike Slade [2 b]

[1] Department of Health Systems, Impact Evaluation and Policy, Ifakara Health Institute, Dar es Salaam, Tanzania
[2] School of Health Sciences, Institute of Mental Health, University of Nottingham, UK
[3] Department of Social Work, Ben Gurion University of the Negev, Be'er Sheva, Israel
[4] Department of Psychiatry II, Ulm University, Ulm, Germany
[5] Centre for Mental Health Law and Policy, Indian Law Society, Pune, India
[6] Department of Psychiatry, University Medical Center Hamburg-Eppendorf, Germany
[7] Butabika National Referral Hospital, Uganda
[8] School of Health Sciences, Soroti University, Soroti, Uganda
[9] MRC/UVRI and LSHTM Uganda Research Unit, Entebbe, Uganda
[10] Centre for Global Mental Health, London School of Hygiene and Tropical Medicine, UK

[a] Ramesh and Charles contributed equally and are joint first authors
[b] Shamba and Slade contributed equally and are joint last authors

* Correspondence to: Donat Shamba: dshamba@ihi.or.tz

ORCID iDs
MR: 0000-0002-4942-3923; AC: 0000-0003-2222-4358; AG 0000-0002-8192-7697; RH: 0000-0003-2544-4188; JK: 0000-0001-7357-2366; AK: 0000-0002-3281-4388; CM: 0000-0001-9573-6106;GM: 0000-0001-9718-1773; RM: 0000-0001-6960-3174; AMS: 0000-0003-2812-5115; RN: 0000-0002-3064-8380; GR: 0000-0002-9310-3513; DS: 0000-0001-7431-7199; MS: 0000-0001-7020-3434

Word count
4759 words.

For peer review only - http://bmjopen.bmj.com/site/about/guidelines.xhtml

ABSTRACT

Objectives
Despite the established evidence base for mental health peer support work, widespread implementation remains a challenge. This study aimed to explore societal and organisational influences on the implementation of peer support work in low and high income settings.

Design
Study sites conducted two focus groups in local languages at each site, using a topic guide based on a conceptual framework describing eight peer support worker principles and five implementation issues. Transcripts were translated into English and an inductive thematic analysis was conducted to characterise implementation influences.

Setting
The study took place in five secondary mental health care sites as part of the Using Peer Support in Developing Empowering Mental Health Services (UPSIDES) study, comprising three high-income sites (Hamburg and Ulm, Germany; Be'er Sheva, Israel) and two low-income sites (Dar es Salaam, Tanzania; Kampala, Uganda) chosen for diversity both in region and in experience of peer support work.

Participants
86 participants (56 female, 30 male) met inclusion criteria: aged over 18 years; actual or potential peer support worker (n=26) or mental health clinician (n=49) or hospital / community manager or regional / national policy-maker (n=11); able to give informed consent.

Results
Six themes relating to implementation influences were identified: community and staff attitudes; resource availability; organisational culture; role definition; training and support; and peer support network.

Conclusions
This is the first multi-country study to explore societal attitudes and organisational culture influences on the implementation of peer support. Addressing community-level discrimination and developing a recovery orientation in mental health systems can contribute to effective implementation of peer support work. The relationship between societal stigma about mental health and resource allocation decisions warrants future investigation.

Trial registration
UPSIDES RCT: ISRCTN26008944

For peer review only

Strengths and limitations of this study

- The sample size (n=86) and sampling across two low-income and three high-income sites increases the credibility of the findings and their relevance for both low- and high-income settings.
- Independent coding by multiple analysts from different cultures enhances trustworthiness.
- Sociodemographic characteristics were not sufficiently collected to be reported, limiting transferability of findings.
- Study participants were peer support workers and mental health professionals, there is a need to conduct further studies with service users or recipients of peer support services in order to understand their perceptions on the influences for the implementation of peer support work.
- Two FGDs per site may not reach saturation. However, the study involved different sets of respondents to bring the perspectives of different groups who either had an experience in peer support work or were planning to use peer support workers.

For peer review only

Introduction

Many people living with severe mental illness do not receive adequate care. For example, in Europe the EuroPoPP-MH study found that a comprehensive range of community-based services existed in only eight of 29 countries.[1] The resulting gap between demand and supply is called the treatment gap, or care gap.[2] While mental health has been identified as a global priority,[3] the mental health treatment gap remains, and is largest in low-resource settings.[4]

One reason for the treatment gap in low-resource settings is that global mental health initiatives do not sufficiently address contextual aspects, such as up-stream social determinants, geographic and linguistic differences, and sociodemographic influences such as ethnicity, caste and tribe.[5] This leads to barriers in receiving mental health treatment, including stigma, social exclusion and differing availability of resources.

Mental health peer support is an established intervention involving a person with lived experience of mental health problems and recovery employed to offer support to others with mental health problems. Peer support workers (PSWs) act as credible role models of recovery,[6] instilling hope through positive self-disclosure, modelling the use of experiential knowledge for self-care, and offering supportive relationships based on intentional mutuality.[7] There is a strong empirical evidence base for PSWs.[8-11] The most recent systematic review identified 19 randomised controlled trials,[12] all from high income countries. This review found PSW was associated with beneficial outcomes in relation to supporting recovery, empowerment and social networks. However, heterogeneity in the implementation of peer support was identified as an important knowledge gap.

Most research on mental health peer support work has been conducted in high-resource settings, including creation of core PSW principles[13,14] and evaluation.[8] However, PSW roles are increasingly being developed, formalised, and implemented in more diverse settings, such as China,[15] India,[16] Israel,[17] Singapore[18] and Uganda_ENREF_24.[19] An important knowledge gap therefore exists in relation to PSW implementation influences across settings with different resource levels.[20] A recent systematic review synthesised 53 studies to identify 14 influences on implementation of mental health PSW.[21] The most commonly influence was organisational culture, identified in 53% of studies, training, and role definition. Societal influences were also identified, including PSW access to a peer network, resource availability and financial arrangements.

The Grand Challenges for Mental Health initiative identified the importance of research along the translation continuum including implementation, and emphasised that implementation is a challenge not just in low and middle income countries.[3] In other words, the focus should be on implementation research including both lower and higher resource settings. To our knowledge, no study has explored PSW implementation across multiple countries. The aim of this study was to explore and characterise the societal and organisational influences on the implementation of mental health peer support work in lower income and higher income settings.

Methods

This study was conducted as part of UPSIDES (Using Peer Support in Developing Empowering Mental Health Services), a 5-year (2018-2022) European Union–funded multinational study that aims to replicate and scale up peer support interventions for people with severe mental illness.[22]

Design

A qualitative research design informed by a critical realist perspective was used. A critical realist approach was chosen as it can help in identifying some of the underlying organisational and

For peer review only - http://bmjopen.bmj.com/site/about/guidelines.xhtml

societal influences of PSW implementation. Focus groups were chosen over other data collection approaches to maximise breadth of data coverage.

Setting

Data were collected from five UPSIDES study sites. Sites were based in two high-income countries (Hamburg and Ulm sites, Germany; Be'er Sheva, Israel) and two low-income countries (Dar es Salaam, Tanzania [low resource setting at the time of data collection, re-banded in 2020 to lower-middle resource setting]; Kampala, Uganda), ensuring regional diversity (Europe, Eastern Mediterranean, sub-Saharan Africa). Sites were classified as low-resource settings because they are based in low-resource countries. As previously reported,[21] sites were also diverse in terms of their experience with peer support work, with two sites (Dar es Salaam, Ulm) having no or very little previous experience.

Participants

Participants were purposively selected to include stakeholders with different perspectives on PSW implementation: actual or potential PSWs, mental health clinicians or managers from hospitals or community services; and regional or national policy-makers. To be included, participants had to be over 18 years of age and capable of providing informed consent.

Participants came from a range of community-based, outpatient and inpatient mental health services in Germany, Uganda and Tanzania, and from a range of community mental health rehabilitation services in Israel. In all sites, multidisciplinary in-patient and out-patient care involves psychotherapy, psychosocial rehabilitation and psychiatric clinics, with some sites also offering family intervention, vocational skills training, cognitive enhancement therapy, psychoeducation, pre-discharge social interventions and physical health care.

Procedures

A conceptual framework – a network of interlinked concepts together providing a comprehensive understanding of a phenomenon[23] – was developed to capture the key elements and implementation influences on the PSW role. The conceptual framework comprised (a) PSW principles and (b) societal and organisational implementation influences, as shown in table 1. The PSW principles were derived from a researcher-led integration of established core principles from high-resource settings.[13,14,24] At the time of development (2017) there was an absence of integrated evidence, so a systematic review was subsequently undertaken[21], but for the current study the implementation influences were developed through consultation with experts in the UPSIDES consortium.

The conceptual framework informed the development of a topic guide (Supplement 1), comprising open-ended conversational prompts to explore the cultural applicability of PSW principles and to identify societal and organisational implementation influences. Exploration of areas of disagreement was encouraged, as was speculation about potential implementation influences in sites with no experience of PSW. The topic guide was developed in English, commented on by all sites, and then finalised and translated into Kiswahili (Dar es Salaam), German (Hamburg/Ulm), Hebrew (Be'er Sheva) and Luganda (Kampala).

Insert table 1 here

Focus groups were conducted at each site between September and December 2018. In each site, potential participants were identified by mental health clinicians and UPSIDES research workers. Two focus groups were conducted per site, apart from Kampala where four focus groups (two for PSWs, two for other stakeholders) and three individual interviews were conducted. All

For peer review only - http://bmjopen.bmj.com/site/about/guidelines.xhtml

focus groups were conducted in the local language and held in a health service or community venue.

Each focus group comprised five to nine participants, and lasted up to 60 minutes. Facilitators were UPSIDES research workers from the site, who were bilingual in the local language and English, and came from psychology, sociology, health sciences, social work and nursing backgrounds. All facilitators were experienced in qualitative data collection, and actively managed group dynamics to ensure full participation from all participants. Focus groups were recorded using an audio recorder and researchers took field notes during the discussions. After the focus groups, local language transcripts were made, with pseudonymisation of identifying information about participants and third parties. Each local language transcript was translated into English by the local UPSIDES researcher, and checked by the UPSIDES translation leads (Nottingham, UK and Pune, India) for data integrity, identifying points for site checking if needed. Finalised transcripts were password protected and uploaded to a restricted area on the UPSIDES website.

Ethics approval

Ethical approval was obtained by each site: Ulm University Ethics Commission (Application nr. 195/18), Mengo IRB Uganda (MH: 360; MH/REC/141/8/2018), National Institute for Medical Research Tanzania (NIMR/HQ/R.8a/Vol.IX/2982), Institutional Review Board, Ifakara Health Institute, Tanzania (IHI/IRB/No. 28 – 2018), Ärztekammer Hamburg, Germany (MC-230/18), Indian Council of Medical Research (Indo-foreign/66/M/2017-NCD-1), Indian Law Society (ILS/37/2018), and Human Subjects Research Committee of Ben-Gurion University (ref: 1621-2). Each potential participant was given an information sheet in the local language, and the opportunity to ask questions. All participants gave written informed consent before participation.

Patient and public involvement

Individuals with lived experience are involved at multiple levels of the UPSIDES Study, including as part of the site team, as advisory board members, as peer support workers and as authors on some papers. No specific patient and public involvement was used in the current study.

Analysis

A combination of deductive and Inductive thematic analysis was conducted.[25] The two primary coders were UPSIDES research workers in Dar es Salaam (MR: background in public health) and Nottingham (AC: mental health nursing, sociology). MR and AC independently read all transcripts to familiarise themselves with the content, and start the process of creating preliminary codes and categories. Coding was then discussed with site leads in Dar es Salaam (DS: social science) and Nottingham (MS: clinical psychology), following which a preliminary coding framework for implementation influences was developed. The codebook was then transferred into NVivo 12 software for coding. MR and AC independently coded the same four transcripts, and then discussed and reviewed any differences or discrepancies and any additional themes that emerged from the data. Following review, refinement, and defining of themes, an agreement was reached and new codes were incorporated into the final coding framework. The remaining transcripts were then coded with repeated discussion between coders. The finalised coding framework was iteratively discussed amongst the four primary analysts (AC, MR, MS, and DS) and the wider author team until a consensus was reached.

For peer review only - http://bmjopen.bmj.com/site/about/guidelines.xhtml

Results

A total of 86 individuals participated, as shown in Table 2.

Insert Table 2 here

Six implementation influence themes were identified: community and staff attitudes; resource availability; organisational culture; role definition; training and support; and peer support network, as shown in figure 1.

Insert figure 1 here

Theme 1: Community and staff attitudes

Community and staff attitudes toward mental illness were perceived to be both a barrier and also a facilitator for PSWs to perform their roles. Some participants especially in lower income countries, reported that people with mental health conditions are considered inferior and are also rejected, thus making it difficult for the PSWs to perform their roles effectively.

> *When it comes to class, mental health patients are considered second hand, third hand or fourth hand citizens. So we are marginalised among the marginalised. We take the lowest rank status point in the community.* [#9, Kampala, PSW]

Mainly in lower income countries, the PSWs are rejected when they go to visit service users as some family members do not want the mental health status of their relative to be revealed, thus making it difficult for the PSWs to perform their roles.

> *We are rejected, you can go to that person's place who may not wish to see you and they don't welcome you and you can't insist. Sometimes they just avoid you.* [#12, Kampala, PSW]

Furthermore, religious beliefs can also act as a barrier in implementing peer support work in lower income countries.

> *There are so many religious leaders who believe that God doesn't fail. They interfere with our work. They stop our patients from taking medicine and they say that God is going to perform miracles then in the end they relapse. The traditional healers believe that mental illness is caused by traditional issues and they don't need Western medicine, they need herbs.* [#26, Kampala, PSW]

In both lower and higher income countries, peer support workers face stigma from the health service providers. Peer support workers are labelled and considered to be mad and in some cases health workers raise questions as to why a person with a mental illness is part of the staff as described by peer support workers below.

> *Stigma prevails mainly among doctors and employees in medical and rehabilitation services. I blame it on the illness model as perceived by most. The model holds that illness is an inherent state, a permanent life solution. In my opinion, this is the core of the problem.*

For peer review only - http://bmjopen.bmj.com/site/about/guidelines.xhtml

Even if you have stopped medication and have been well for ten years, still the label remains. It sounds like it's for life. We should stop labelling. [#4, Be'er Sheva, Peer Support Worker]

And even here at [name] hospital some of the professionals say 'who can work with those mad ones?'. But some of them, those who accepted us are happy to work with us, they even smile at us and talk to us but there are others who think that mental illness is contagious. [#5, Kampala, PSW]

Acceptance of the PSWs by the health service providers play a significant role in facilitating the implementation of peer support services. PSWs lack of acceptance from health service providers can cause them to be unstable and fail to fulfil their duties as described by a participant from higher income settings.

Not being accepted made the PSW to be alone. You find that a PSW is stable in the beginning but you notice that she was destabilized in the course of being a peer support workers due to the pressure from outside and lack of acceptance from the team. [#009, ULM, Mental health clinician]

Positive community initiatives facilitate the implementation of peer support services especially in lower income countries.. These initiatives have enabled the PSWs to be known as role models in the community and have inspired hope to others. Additionally, the notion of knowledge from experience adds value to the potential contribution of the PSW and helps transform and enhance the value of lived experience.

Now that we are role-models in the community people inquire from us about the things which I did to enable me to stabilize while at first they were stigmatizing me. They were beating me but now it is in their families and they are having issues worse than mine. They are like 'you see that mentally sick lady who was here? She is now stable. Let us go and inquire from her so that she can help us'…So we have become brokers in the village. [#14, Kampala, PSW]

Despite the fact that there are some health providers who label peer support workers, there are also others who think that peer support workers are an asset to both health service providers and the recipients of peer support services. One participant from a lower income country perceived that peer support workers act as a bridge between the mental health workers and the service users. PSWs and their peers share a mutual relationship, peers open up more to the PSWs than mental health staff.

Actually it has bridged a gap between service users and service providers. There is some kind of mutual understanding that we have built up. We are treated like staff. [#14, Kampala, PSW]

Theme 2: Resource availability

Providing resources to Peer Support Workers is an influencing factor in the provision of peer support services. Some participants from our study have reported that PSW have limited resources in terms of money for airtime, transport, little payment which do not cater for their daily needs especially in lower income settings.

For peer review only - http://bmjopen.bmj.com/site/about/guidelines.xhtml

Sometimes the money we get is not enough to cater for these costs such as airtime and transport, you also need money to cater for yourself when you are in the field but sometimes it is little. [#14, Kampala, PSW]

According to the inflation in the country the money can't be enough to move to a community. Sometimes you need to buy airtime to call a peer, you have to fix something or food in the community, you have to get something to drink or eat in the community. Sometimes you find that this peer you are visiting is far away from where you stay, so the money we are paid is not enough. [#10, Kampala, PSW]

Participants from higher income countries also reported that, whilst PSWs are an important component of mental health services, there is a limited budget set for them and there is a particular challenge in relation to funding arrangements for the peer support program.

The current policy needs to change. The policy says peer specialists have to be everywhere…and it requires resources which we don't have. I am not sure if I can raise the issue but we don't have budgets like the welfare…We need to get a special budget for the program. [#6, Be'er Sheva, Mental health clinician]

Then there is always the question of who finances it. For example, the peer support workers on the ground floor (acute ward), are they financed by the ward budget or hospital budget or are they somehow cross-financed by other projects? [#3, Hamburg, Mental health clinician]

The facilities available to enable PSWs to perform their roles can be inadequate. It was noted that the working environment in lower-resource health facilities is very poor, exposing the PSWs, mental health workers and services users to many risks.

The…Outpatient department can only accommodate four people while there are almost a hundred or ninety people per day so you find that people are just standing. [#1, Dar es Salaam, Mental health clinician]

We don't have enough facilities within the hospital, nurses face some challenges also. When you go to [name] ward, some peers are sleeping down and even divide blankets. You may find that one blanket is divided among 2 to 4 patients. [#13, Kampala, PSW]

Theme 3: Organisational culture

Participants mainly in higher income countries reported that working inside structured and hierarchal systems can create a feeling of indebtedness to the organisation which can impact on PSWs autonomy in decision-making and contribute towards feelings of disempowerment.

When you enter a job as a consumer provider, at least in the beginning, there is part of you that feels like they are doing you a favour that they hired you. That you have to do what the organisation tells you to do in order to gain experience etc. [#8, Be'er Sheva, PSW]

For peer review only - http://bmjopen.bmj.com/site/about/guidelines.xhtml

For successful implementation of peer support work, PSWs should work by the following the rules that have been set up in the organization and the organization should also adjust its system to accommodate the peer support worker.

> *The PSW needs to understand that he is coming here as a worker and needs to follow the expectations like any other worker. As a worker he is also entitled to some sort of accommodation system, for example it might be difficult for them to start work in the mornings because they have to take pills, so he will start at 10:00 and not 7:30. So he'll do more afternoon shifts as opposed to morning ones. [#2,* Be'er Sheva, Mental Health Clinician]

Participants highlighted that the formalisation of the PSW role in such defined systems raises the question of how much of the role should remain informal versus formal in order for PSWs to fit in.

> *I also think that what especially happens with peer specialists, is some sort of formalization of this thing, and how much do we really actually want to formalize it. And how much of it do we have to keep informal, which is one of the worries or dilemmas. The ideals that are really inside of this system that is so formal, and is hierarchical and clear.* [#3, Be'er Sheva, PSW]

In lower income countries, participants reported that the support can be very limited due to the lack of psychological and social support resources available.

> *In summary, there is diagnosis, treatment mostly pharmacological using medical treatment. [We] do our best to try to provide psychological and social support but those are very limited most of the time.* [#30, Kampala, Manager]

Theme 4: Role definition

Having a clear role definition and expectations were reported as important, because without this reference point it can lead to potential role confusion and uncertainty.

> *I don't think it's that easy. They often don't know what they can do themselves. That they also have ideas, what can I actually do now? And I don't think there were enough guidelines or terms of reference.* [#11, Ulm, Mental health clinician]

In addition, the wide variety of tasks PSWs can perform means it can be difficult to construct a role description that accurately fits with real-life peer support practice.

> *The task fields of peer support workers are totally different. That is always person-dependent. We tried to create a kind of job description already and that was very, very difficult. Because we didn't want to restrict the peer support workers too much. Since the tasks always depend on the personality.* [#2, Ulm, Mental health clinician]

Theme 5: Training and Support

Training to peer support workers and health workers is an important factor for successful implementation of PSWs roles. Some participants in both lower and higher income countries described that peer support service is something which is new to other mental health professionals, some of them lack knowledge on what peer support work is. Training health care workers and PSWs will help in reducing uncertainties among professionals.

For peer review only - http://bmjopen.bmj.com/site/about/guidelines.xhtml

For peer review only

> *Peer support work is something new in other countries, it needs to be introduced to other staff members in a larger scale. There is also a simple lack of knowledge, not just supervision, but knowledge of what it is all about, and this lack of knowledge also leads to uncertainty among professionals. What, how do we deal with it now, what do we trust them to do, what do we take away from them because it is not the right thing to do? [#11, ULM, Mental health clinician]*

The availability of training enables PSWs to know what is expected from them and also understand their needs.

> *The first thing is that they are supposed to receive is training. If they receive training they will know their job description and the techniques of going to the families because there are families which don't want people to know that they have a mental patient.* [#3, Dar es Salaam, Mental health clinician]

> *I can say that the PSW program that I was part of had PSWs who first of all received training especially to understand their needs, making sure they are dealing with mental illness of others and also how they work with PSWs.* [#30, Kampala, Manager]

Participants in both lower and higher income countries described how PSWs need support to maintain their wellbeing and carry out their roles. Guidance and supervision from health care workers is very important for PSWs.

> *They shouldn't always work in isolation; they should be supervised.* [#24, Kampala, Mental health clinician]

> *Peer support workers can't be independent, they need professional community nurses to guide them so they can go out in the field, they can be together.* [#30, Kampala, Manager]

> *Coming from a person who is currently dealing with very active symptoms with varying levels of force, a person needs…regulatory capacity, the ability to manage workloads, the ability to receive help, to be helped and to defend oneself.* [#1, Be'er Sheva, Mental health clinician]

Participants identified the need for initial and further training in boundaries, code of conduct, and levels of disclosure.

> *Peer support workers need more training, continuous training. Even if the training is a one-off. So this should be happening. It shouldn't be a big deal.* [#30, Kampala, Manager]

Theme 6: Peer support network
Participants in both lower and higher income countries described that having peer support workers access to a peer network enables them to address their potential challenges, made them to feel connected through sharing their experiences and also enabled them to feel stronger together.

For peer review only - http://bmjopen.bmj.com/site/about/guidelines.xhtml

I can see how a group of peers impact each other non-stop and advances processes almost as if it is a race, but not in a bad way. That is to say, not from a place where you feel that they are forcing you to run, but from a place where a lot of people who are together all the time, are shattering stigmas about one another, I think that a group is stronger. [#3, Be'er Sheva, PSW]

Through these networks PSWs get to know each other well, and can identify if another PSW was facing a problem, such networks enabled PSWs to improve their well-being.

We peers have what they call buddies. A buddy is a person who knows more about you whereby in case you show signs of relapse that buddy will say '(name) is getting a relapse, (name) do this and this'. He will help you and bring you medical personnel and overcome the situation. [#11, Kampala, PSW]

Discussion

Our study identified six influences on PSW implementation. At the societal level, community stigma and lay beliefs about mental conditions were influential. At the organisational level, the inter-linked themes of resource allocation and organisational culture were identified, as well as staff attitudes and the challenge of role clarity. At the PSW level, both adequate training /support and a strong peer support network were facilitators of implementation.

Two aspects of our findings are noteworthy in relation to other studies. First, the conceptual framework was developed on the basis of research almost exclusively from high-income countries, and identified PSW and organisational influences. The implementation influences identified by participants had a stronger emphasis on societal aspects, including attitudes and role assumptions. Our findings are consistent with the previously-discussed systematic review,[21] published since the conceptual framework was developed. This validates the importance of considering organisational and specifically societal aspects when implementing PSW in different resource settings.

This involves developing community awareness regarding the value of peer support, to gain the support of family and community members.[26] Second, the PSW-level influences indicate the need to modify how PSW is provided in different settings. A systematic review of 39 studies, only one from a lower income setting, identified seven types of modification to the PSW role,[27] including recruitment processes, role expectations, training and support. Recent research is expanding to also consider staff attitudes,[28] organisational integration of PSWs,[29,30] _ENREF_32 organisational climate[31] and context.[32,33]

The primary implication is that more attention needs to be paid to societal attitudes and organisational culture in developing and implementing PSW programmes. Discrimination and stigma relating to mental health are global challenges,[4] but our findings suggest that there is a relationship between community attitudes and the ability to involve people with lived experience in the mental health workforce as PSWs. In terms of organisational culture, the findings reinforce existing evidence[34] that organisational culture impacts on recovery support, so organisational transformation may be needed.[35] Approaches to supporting culture change within mental health services include the introduction of pro-recovery interventions,[36,37] development of adjunctive services such as Recovery Colleges,[38] working with teams[39] and introducing co-production[40] and growth-oriented approaches.[41]

A better understanding of the relationship between the identified influences is needed. In UPSIDES, the theory of change technique is being used to map out different steps in the

For peer review only - http://bmjopen.bmj.com/site/about/guidelines.xhtml

implementation of the PSW intervention, and to articulate the connections between these steps. The impact of societal and organisational influences on PSW effectiveness will be illuminated in the multi-national UPSIDES randomised controlled trial (ISRCTN26008944) which is currently underway.[42]

The strengths of the study include the sample size, the use of multiple informants, conducting local language topic guides to avoid excluding non-English speaking participants, and the multi-national sampling frame. Credibility of the findings was enhanced by independent coding and the use of multiple analysts.

Several limitations can be identified. One significant shortcoming is that sociodemographic characteristics of participants were not collected in a standardised way across all sites, limiting the transferability of findings. Whilst the sample is large for a qualitative study, the findings are complex and nuanced, so our analysis focused on semantic rather than latent coding[25]. Future analysis might explore the relationship between the identified implementation influences, such as how community attitudes may distally impact on resource allocation. Whilst the use of analysts with different professional backgrounds reduced researcher influence on findings, the credibility of the findings could be enhanced by member checking, and including people with lived experience as co-analysts.[43] Finally, the relatively small number of policy-maker participants may account for the limited mention of national and regional policy as an influence.

Conclusions

This is the first multi-country study to explore societal attitudes and organisational culture influences on the implementation of peer support. Addressing community-level discrimination and developing a recovery orientation in mental health systems can contribute to effective implementation of peer support work.

For peer review only - http://bmjopen.bmj.com/site/about/guidelines.xhtml

For peer review only

Acknowledgements

The Using Peer Support In Developing Empowering Mental Health Services (UPSIDES) Study is a multicentre collaboration between the Department for Psychiatry and Psychotherapy II at Ulm University, Germany (Bernd Puschner, coordinator); the Institute of Mental Health at University of Nottingham, UK (Mike Slade); the Department of Psychiatry at University Hospital Hamburg-Eppendorf, Germany (Candelaria Mahlke); Butabika National Referral Hospital, Uganda (David Basangwa); the Centre for Global Mental Health at London School of Hygiene and Tropical Medicine, UK (Grace Ryan); the Department of Health Systems, Impact Evaluation and Policy, Ifakara Health Institute, Dar es Salaam, Tanzania (Donat Shamba); the Department of Social Work at Ben Gurion University of the Negev, Be'er Sheva, Israel (Galia Moran); and the Centre for Mental Health Law and Policy, Pune, India (Jasmine Kalha).

Contributors statement

MR, AC contributed to data acquisition, analysis and interpretation and drafting the manuscript. AG, RH, RN, AK, JK, CM, GM, RM, AMS, GR, DS and MS contributed to the design of the work, data acquisition, analysis and interpretation, and critically revised the work for important intellectual content. MR, AC, AG, RH, RN, AK, JK, CM, GM, RM, AMS, GR, DS and MS gave final approval of the version to be published, and are accountable for all aspects of the work.

Funding

This work was supported by the European Union Horizon 2020 Research and Innovation Programme under Grant Agreement No 779263. MS acknowledges the support of the Center for Mental Health and Substance Abuse, University of South-Eastern Norway and the NIHR Nottingham Biomedical Research Centre. This publication reflects only the authors' views. The Commission is not responsible for any use that may be made of the information it contains. The funding body had no role in the design of the study and in writing the manuscript.

Competing Interests

None declared.

Data sharing statement

Data are available upon reasonable request.

For peer review only

For peer review only - http://bmjopen.bmj.com/site/about/guidelines.xhtml

References

1. Samele C, Frew, S., Urquia, N. European profile of prevention and promotion of mental health (EuroPoPP-MH): EU Health Programme [(EAHC/2010Health/04)]; 2013.
2. Pathare S, Brazinova A, Levav I. Care gap: a comprehensive measure to quantify unmet needs in mental health. *Epidemiology and Psychiatric Sciences* 2018; **27**: 463-7.
3. Collins PY, Patel V, Joestl SS, March D, Insel TR, Daar AS. Grand challenges in global mental health. *Nature* 2011; **475**(7354): 27-30.
4. Patel V, Saxena S, Lund C, et al. The Lancet Commission on global mental health and sustainable development. *Lancet* 2018; **392**: 1553-98.
5. Misra S, Stevenson A, Haroz E, de Menil V, Koenen K. 'Global mental health': systematic review of the term and its implicit priorities. *Br J Psychiatry* 2019; **5**: e47.
6. Davidson L, Bellamy C, Guy K, Miller R. Peer support among persons with severe mental illnesses: a review of evidence and experience. *World Psychiatry* 2012; **11**: 123-8.
7. Mead S. Intentional Peer Support: an alternative approach. Plainfield, NH: Shery Mead Consulting; 2005.
8. Pitt V, Lowe D, Hill S, et al. Consumer-providers of care for adult clients of statutory mental health services. *Cochrane Database of Systematic Reviews* 2013; **3**: CD004807.
9. Lloyd-Evans B, Mayo-Wilson E, Harrison B, et al. A systematic review and meta-analysis of randomised controlled trials of peer support for people with severe mental illness. *BMC Psychiatry* 2014; **14**: 39.
10. Shalaby R, Agyapong V. Peer Support in Mental Health: Literature Review. *JMIR Mental Health* 2020; **7**: e15572.
11. Chinman M, George, P., Dougherty, R., Daniels, A., Ghose, S., Swift, A., Delphin-Rittmon, M. Peer Support Services for Individuals With Serious Mental Illnesses: Assessing the Evidence. *Psychiatr Serv* 2014; **65**: 429-41.
12. White S, Foster R, Marks J, et al. The effectiveness of one-to-one peer support in mental health services: a systematic review and meta-analysis. *BMC Psychiatry* 2020; **20**: 534.
13. Repper J. ImROC Briefing Paper 5. Peer Support Workers: Theory and Practice. London: Centre for Mental Health; 2013.
14. Mead S, Hilton, D., Curtis, L. Peer support: A theoretical perspective. *Psychiatric Rehabilitation Journal* 2001; **25**: 134–41.
15. Fan Y, Ma N, Ma L, Xu W, Lamberti JS, Caine E. A community-based peer support service for persons with severe mental illness in China. *BMC Psychiatry* 2018; **18**: 170.
16. Pathare S, Kalha J, Krishnamoorthy S. Peer support for mental illness in India: An underutilised resource. *Epidemiology and Psychiatric Sciences* 2018; **27**: 415-9.
17. Moran GS. The mental health consumer movement and peer providers in Israel. *Epidemiology and Psychiatric Sciences* 2018; **27**: 420-6.
18. Lee YY, Ang S, Chua HC, Subramaniam M. Peer Support in Mental Health: A Growing Movement in Singapore. *Annals of Academy of Medicine Singapore* 2019; **48**: 95-7.
19. Ryan G, Kamuhiirwa M, Mugisha J, et al. Peer support for frequent users of inpatient mental health care in Uganda: protocol of a quasi-experimental study. *BMC Psychiatry* 2019; **19**: 374.
20. Piat M, Sofouli E, Sabetti J, et al. Protocol for a mixed studies systematic review on the implementation of the recovery approach in adult mental health services. *BMJ Open* 2017; **7**(8).
21. Ibrahim N, Thompson D, Nixdorf R, et al. A systematic review of influences on implementation of peer support work for adults with mental health problems. *Soc Psychiatry Psychiatr Epidemiol* 2020; **55**: 285-93.

For peer review only - http://bmjopen.bmj.com/site/about/guidelines.xhtml

22. Puschner B, Repper J, Mahlke C, et al. Using Peer Support in Developing Empowering Mental Health Services (UPSIDES): Background, rationale and methodology. *Annals of Global Health* 2019; **85**(53): 1-10.
23. Jabareen Y. Building a Conceptual Framework: Philosophy, Definitions, and Procedure. *International Journal of Qualitative Methods* 2009; **8**: 49-62.
24. Repper J, Perkins R. ImROC Briefing Paper 6. The Team Recovery Implementation Plan: a framework for creating recovery-focused services. London: Centre for Mental Health; 2013.
25. Braun V, Clarke V, Hayfield N, Terry G. Thematic analysis. In: Laiamputtong L, ed. Handbook of Research Methods in Social Sciences. Singapore: Springer; 2019: 843-60.
26. Pearson H, Adams B, Eaton J, Illingworth M. Community Mental Health Good Practice Guide: Peer Support: CBM Global Disability Inclusion; 2021.
27. Charles A, Thompson D, Nixdorf R, et al. A typology of modifications to peer support work for adults with mental health problems: systematic review. *Br J Psychiatry* 2020; **216**: 301-7.
28. Moore T, Zeeman L. More 'milk' than 'psychology or tablets': Mental health professionals' perspectives on the value of peer support workers. *Health Expectations* DOI: 10.1111/hex.13151.
29. Charles A, Nixdorf R, Ibrahim N, et al. Initial training for mental health peer support workers: international Delphi Consultation. *JMIR Mental Health* 2021; **5**: e25528.
30. Otte I, Werning A, Nossek A, Vollmann J, Juckel G, Gather J. Challenges faced by peer support workers during the integration into hospital-based mental health-care teams: Results from a qualitative interview study. *Int J Soc Psychiatry* 2020; **66**: 263-9.
31. Jones N, Teague G, Wolf J, Rosen C. Organizational Climate and Support Among Peer Specialists Working in Peer-Run, Hybrid and Conventional Mental Health Settings. *Administration and Policy in Mental Health and Mental Health Services Research* 2020; **47**: 150-67.
32. Adams W. Unintended consequences of institutionalizing peer support work in mental healthcare. *Soc Sci Med* 2020; **262**: https://doi.org/10.1016/j.socscimed.2020.113249.
33. Urichuk L, Hrabok M, Hay K, et al. Enhancing peer support experience for patients discharged from acute psychiatric care: protocol for a randomised controlled pilot trial. *BMJ Open* 2018; **8**: e022433.
34. Clossey L, Rheinheimer D. Exploring the effect of organizational culture on consumer perceptions of agency support for mental health recovery. *Community Ment Health J* 2014; **50**(4): 427-34.
35. Le Boutillier C, Chevalier A, Lawrence V, et al. Staff understanding of recovery-orientated mental health practice: a systematic review and narrative synthesis. *Implementation Science* 2015; **10**: 87.
36. Meadows G, Brophy L, Shawyer F, et al. REFOCUS-PULSAR recovery-oriented practice training in specialist mental health care: a stepped-wedge cluster randomised controlled trial. *Lancet Psychiatry* 2019; **6**: 103-14.
37. Slade M, Bird V, Clarke E, et al. Supporting recovery in patients with psychosis through care by community-based adult mental health teams (REFOCUS): a multisite, cluster, randomised, controlled trial. *Lancet Psychiatry* 2015; **2**: 503-14.
38. Crowther A, Taylor A, Toney R, et al. The impact of Recovery Colleges on mental health staff, services and society. *Epidemiology and Psychiatric Sciences* 2019; **28**: 481-8.
39. Vacher G. Utilising Team Recovery Implementation Plan (TRIP): embedding recovery-focused practice in rehabilitation services. *Mental Health and Social Inclusion* 2017; **21**: 240-7.
40. Lewis A, King T, Herbert L, Repper J. Co-Production – Sharing Our Experiences, Reflecting On Our Learning. Nottingham: ImROC; 2017.
41. Slade M, Rennick-Egglestone S, Blackie L, et al. Post-traumatic growth in mental health recovery: qualitative study of narratives. *BMJ Open* 2019; **9**: e029342.

For peer review only - http://bmjopen.bmj.com/site/about/guidelines.xhtml

42. Moran G, Kalha J, Mueller-Stierlin A, et al. Peer support for people with severe mental illness versus usual care: study protocol for a pragmatic multicentre randomised controlled trial (UPSIDES-RCT). *Trials* 2020; **21**: 371.
43. Jennings H, Slade M, Bates P, Munday E, Toney R. Best practice framework for Patient and Public Involvement (PPI) in collaborative data analysis of qualitative mental health research: methodology development and refinement. *BMC Psychiatry* 2018; **18**: 213.

For peer review only

For peer review only - http://bmjopen.bmj.com/site/about/guidelines.xhtml

Table 1: Conceptual framework for PSW principles (n=8) and societal / organisational implementation influences (n=5)

Principle	Definition
1. Mutual	Peer support workers have similar experiences to peer support users
2. Reciprocal	Peer support workers and peer support users both give and receive in the relationship
3. Non-directive	Peer support workers develop solutions together with the peer support user, instead of dictating solutions
4. Recovery focused	Peer support workers support the peer support user on his/her path towards overcoming the problems that they experience
5. Strengths-based	Peer support workers show a positive attitude and identify and build on the strengths and recovery progress of peer support users
6. Inclusive	Peer support workers do not exclude people on the basis of the nature of their problems or beliefs about their level of ability, and help peer support users to find their place in society
7. Progressive	Peer support workers and users advance together towards recovery, this is not a befriending relationship that aims to maintain current progress
8. Safe	Peer support workers and users develop a common basis of trust and safety, which is central to the planning of the service and training of peer workers
**Implementation influence**	**Description of societal / organisational influence**
1. Group versus individual	Peer support can be offered in single sessions and in a group setting
2. Extent to which both parties choose to enter the relationship	Peer support pairs and groups can be formed by the organisation, but also by the peers themselves
3. Extent to which rules govern the relationship	There can be implicit and explicit rules underpinning how the peer support work is conducted
4. Extent to which the parties involved are in the same place in their recovery journey	Depending on the state of recovery, peer support users can become peer support workers and vice versa
5. Extent to which the peer support workers focus on peer support users	Peer support workers can support recovery for peer support users and/or promote a recovery orientation for the staff they work with, the institution they work in, and the society they live in

For peer review only - http://bmjopen.bmj.com/site/about/guidelines.xhtml

Table 2: Focus group participants (n=86)

Site	Focus groups	n	Participant				Gender	
			Potential or actual PSW	Mental health worker	Mental health manager	Policy-maker	Male	Female
Ulm	2	12	1	10	0	1	4	8
Hamburg	2	12	7	5	0	0	4	8
Kampala	4	32	16	14	1	1	10	22
Dar es Salaam	2	16	0	12	4	0	7	9
Be'er Sheva	2	14	2	8	4	0	5	9
**Total**	**12**	**86**	**26**	**49**	**9**	**2**	**30**	**56**

For peer review only

Figure 1: Summary of findings

Figure legend: Figure 1 shows the societal and organisation influences which are important for the implementation of mental health peer support work. This includes community and staff attitudes, availability of resources, organisational culture, having clear role definition, training and support and access to a peer support network.

For peer review only

For peer review only - http://bmjopen.bmj.com/site/about/guidelines.xhtml

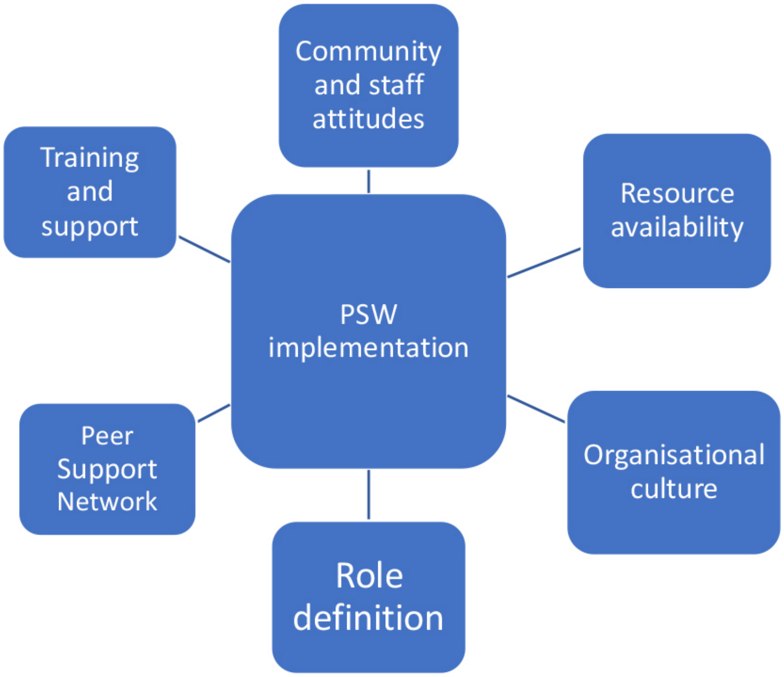

Figure 1: Summary of findings

Figure 1 shows the societal and organisation influences which are important for the implementation of mental health peer support work. This includes community and staff attitudes, availability of resources, organisational culture, having clear role definition, training and support and access to a peer support network.

90x90mm (300 x 300 DPI)

Instructions:

This guide is to be used to facilitate focus group discussion with (1.1) health workers who are using or plan to recruit peer support workers. (Psychiatric nurses, clinicians working in mental health ward / departments) and (1.2) local stakeholders with relevant expertise relating to implementation of peer work (including clinicians and managers who currently, previously or in the future may employ peer support workers, and people who currently, previously or in the future may work as peer support workers).

Interviewer ID ___ ___ ___ ___

Interview date (DD/MM/YYYY): __ __ / __ __ / __ __ __

Location of the interview ______________________________

Country: ______________________________

Introduction:

Ensure that the participants are comfortable.

Hi and welcome to this session. My name is ___ and my colleague is___. Thank you for taking your time to talk to us about your experiences with people with severe mental illness and peer support workers. We will discuss topics related to the key characteristics of peer support workers and the challenges they may face. The discussion will take around 60minutes. The interview will be recorded using an audio recorder as we will not have to write down all your answers and we will not miss any of your useful comments. Informed consent will be obtained from all the participants and confidentiality will be assured to all the participants. Before we begin, I would like to know if you have any questions.

Explain you are starting the audio recorder.

Time interview started:

Theme 1: Socio-demographic information of the respondents

Collect data on name, age, education, position and number of years working in this position and give a code to each participant.

Explain again to the participant that:

- I want to learn about your experience, thoughts and perspective on this topic of using peer support workers for improving mental health conditions in your country.
- There is no 'right' or 'wrong' answer.

Say: We are planning to recruit and train peer support workers. Peer providers in mental health are individuals with severe mental illness who are further along in their recovery, who support others with similar conditions by role modelling that recovery is possible, sharing knowledge from experience and using reciprocal empathic relationships. We are thinking of asking these peer support workers to identify and visit individuals with severe mental illness. We would like to get your opinion on how best to implement PSWs intervention in this region. We would like to know key characteristics for PSWs and challenges that could hinder them performing their

For peer review only - http://bmjopen.bmj.com/site/about/guidelines.xhtml

tasks. Please be frank as we want the information to be the right ones for this community.

Theme 2: Social cultural factors and acceptability of PSWs

1. Based on your experiences, how does the community care for people with SMIs? Where do they seek care? Are there other places apart from the hospital? Describe all the places people seek care for SMI.

2. What are your main roles in supporting people with SMIs? What are the main challenges that you are facing in doing your job? What are the solutions to those challenges?

    - Probe to know if mental health programmes are given a priority?
    - Is there a national policy or guideline for mental health conditions? Are these accessible?
    - What has been the main challenge your department has been facing in managing/meeting the needs of people with Severe Mental Illness? Probe for various challenges the department has been facing in regard to SMI programmes? For each challenge mentioned, ask how the department could handle it i.e. any potential solution and/or suggestion on how to address it?

3. With your experience working in this area, could you please tell us what do you know about PSWs and how do they perform their duties?

    - Probe for, how they are recruited, who recruits them, what are they exactly doing, etc?
    - Please, describe any systems for linking people with peers who can serve as role models in this facility or community? (e.g. through contact with local user-run groups).

4. What key qualities would you want a peer support worker to have or NOT to have? We want to find out what sort of person the respondents would trust and value, perform the work well and responsibly.

5. Could you please tell us what are the specific things, you would want a peer support worker to do?

    Probe for specific terminologogies, main activities, duration of activities. care planning

Theme 3: Institution / facility readiness to incorporate PSW

6. In your opinion – for using peer support workers, how could it be implemented / improved at this facility?

For peer review only - http://bmjopen.bmj.com/site/about/guidelines.xhtml

- Probe: training, logistics, management, supervision, monitoring, capacity development, resources etc.

7. What are the facilitators and barriers in providing peer support to people with SMI in your facility or in your region?

    - Probe for resources, skilled personnel, infrastructure, systems support?

8. Is there anything more you would like to add about your experiences with, or views on, using peer support workers?

Time interview end:

Thank the participant for his / her time. Remind them that the information will be kept confidential.

Interviewer comments on how the interview went:

For peer review only

For peer review only - http://bmjopen.bmj.com/site/about/guidelines.xhtml

Standards for Reporting Qualitative Research Checklist

Item	Topic	Where addressed
**Title and abstract**		
S1	**Title**<br>Concise description of the nature and topic of the study Identifying the study as qualitative or indicating the approach (e.g., ethnography, grounded theory) or data collection methods (e.g., interview, focus group) is recommended	Title
S2	**Abstract**<br>Summary of key elements of the study using the abstract format of the intended publication; typically includes background, purpose, methods, results, and conclusions	Abstract
**Introduction**		
S3	**Problem formulation** Description and significance of the problem/phenomenon studied; review of relevant theory and empirical work; problem statement	Introduction
S4	Purpose or research question Purpose of the study and specific objectives or questions	Introduction final paragraph
**Methods**		
S5	**Qualitative approach and research paradigm**<br>Qualitative approach (e.g., ethnography, grounded theory, case study, phenomenology, narrative research) and guiding theory if appropriate; identifying the research paradigm (e.g., postpositivist, constructivist/interpretivist) is also recommended; rationale	Methods Design<br>Method Procedures paragraph 2<br>Methods Analysis
S6	**Researcher characteristics and reflexivity**<br>Researchers' characteristics that may influence the research, including personal attributes, qualifications/experience, relationship with participants, assumptions, and/or presuppositions; potential or actual interaction between researchers' characteristics and the research questions, approach, methods, results, and/or transferability	Methods Analysis<br>Discussion paragraph 2
S7	**Context**	Methods Setting

For peer review only

	Setting/site and salient contextual factors; rationale	
S8	**Sampling strategy**<br>How and why research participants, documents, or events were selected; criteria for deciding when no further sampling was necessary (e.g., sampling saturation); rationale	Methods Participants<br>Methods Procedures final paragraph<br>Methods Sampling
S9	**Ethical issues pertaining to human subjects** Documentation of approval by an appropriate ethics review board and participant consent, or explanation for lack thereof; other confidentiality and data security issues	Methods Ethics approval
S10	**Data collection methods** Types of data collected; details of data collection procedures including (as appropriate) start and stop dates of data collection and analysis, iterative process, triangulation of sources/methods, and modification of procedures in response to evolving study findings; rationale. The rationale should briefly discuss the justification for choosing that theory, approach, method, or technique rather than other options available, the assumptions and limitations implicit in those choices, and how those choices influence study conclusions and transferability. As appropriate, the rationale for several items might be discussed together.	Methods Procedures<br>Methods Analysis
S11	**Data collection instruments and technologies**<br>Description of instruments (e.g., interview guides, questionnaires) and devices (e.g., audio recorders) used for data collection; if/how the instrument(s) changed over the course of the study	Methods Procedures paragraphs 1 and 2
S12	**Units of study**<br>Number and relevant characteristics of participants, documents, or events included in the study; level of participation (could be reported in results)	Results Table 1
S13	**Data processing**<br>Methods for processing data prior to and during analysis, including transcription, data entry, data management and security, verification of data integrity, data coding, and anonymization/deidentification of excerpts	Methods Procedures final paragraph
S14	**Data analysis**<br>Process by which inferences, themes, etc., were identified and developed, including the researchers involved in data analysis; usually references a specific paradigm or approach; rationale	Methods Analysis

S15	**Techniques to enhance trustworthiness** Techniques to enhance trustworthiness and credibility of data analysis (e.g., member checking, audit trail, triangulation); rationale	Methods Analysis Discussion strengths and limitations paragraph
**Results/findings**		
S16	**Synthesis and interpretation** Main findings (e.g., interpretations, inferences, and themes); might include development of a theory or model, or integration with prior research or theory	Results
S17	**Links to empirical data** Evidence (e.g., quotes, field notes, text excerpts, photographs) to substantiate analytic findings	Results
**Discussion**		
S18	**Integration with prior work, implications, transferability, and contribution(s) to the field** Short summary of main findings; explanation of how findings and conclusions connect to, support, elaborate on, or challenge conclusions of earlier scholarship; discussion of scope of application/generalizability; identification of unique contribution(s) to scholarship in a discipline or field	Discussion
S19	**Limitations** Trustworthiness and limitations of findings	Discussion paragraph 2
**Other**		
S20	**Conflicts of interest** Potential sources of influence or perceived influence on study conduct and conclusions; how these were managed	Competing Interests statement
S21	**Funding** Sources of funding and other support; role of funders in data collection, interpretation, and reporting	Funding statement

For peer review only

BMJ Open

Societal and organisational influences on implementation of mental health peer support work in low-income and high-income settings: a qualitative focus group study

Journal:	*BMJ Open*
Manuscript ID	bmjopen-2021-058724.R3
Article Type:	Original research
Date Submitted by the Author:	26-Dec-2022
Complete List of Authors:	Ramesh, Mary; Ifakara Health Institute, Department of Health System, Impact Evaluation and Policy<br>Charles, Ashleigh; University of Nottingham, School of Health Sciences<br>Grayzman, Alina; Ben-Gurion University of the Negev Faculty of Health Sciences, Department of Social Work, Ben Gurion University of the Negev<br>Hiltensperger, Ramona; Department of Psychiatry II<br>Kalha, Jasmine; Indian Law Society<br>Kulkarni, Arti; Indian Law Society<br>Mahlke, Candelaria ; University Medical Cente Hamburg, Candelaria Irene Mahlke<br>Moran, Galia; Ben-Gurion University of the Negev, Department of Social Work<br>Mpango, Richard; Butabika National Referral Hospital; Soroti University<br>Stierlin, Annabel; Ulm University, Institute of Epidemiology and Medical Biometry<br>Nixdorf, Rebecca ; University Medical Centre Hamburg-Eppendorf, Department of Psychiatry<br>Ryan, Grace; London School of Hygiene and Tropical Medicine, Population Health<br>Shamba, Donat ; Ifakara Health Institute, Department of Health Systems, Impact Evaluation and Policy<br>Slade, Mike; University of Nottingham School of Health Sciences
**Primary Subject Heading**:	Global health
Secondary Subject Heading:	Global health, Mental health, Qualitative research
Keywords:	MENTAL HEALTH, QUALITATIVE RESEARCH, PUBLIC HEALTH

SCHOLARONE™
Manuscripts

I, the Submitting Author has the right to grant and does grant on behalf of all authors of the Work (as defined in the below author licence), an exclusive licence and/or a non-exclusive licence for contributions from authors who are: i) UK Crown employees; ii) where BMJ has agreed a CC-BY licence shall apply, and/or iii) in accordance with the terms applicable for US Federal Government officers or employees acting as part of their official duties; on a worldwide, perpetual, irrevocable, royalty-free basis to BMJ Publishing Group Ltd ("BMJ") its licensees and where the relevant Journal is co-owned by BMJ to the co-owners of the Journal, to publish the Work in this journal and any other BMJ products and to exploit all rights, as set out in our licence.

The Submitting Author accepts and understands that any supply made under these terms is made by BMJ to the Submitting Author unless you are acting as an employee on behalf of your employer or a postgraduate student of an affiliated institution which is paying any applicable article publishing charge ("APC") for Open Access articles. Where the Submitting Author wishes to make the Work available on an Open Access basis (and intends to pay the relevant APC), the terms of reuse of such Open Access shall be governed by a Creative Commons licence – details of these licences and which Creative Commons licence will apply to this Work are set out in our licence referred to above.

Other than as permitted in any relevant BMJ Author's Self Archiving Policies, I confirm this Work has not been accepted for publication elsewhere, is not being considered for publication elsewhere and does not duplicate material already published. I confirm all authors consent to publication of this Work and authorise the granting of this licence.

For peer review only

Societal and organisational influences on implementation of mental health peer support work in low-income and high-income settings: a qualitative focus group study

Mary Ramesh [1 a]
Ashleigh Charles [2 a]
Alina Grayzman [3]
Ramona Hiltensperger [4]
Jasmine Kalha [5]
Arti Kulkarni[5]
Candelaria Mahlke [6]
Galia Moran[3]
Richard Mpango [7 8 9]
Annabel Sandra Müller-Stierlin [4]
Rebecca Nixdorf [6]
Grace Ryan [10]
Donat Shamba [1 b *]
Mike Slade [2 b]

[1] Department of Health Systems, Impact Evaluation and Policy, Ifakara Health Institute, Dar es Salaam, Tanzania
[2] School of Health Sciences, Institute of Mental Health, University of Nottingham, UK
[3] Department of Social Work, Ben Gurion University of the Negev, Be'er Sheva, Israel
[4] Department of Psychiatry II, Ulm University, Ulm, Germany
[5] Centre for Mental Health Law and Policy, Indian Law Society, Pune, India
[6] Department of Psychiatry, University Medical Center Hamburg-Eppendorf, Germany
[7] Butabika National Referral Hospital, Uganda
[8] School of Health Sciences, Soroti University, Soroti, Uganda
[9] MRC/UVRI and LSHTM Uganda Research Unit, Entebbe, Uganda
[10] Centre for Global Mental Health, London School of Hygiene and Tropical Medicine, UK

[a] Ramesh and Charles contributed equally and are joint first authors
[b] Shamba and Slade contributed equally and are joint last authors

*Correspondence to:
Donat Shamba
dshamba@ihi.or.tz

ORCID iDs
MR: 0000-0002-4942-3923; AC: 0000-0003-2222-4358; AG 0000-0002-8192-7697; RH: 0000-0003-2544-4188; JK: 0000-0001-7357-2366; AK: 0000-0002-3281-4388; CM: 0000-0001-9573-6106;GM: 0000-0001-9718-1773; RM: 0000-0001-6960-3174; AMS: 0000-0003-2812-5115; RN: 0000-0002-3064-8380; GR: 0000-0002-9310-3513; DS: 0000-0001-7431-7199; MS: 0000-0001-7020-3434

Word count
5231 words.

For peer review only - http://bmjopen.bmj.com/site/about/guidelines.xhtml

ABSTRACT

Objectives
Despite the established evidence base for mental health peer support work, widespread implementation remains a challenge. This study aimed to explore societal and organisational influences on the implementation of peer support work in low-income and high-income settings.

Design
Study sites conducted two focus groups in local languages at each site, using a topic guide based on a conceptual framework describing eight peer support worker (PSW) principles and five implementation issues. Transcripts were translated into English and an inductive thematic analysis was conducted to characterise implementation influences.

Setting
The study took place in two tertiary and three secondary mental health care sites as part of the Using Peer Support in Developing Empowering Mental Health Services (UPSIDES) study, comprising three high-income sites (Hamburg and Ulm, Germany; Be'er Sheva, Israel) and two low-income sites (Dar es Salaam, Tanzania; Kampala, Uganda) chosen for diversity both in region and in experience of peer support work.

Participants
12 focus groups were conducted (including a total of 86 participants), across sites in Ulm (n=2), Hamburg (n=2), Dar es Salaam (n=2), Be'er Sheva (n=2), and Kampala (n=4). Three individual interviews were also done in Kampala. All participants met the inclusion criteria: aged over 18 years; actual or potential peer support worker or mental health clinician or hospital / community manager or regional / national policy-maker; and able to give informed consent.

Results
Six themes relating to implementation influences were identified: community and staff attitudes; resource availability; organisational culture; role definition; training and support; and peer support network.

Conclusions
This is the first multi-country study to explore societal attitudes and organisational culture influences on the implementation of peer support. Addressing community-level discrimination and developing a recovery orientation in mental health systems can contribute to effective implementation of peer support work. The relationship between societal stigma about mental health and resource allocation decisions warrants future investigation.

Study registration number
ISRCTN26008944 (UPSIDES Study).

For peer review only

Strengths and limitations of this study

- The sample size (86 participants, across 12 focus groups) and sampling across two low-income and three high-income sites increases the credibility of the findings and their relevance to similar settings.
- Independent coding by multiple analysts from different cultures enhances trustworthiness.
- Sociodemographic characteristics were not sufficiently collected to be reported, limiting transferability of findings.
- Study participants were peer support workers and mental health professionals; there is a need to conduct further studies with service users or recipients of peer support services in order to understand their perceptions on the influences on the implementation of peer support work.
- Two focus group discussions per site may not reach saturation; however, the study involved different sets of respondents to bring together the perspectives of different groups who either had experience in peer support work or were planning to use peer support workers.

For peer review only

For peer review only - http://bmjopen.bmj.com/site/about/guidelines.xhtml

Introduction

Many people living with severe mental illness do not receive adequate care. For example, in Europe the EuroPoPP-MH study found that a comprehensive range of community-based services existed in only eight of 29 countries.[1] The resulting gap between demand and supply is called the treatment gap, or care gap.[2] While mental health has been identified as a global priority,[3] the mental health treatment gap remains, and is largest in low-resource settings.[4]

One reason for the treatment gap in low-resource settings is that mental health initiatives do not always sufficiently address contextual aspects, such as up-stream social determinants, geographic and linguistic differences, and sociodemographic influences such as ethnicity, caste and tribe.[5] This leads to barriers in receiving mental health treatment, including stigma, social exclusion and inequities in terms of resourcing.

Mental health peer support is an established intervention involving a person with lived experience of mental health problems and recovery employed to offer support to others with mental health problems. Peer support workers (PSWs) act as credible role models of recovery,[6] instilling hope through positive self-disclosure, modelling the use of experiential knowledge for self-care, and offering supportive relationships based on intentional mutuality.[7] There is a growing empirical evidence base for PSWs.[8-11] A recent systematic review identified 19 randomised controlled trials,[12] all from high-income countries. This review found PSW was associated with beneficial outcomes in relation to supporting recovery, empowerment and social networks. However, heterogeneity in the implementation of peer support was identified as an important knowledge gap.

Most research on mental health peer support work has been conducted in high-resource (typically Anglophone) settings, including creation of core PSW principles[13,14] and evaluation.[8] However, PSW roles are increasingly being developed, formalised, and implemented in more diverse settings, such as China,[15] India,[16] Israel,[17] Singapore[18] and Uganda_ENREF_24.[19] An important knowledge gap therefore exists in relation to PSW implementation influences across settings with different resource levels.[20] A recent systematic review synthesised 53 studies (none from low-income countries) to identify 14 influences on implementation of mental health PSW.[21] The most commonly reported influence was organisational culture, identified in 53% of studies, followed by training and role definition. Societal influences were also identified, including PSW access to a peer network, resource availability and financial arrangements.

The Grand Challenges for Mental Health initiative identified the importance of research along the translation continuum including implementation, and emphasised that implementation is a challenge not just in low- and middle-income countries.[3] In other words, the focus should be on implementation research including both lower- and higher-resource settings. To our knowledge, no study has explored PSW implementation across multiple countries. The aim of this study was to explore and characterise the societal and organisational influences on the implementation of mental health peer support work in low-income and high-income settings.

Methods

This study was conducted as part of UPSIDES (Using Peer Support in Developing Empowering Mental Health Services), a five-year (2018-2022) European Union–funded multinational study that aims to replicate and scale up peer support interventions for people with severe mental illness.[22]

Design

A qualitative research design informed by a critical realist perspective was used. A critical realist approach was chosen as it can help in identifying some of the underlying organisational and

For peer review only - http://bmjopen.bmj.com/site/about/guidelines.xhtml

societal influences of PSW implementation. Focus groups were selected over other data collection approaches to maximise breadth of data coverage.

Setting

Data were collected from five UPSIDES study sites. Sites were based in two high-income countries (Hamburg and Ulm sites, Germany; Be'er Sheva, Israel) and two low-income countries (Dar es Salaam, Tanzania [low resource setting at the time of data collection, re-banded in 2020 to lower-middle income country]; Kampala, Uganda), ensuring regional diversity (Europe, Eastern Mediterranean, sub-Saharan Africa). Sites were classified as low-resource settings because they are based in low-resource countries. As previously reported,[21] sites were also diverse in terms of their experience with peer support work, with two sites (Dar es Salaam, Ulm) having no or very little previous experience.

Participants

Participants were purposively selected to include stakeholders with different perspectives on PSW implementation: actual or potential PSWs, mental health clinicians or managers from hospitals or community services; and regional or national policy-makers. To be included, participants had to be over 18 years of age and capable of providing informed consent.

Participants came from a range of community-based, outpatient and inpatient mental health services in Germany, Uganda and Tanzania, and from a range of community mental health rehabilitation services in Israel. In all sites, multidisciplinary in-patient and out-patient care involves psychotherapy, psychosocial rehabilitation and psychiatric clinics, with some sites also offering family intervention, vocational skills training, cognitive enhancement therapy, psychoeducation, pre-discharge social interventions and physical health care.

Procedures

A conceptual framework – a network of interlinked concepts together providing a comprehensive understanding of a phenomenon[23] – was developed to capture the key elements and implementation influences on the PSW role. The conceptual framework comprised (a) PSW principles and (b) societal and organisational implementation influences, as shown in Table 1. The PSW principles were derived from a researcher-led integration of established core principles from high-resource settings.[13,14,24] At the time of development (2017) there was an absence of integrated evidence, so a systematic review was subsequently undertaken,[21] but for the current study the implementation influences were developed through consultation with experts in the UPSIDES consortium.

The conceptual framework informed the development of a topic guide (Supplement 1), comprising open-ended conversational prompts to explore the cultural applicability of PSW principles and to identify societal and organisational implementation influences. Exploration of areas of disagreement was encouraged, as was speculation about potential implementation influences in sites with no experience of PSW. The topic guide was developed in English, commented on by all sites, and then finalised and translated into Kiswahili (Dar es Salaam), German (Hamburg/Ulm), Hebrew (Be'er Sheva) and Luganda (Kampala).

Focus groups were conducted at each site between September and December 2018. In each site, potential participants were identified by mental health clinicians and UPSIDES research workers. Two focus groups were conducted per site, apart from Kampala where four focus groups (two for PSWs, two for other stakeholders) and three individual interviews were conducted. All focus groups were conducted in the local language and held in a health service or community venue.

For peer review only - http://bmjopen.bmj.com/site/about/guidelines.xhtml

Each focus group comprised five to nine participants, and lasted up to 60 minutes. Facilitators were UPSIDES research workers from the site, who were bilingual in the local language and English, and came from psychology, sociology, health sciences, social work and nursing backgrounds. All facilitators were experienced in qualitative data collection, and actively managed group dynamics to ensure full participation from all participants. Focus groups were recorded using an audio recorder and researchers took field notes during the discussions. After the focus groups, local language transcripts were made, with pseudonymisation of identifying information about participants and third parties. Each local language transcript was translated into English by the local UPSIDES researcher, and checked by the UPSIDES translation leads (Nottingham, UK and Pune, India) for data integrity, identifying points for site checking if needed. Finalised transcripts were password protected and uploaded to a restricted area on the UPSIDES website.

Ethics approval and participant informed consent

Ethical approval was obtained by each site: Ulm University Ethics Commission (Application nr. 195/18), Mengo Hospital Institutional Review Board Uganda (MH: 360; MH/REC/141/8/2018), National Institute for Medical Research Tanzania (NIMR/HQ/R.8a/Vol.IX/2982), Institutional Review Board, Ifakara Health Institute, Tanzania (IHI/IRB/No. 28 – 2018), Ärztekammer Hamburg, Germany (MC-230/18), and Human Subjects Research Committee of Ben-Gurion University (ref: 1621-2). Each potential participant was given an information sheet in the local language, and the opportunity to ask questions. All participants gave written informed consent before participation.

Analysis

A combination of deductive and inductive thematic analysis was conducted.[25] The two primary coders were UPSIDES research workers in Dar es Salaam (MR: background in public health) and Nottingham (AC: mental health nursing, sociology). MR and AC independently read all transcripts to familiarise themselves with the content and start the process of creating preliminary codes and categories. Coding was then discussed with site leads in Dar es Salaam (DS: social science) and Nottingham (MS: clinical psychology), following which a preliminary coding framework for implementation influences was developed. The codebook was then transferred into NVivo 12 software for coding. MR and AC independently coded the same four transcripts, and then discussed and reviewed any differences or discrepancies and any additional themes that emerged from the data. Following review, refinement, and defining of themes, an agreement was reached, and new codes were incorporated into the final coding framework. The remaining transcripts were then coded with repeated discussion between coders. The finalised coding framework was iteratively discussed amongst the four primary analysts (AC, MR, MS, and DS) and the wider author team until a consensus was reached.

Patient and public involvement

Individuals with lived experience are involved at multiple levels of the UPSIDES Study, including as part of the site team, as advisory board members, as peer support workers and as authors on some papers. No further patient and public involvement specific to the current study was undertaken.

Results

For peer review only - http://bmjopen.bmj.com/site/about/guidelines.xhtml

A total of 86 individuals participated across 12 focus groups. These include focus groups in Ulm (n=2), Hamburg (n=2), Dar es Salaam (n=2), Be'er Sheva (n=2), and Kampala (n=4, two for PSWs, two for other stakeholders). In addition to the focus groups, Kampala also conducted three individual interviews. Details are shown in Table 2.

Six implementation influence themes were identified: community and staff attitudes; resource availability; organisational culture; role definition; training and support; and peer support network.

Theme 1: Community and staff attitudes

Community and staff attitudes toward mental illness were perceived to be both a barrier and also a facilitator for PSWs to perform their roles. Some participants, especially in lower income countries, reported that people with mental health conditions are considered inferior and are also rejected, thus making it difficult for the PSWs to perform their roles effectively.

> *When it comes to class, mental health patients are considered second-hand, third-hand or fourth-hand citizens. So we are marginalised among the marginalised. We take the lowest rank status point in the community.* [#9, Kampala, PSW]

> Even in our community a person with a mental illness is not a priority. A large percent of our patients live in a community where there is stigma to the extent that they are not brought to the hospital. [#2, Dar es Salaam, Mental Health Clinician]

Furthermore, religious beliefs can also act as a barrier in implementing peer support work, as was apparent mainly in the lower-income countries.

> *There are so many religious leaders who believe that God doesn't fail. They interfere with our work. They stop our patients from taking medicine and they say that God is going to perform miracles then in the end they relapse. The traditional healers believe that mental illness is caused by traditional issues and they don't need Western medicine, they need herbs.* [#26, Kampala, PSW]

Also in the lower-income countries, PSWs reported experiencing rejection when they go to visit service users, as some family members do not want the mental health status of their relative to be revealed, thus making it difficult for the PSWs to perform their roles as described by a PSW from Uganda

> *We are rejected, you can go to that person's place who may not wish to see you and they don't welcome you and you can't insist. Sometimes they just avoid you.* [#12, Kampala, PSW]

For participants in lower income countries, community initiatives through arts, media, and local projects raised awareness in the community and educated people about mental health. These initiatives also enabled the PSWs to be known as role models in the community and have inspired hope to others. Additionally, the notion of knowledge from experience adds value to the potential contribution of the PSW and helps transform and enhance the value of lived experience.

For peer review only - http://bmjopen.bmj.com/site/about/guidelines.xhtml

Now that we are role-models in the community people inquire from us about the things which I did to enable me to stabilize while at first they were stigmatizing me. They were beating me but now it is in their families and they are having issues worse than mine. They are like, 'You see that mentally sick lady who was here? She is now stable. Let us go and inquire from her so that she can help us'…So we have become brokers in the village. [#14, Kampala, PSW]

In both the lower- and higher-income countries, PSWs reported facing stigma from health service providers. PSWs are labelled as 'mad', and in some cases, health workers question why a person with a mental illness is part of the staff team, as described by PSWs from Israel and Uganda, below.

Stigma prevails mainly among doctors and employees in medical and rehabilitation services. I blame it on the illness model as perceived by most. The model holds that illness is an inherent state, a permanent life solution. In my opinion, this is the core of the problem. Even if you have stopped medication and have been well for ten years, still the label remains. It sounds like it's for life. We should stop labelling. [#4, Be'er Sheva,PSW]

And even here at [name] hospital some of the professionals say, 'Who can work with those mad ones? But some of them, those who accepted us, are happy to work with us. They even smile at us and talk to us, but there are others who think that mental illness is contagious. [#5, Kampala, PSW]

Acceptance of the PSWs by the health service providers plays a significant role in facilitating the implementation of peer support services. PSWs' lack of acceptance from health service providers and unwillingness to work with PSWs can cause them to become "unstable" and fail to fulfil their duties, as described here by a participant from a higher-income setting.

Not being accepted made the PSW to be alone. You find that a PSW is stable in the beginning but you notice that she was destabilized in the course of being a PSW due to the pressure from outside and lack of acceptance from the team. [#009, ULM, Mental health clinician]

Although there are some health providers who stigmatize PSWs, there are others who think that PSWs are an asset to both health service providers and the recipients of peer support services. One participant from a lower-income country perceived that PSWs act as a bridge between the mental health workers and the service users. PSWs and their peers share a mutual relationship, and peers open up more to the PSWs than to mental health staff.

Actually it has bridged a gap between service users and service providers. There is some kind of mutual understanding that we have built up. We are treated like staff. [#14, Kampala, PSW]

Theme 2: Resource availability

Providing resources for PSWs to carry out their work is an important factor influencing the provision of peer support services. Several participants reported that PSWs have limited resources in terms of money for airtime (using their phone for work-based calls), transport to visit individuals in the community, and payment to cater for their daily needs when performing their

For peer review only - http://bmjopen.bmj.com/site/about/guidelines.xhtml

role, especially in lower-income settings. Two PSWs described the financial challenges they face when working in the community.

> *PSWs should receive financial support so as to be able to make home visits to service users. They should also be incentivized so as to deal with the different challenges that they face in the community. [#1, Dar es Salaam, Mental Health Clinician]*

> *According to the inflation in the country, the money can't be enough to move [travel] to a community. Sometimes you need to buy airtime to call a peer, you have to fix something or food in the community, you have to get something to drink or eat in the community. Sometimes you find that this peer you are visiting is far away from where you stay, so the money we are paid is not enough.* [#10, Kampala, PSW]

Participants from higher-income countries also reported that, whilst PSWs are an important component of mental health services, and whilst this is reflected in policy, there is a limited budget set for them, and there is a particular challenge in relation to funding arrangements for the peer support program. Differing funding arrangements across organisations and systems means that the expectation for PSWs to be employed in many departments is a challenge due to limited resources which are often stretched to cover a range of competing and differing organisational needs. In addition, uncertainties around who funds PSW programs means that cross-funding from other projects is common.

> *The current policy needs to change. The policy says peer specialists have to be everywhere…and it requires resources which we don't have. I am not sure if I can raise the issue, but we don't have budgets like the welfare…We need to get a special budget for the program.* [#6, Be'er Sheva, Mental health clinician]

> *Then there is always the question of who finances it. For example, the peer support workers on the ground floor (acute ward), are they financed by the ward budget or hospital budget or are they somehow cross-financed by other projects?* [#3, Hamburg, Mental health clinician]

The facilities available to enable PSWs to perform their roles can be inadequate. It was noted that the working environment and infrastructure in lower-resource health facilities is very poor, exposing the PSWs, mental health workers and services users to many risks. Two participants described in detail the workplace environment and the impact of this for individuals using the services and for workers.

> *The…Outpatient department can only accommodate four people while there are almost a hundred or ninety people per day, so you find that people are just standing.* [#1, Dar es Salaam, Mental health clinician]

> *We don't have enough facilities within the hospital, nurses face some challenges also. When you go to [name] ward, some peers are sleeping down [on the floor] and even divide blankets. You may find that one blanket is divided among 2 to 4 patients.* [#13, Kampala, PSW]

Theme 3: Organisational culture

For peer review only - http://bmjopen.bmj.com/site/about/guidelines.xhtml

The goals, attitudes, role assumptions and values held by the organisation about PSWs, and the relationship between the PSW and the organisation, are important for PSW implementation. Participants mainly in higher-income countries reported that working inside structured and hierarchal systems can create a feeling of indebtedness to the organisation which can impact on PSWs' autonomy in decision-making and contribute towards feelings of disempowerment.

> *When you enter a job as a consume- provider, at least in the beginning, there is part of you that feels like they are doing you a favour that they hired you. That you have to do what the organisation tells you to do in order to gain experience, etc.* [#8, Be'er Sheva, PSW]

For successful implementation of peer support work, participants explained how PSWs should work by following the rules that have been set up in the organization, and the organization should also adjust its system to accommodate the PSW. For example, organisational flexibility and understanding of the role was perceived as crucial. This included accommodations in the workplace which allow PSWs to manage their own mental health and carry out their role effectively. However, participants acknowledged that organisations' expectations were important in terms of PSWs being recognised as members of the team and as part of the organisation. Participants highlighted that organisational rules, processes, and structures were not always easy to manage or negotiate for PSWs.

> *The PSW needs to understand that he is coming here as a worker and needs to follow the expectations like any other worker. As a worker he is also entitled to some sort of accommodation system, for example it might be difficult for them to start work in the mornings because they have to take pills, so he will start at 10:00 and not 7:30. So he'll do more afternoon shifts as opposed to morning ones. [#2,* Be'er Sheva, Mental Health Clinician]

Participants highlighted that the formalisation of the PSW role in such defined systems raises the question of how much of the role should remain informal versus formal in order for PSWs to fit in. For example, the integration of PSWs into teams that already have clearly defined roles, responsibilities, and hierarchies raised uncertainties around what this might mean for the PSW role and how the introduction of the PSWs into formal systems may impact on role integrity.

> *I also think that what especially happens with peer specialists, is some sort of formalization of this thing, and how much do we really actually want to formalize it. And how much of it do we have to keep informal, which is one of the worries or dilemmas. The ideals that are really inside of this system that is so formal, and is hierarchical and clear.* [#3, Be'er Sheva, PSW]

In lower-income countries, participants reported that the support can be very limited, due to the lack of psychological and social support resources available. Participants recognised the importance of these resources for carrying out their role, but also acknowledged the difficulties and ongoing challenges of working within a system with limited resources and that follows a strongly medical model. However, in these settings, attempts were made to provide this support as much as possible despite these challenges, as one participant describes.

For peer review only - http://bmjopen.bmj.com/site/about/guidelines.xhtml

> *In summary, there is diagnosis, treatment mostly pharmacological using medical treatment. [We] do our best to try to provide psychological and social support but those are very limited most of the time.* [#30, Kampala, Manager]

Theme 4: Role definition

Nearly all participants reported that having a clear role definition and expectations was important, because without this reference point, potential role confusion and uncertainty ensue.

> *I don't think it's that easy. They often don't know what they can do themselves. That they also have ideas, what can I actually do now? And I don't think there were enough guidelines or terms of reference.* [#11, Ulm, Mental health clinician]

In addition, most participants identified that the wide variety of tasks PSWs can perform means it can be difficult to construct a role description that accurately fits with real-life peer support practice. Participants spoke in detail about how the role is performed differently depending on where the PSW works, and many participants highlighted how individual differences were also considered for specific PSW roles.

> *The task fields of peer support workers are totally different. That is always person-dependent. We tried to create a kind of job description already and that was very, very difficult. Because we didn't want to restrict the peer support workers too much. Since the tasks always depend on the personality.* [#2, Ulm, Mental health clinician]

Theme 5: Training and support

Training for PSWs and health workers is an important factor for successful implementation of PSW roles. Some participants in both lower- and higher-income countries described peer support services as something which is new to other mental health professionals, meaning that some lack knowledge of what peer support work is and what PSWs can do. Training health care workers and PSWs will help in reducing uncertainties among professionals.

> *Peer support work is something new in other countries, it needs to be introduced to other staff members in a larger scale. There is also a simple lack of knowledge, not just supervision, but knowledge of what it is all about, and this lack of knowledge also leads to uncertainty among professionals. What, how do we deal with it now, what do we trust them to do, what do we take away from them because it is not the right thing to do? [#11, ULM, Mental health clinician]*

The availability of initial training enables PSWs to know what is expected from them and also understand their needs. Initial training was identified as key for PSWs being prepared for working in the role from both lower and higher income countries. Many participants highlighted that training which provides an understanding of the different types of PSW activities and the work-based challenges PSWs may face was important. Some examples of the training content identified as key for initial training included knowledge of the varying attitudes towards mental health, working with individuals in distress, their families, PSWs, and other mental health workers.

> *The first thing that they are supposed to receive is training. If they receive training they will know their job description and the techniques of going to the families because there are families which don't want people to know that they have a mental patient.* [#3, Dar es Salaam, Mental health clinician]

For peer review only - http://bmjopen.bmj.com/site/about/guidelines.xhtml

I can say that the PSW program that I was part of had PSWs who first of all received training especially to understand their needs, making sure they are dealing with mental illness of others and also how they work with PSWs. [#30, Kampala, Manager]

Most participants also identified the need for further and ongoing training opportunities to be provided. Ongoing training that was highlighted as important for PSWs included understanding boundaries, knowledge about the code of conduct, and levels of disclosure. Continual training was viewed as an expectation that should be in place and carried out, so PSWs can continue to carry out their role effectively along with developing knowledge and learning new skills.

Peer support workers need more training, continuous training. Even if the training is a one-off. So this should be happening. It shouldn't be a big deal. [#30, Kampala, Manager]

Participants in both lower- and higher-income countries described how PSWs need support to maintain their wellbeing and carry out their roles. Guidance, supervision, and support from other health care workers is very important for PSWs in carrying out their work, from a practical perspective. It also enables them to feel part of the wider team, rather than feeling as though they are working in isolation. Participants from both high- and lower-income countries described the importance of ongoing support.

Coming from a person who is currently dealing with very active symptoms with varying levels of force, a person needs…regulatory capacity, the ability to manage workloads, the ability to receive help, to be helped and to defend oneself. [#1, Be'er Sheva, Mental health clinician]

They shouldn't always work in isolation; they should be supervised. [#24, Kampala, Mental health clinician]

Peer support workers can't be independent, they need professional community nurses to guide them so they can go out in the field, they can be together. [#30, Kampala, Manager]

Theme 6: Peer support network

Participants in both lower- and higher-income countries explained that having access to a peer network enables PSWs to address their potential challenges, makes them feel connected through sharing their experiences and also enables them to feel stronger together. Further, through these networks PSWs can get to know each other and identify if another PSW is facing a problem, looking after the well-being of the network's members.

I can see how a group of peers impact each other non-stop and advances processes almost as if it is a race, but not in a bad way. That is to say, not from a place where you feel that they are forcing you to run, but from a place where a lot of people who are together all the time, are shattering stigmas about one another, I think that a group is stronger. [#3, Be'er Sheva, PSW]

For peer review only - http://bmjopen.bmj.com/site/about/guidelines.xhtml

We peers have what they call buddies. A buddy is a person who knows more about you whereby in case you show signs of relapse that buddy will say, '(Name) is getting a relapse, (name) do this and this'. He will help you and bring you medical personnel and overcome the situation. [#11, Kampala, PSW]

Discussion

Our study identified six influences on PSW implementation. At the societal level, community stigma and lay beliefs about mental health conditions were influential. At the organisational level, the inter-linked themes of resource allocation and organisational culture were identified, as well as staff attitudes and the challenge of ensuring role clarity. At the PSW level, both adequate training /support and a strong peer support network were facilitators of implementation.

Two aspects of our findings are noteworthy in relation to other studies. First, the conceptual framework was developed on the basis of research almost exclusively from high-income countries, and identified PSW and organisational influences. The implementation influences identified by participants had a stronger emphasis on societal aspects, including attitudes and role assumptions. Our findings are consistent with the previously-discussed systematic review,[21] published since the conceptual framework was developed. This validates the importance of considering organisational and societal aspects when implementing PSW in different resource settings.

This involves developing community awareness regarding the value of peer support, to gain the support of family and community members.[26] Second, the PSW-level influences indicate the need to modify how PSW is provided in different settings. A systematic review of 39 studies (only one from a lower-income setting), identified seven types of modification to the PSW role,[27] including recruitment processes, role expectations, training and support. Recent research is expanding to also consider staff attitudes,[28] organisational integration of PSWs,[29,30] _ENREF_32 organisational climate[31] and context.[32,33]

The primary implication is that more attention needs to be paid to societal attitudes and organisational culture in developing and implementing PSW programmes. Discrimination and stigma relating to mental health are global challenges,[4] but our findings suggest that there is a relationship between community attitudes and the ability to involve people with lived experience in the mental health workforce as PSWs. In terms of organisational culture, the findings reinforce existing evidence[34] that organisational culture impacts on recovery support, so organisational transformation may be needed.[35] Approaches to supporting culture change within mental health services include the introduction of pro-recovery interventions,[36,37] development of adjunctive services such as Recovery Colleges,[38] working with teams[39] and introducing co-production[40] and growth-oriented approaches.[41]

A better understanding of the relationship between the identified influences is needed. In UPSIDES, the theory of change technique is being used to map out different steps in the implementation of the PSW intervention, and to articulate the connections between these steps. The impact of societal and organisational influences on PSW effectiveness will be further explored in the multi-national UPSIDES randomised controlled trial (ISRCTN26008944) which is currently underway.[42]

The strengths of the study include the sample size, the use of multiple informants, using local language topic guides to avoid excluding non-English speaking participants, and the multi-

For peer review only - http://bmjopen.bmj.com/site/about/guidelines.xhtml

national sample. Credibility of the findings was enhanced by independent coding and the use of multiple analysts.

Several limitations can be identified. One significant shortcoming is that sociodemographic characteristics of participants were not collected in a standardised way across all sites, so are not reported here— limiting the transferability of findings. Whilst the sample is large for a qualitative study, the findings are complex and nuanced, so our analysis focused on semantic rather than latent coding[25]. Future analysis might explore the relationship between the identified implementation influences, such as how community attitudes may distally impact on resource allocation. Whilst the use of analysts with different professional backgrounds reduced researcher influence on findings, the credibility of the findings could be enhanced by member checking, and including people with lived experience as co-analysts.[43] Finally, the relatively small number of policy-maker participants may account for the limited mention of national and regional policy as an influence.

Conclusions

This is the first multi-country study to explore societal attitudes and organisational culture influences on the implementation of peer support. Addressing community-level stigma and discrimination and developing a recovery orientation in mental health systems can contribute to effective implementation of peer support work.

For peer review only

For peer review only - http://bmjopen.bmj.com/site/about/guidelines.xhtml

Acknowledgements
The Using Peer Support In Developing Empowering Mental Health Services (UPSIDES) Study is a multicentre collaboration between the Department for Psychiatry and Psychotherapy II at Ulm University, Germany (Bernd Puschner, coordinator); the Institute of Mental Health at University of Nottingham, UK (Mike Slade); the Department of Psychiatry at University Hospital Hamburg-Eppendorf, Germany (Candelaria Mahlke); Butabika National Referral Hospital, Uganda (David Basangwa); the Centre for Global Mental Health at London School of Hygiene and Tropical Medicine, UK (Grace Ryan); the Department of Health Systems, Impact Evaluation and Policy, Ifakara Health Institute, Dar es Salaam, Tanzania (Donat Shamba); the Department of Social Work at Ben Gurion University of the Negev, Be'er Sheva, Israel (Galia Moran); and the Centre for Mental Health Law and Policy, Pune, India (Jasmine Kalha).

Contributors
MR, AC contributed to data acquisition, analysis and interpretation and drafting the manuscript. AG, RH, RN, AK, JK, CM, GM, RM, AMS, GR, DS and MS contributed to the design of the work, data acquisition, analysis and interpretation, and critically revised the work for important intellectual content. MR, AC, AG, RH, RN, AK, JK, CM, GM, RM, AMS, GR, DS and MS gave final approval of the version to be published, and are accountable for all aspects of the work.

Funding
This work was supported by the European Union Horizon 2020 Research and Innovation Programme under Grant Agreement No 779263. MS acknowledges the support of the Center for Mental Health and Substance Abuse, University of South-Eastern Norway and the NIHR Nottingham Biomedical Research Centre. This publication reflects only the authors' views. The Commission is not responsible for any use that may be made of the information it contains. The funding body had no role in the design of the study and in writing the manuscript.

Competing interests
None declared.

Data availability statement
Data are available upon reasonable request.

For peer review only

For peer review only - http://bmjopen.bmj.com/site/about/guidelines.xhtml

References

1. Samele C, Frew, S., Urquia, N. European profile of prevention and promotion of mental health (EuroPoPP-MH): EU Health Programme [(EAHC/2010Health/04)]; 2013.
2. Pathare S, Brazinova A, Levav I. Care gap: a comprehensive measure to quantify unmet needs in mental health. *Epidemiology and Psychiatric Sciences* 2018; **27**: 463-7.
3. Collins PY, Patel V, Joestl SS, March D, Insel TR, Daar AS. Grand challenges in global mental health. *Nature* 2011; **475**(7354): 27-30.
4. Patel V, Saxena S, Lund C, et al. The Lancet Commission on global mental health and sustainable development. *Lancet* 2018; **392**: 1553-98.
5. Misra S, Stevenson A, Haroz E, de Menil V, Koenen K. 'Global mental health': systematic review of the term and its implicit priorities. *Br J Psychiatry* 2019; **5**: e47.
6. Davidson L, Bellamy C, Guy K, Miller R. Peer support among persons with severe mental illnesses: a review of evidence and experience. *World Psychiatry* 2012; **11**: 123-8.
7. Mead S. Intentional Peer Support: an alternative approach. Plainfield, NH: Shery Mead Consulting; 2005.
8. Pitt V, Lowe D, Hill S, et al. Consumer-providers of care for adult clients of statutory mental health services. *Cochrane Database of Systematic Reviews* 2013; **3**: CD004807.
9. Lloyd-Evans B, Mayo-Wilson E, Harrison B, et al. A systematic review and meta-analysis of randomised controlled trials of peer support for people with severe mental illness. *BMC Psychiatry* 2014; **14**: 39.
10. Shalaby R, Agyapong V. Peer Support in Mental Health: Literature Review. *JMIR Mental Health* 2020; **7**: e15572.
11. Chinman M, George, P., Dougherty, R., Daniels, A., Ghose, S., Swift, A., Delphin-Rittmon, M. Peer Support Services for Individuals With Serious Mental Illnesses: Assessing the Evidence. *Psychiatr Serv* 2014; **65**: 429-41.
12. White S, Foster R, Marks J, et al. The effectiveness of one-to-one peer support in mental health services: a systematic review and meta-analysis. *BMC Psychiatry* 2020; **20**: 534.
13. Repper J. ImROC Briefing Paper 5. Peer Support Workers: Theory and Practice. London: Centre for Mental Health; 2013.
14. Mead S, Hilton, D., Curtis, L. Peer support: A theoretical perspective. *Psychiatric Rehabilitation Journal* 2001; **25**: 134–41.
15. Fan Y, Ma N, Ma L, Xu W, Lamberti JS, Caine E. A community-based peer support service for persons with severe mental illness in China. *BMC Psychiatry* 2018; **18**: 170.
16. Pathare S, Kalha J, Krishnamoorthy S. Peer support for mental illness in India: An underutilised resource. *Epidemiology and Psychiatric Sciences* 2018; **27**: 415-9.
17. Moran GS. The mental health consumer movement and peer providers in Israel. *Epidemiology and Psychiatric Sciences* 2018; **27**: 420-6.
18. Lee YY, Ang S, Chua HC, Subramaniam M. Peer Support in Mental Health: A Growing Movement in Singapore. *Annals of Academy of Medicine Singapore* 2019; **48**: 95-7.
19. Ryan G, Kamuhiirwa M, Mugisha J, et al. Peer support for frequent users of inpatient mental health care in Uganda: protocol of a quasi-experimental study. *BMC Psychiatry* 2019; **19**: 374.
20. Piat M, Sofouli E, Sabetti J, et al. Protocol for a mixed studies systematic review on the implementation of the recovery approach in adult mental health services. *BMJ Open* 2017; **7**(8).
21. Ibrahim N, Thompson D, Nixdorf R, et al. A systematic review of influences on implementation of peer support work for adults with mental health problems. *Soc Psychiatry Psychiatr Epidemiol* 2020; **55**: 285-93.

For peer review only - http://bmjopen.bmj.com/site/about/guidelines.xhtml

22. Puschner B, Repper J, Mahlke C, et al. Using Peer Support in Developing Empowering Mental Health Services (UPSIDES): Background, rationale and methodology. *Annals of Global Health* 2019; **85**(53): 1-10.

23. Jabareen Y. Building a Conceptual Framework: Philosophy, Definitions, and Procedure. *International Journal of Qualitative Methods* 2009; **8**: 49-62.

24. Repper J, Perkins R. ImROC Briefing Paper 6. The Team Recovery Implementation Plan: a framework for creating recovery-focused services. London: Centre for Mental Health; 2013.

25. Braun V, Clarke V, Hayfield N, Terry G. Thematic analysis. In: Laiamputtong L, ed. Handbook of Research Methods in Social Sciences. Singapore: Springer; 2019: 843-60.

26. Pearson H, Adams B, Eaton J, Illingworth M. Community Mental Health Good Practice Guide: Peer Support: CBM Global Disability Inclusion; 2021.

27. Charles A, Thompson D, Nixdorf R, et al. A typology of modifications to peer support work for adults with mental health problems: systematic review. *Br J Psychiatry* 2020; **216**: 301-7.

28. Moore T, Zeeman L. More 'milk' than 'psychology or tablets': Mental health professionals' perspectives on the value of peer support workers. *Health Expectations* DOI: 10.1111/hex.13151.

29. Charles A, Nixdorf R, Ibrahim N, et al. Initial training for mental health peer support workers: international Delphi Consultation. *JMIR Mental Health* 2021; **5**: e25528.

30. Otte I, Werning A, Nossek A, Vollmann J, Juckel G, Gather J. Challenges faced by peer support workers during the integration into hospital-based mental health-care teams: Results from a qualitative interview study. *Int J Soc Psychiatry* 2020; **66**: 263-9.

31. Jones N, Teague G, Wolf J, Rosen C. Organizational Climate and Support Among Peer Specialists Working in Peer-Run, Hybrid and Conventional Mental Health Settings. *Administration and Policy in Mental Health and Mental Health Services Research* 2020; **47**: 150-67.

32. Adams W. Unintended consequences of institutionalizing peer support work in mental healthcare. *Soc Sci Med* 2020; **262**: https://doi.org/10.1016/j.socscimed.2020.113249.

33. Urichuk L, Hrabok M, Hay K, et al. Enhancing peer support experience for patients discharged from acute psychiatric care: protocol for a randomised controlled pilot trial. *BMJ Open* 2018; **8**: e022433.

34. Clossey L, Rheinheimer D. Exploring the effect of organizational culture on consumer perceptions of agency support for mental health recovery. *Community Ment Health J* 2014; **50**(4): 427-34.

35. Le Boutillier C, Chevalier A, Lawrence V, et al. Staff understanding of recovery-orientated mental health practice: a systematic review and narrative synthesis. *Implementation Science* 2015; **10**: 87.

36. Meadows G, Brophy L, Shawyer F, et al. REFOCUS-PULSAR recovery-oriented practice training in specialist mental health care: a stepped-wedge cluster randomised controlled trial. *Lancet Psychiatry* 2019; **6**: 103-14.

37. Slade M, Bird V, Clarke E, et al. Supporting recovery in patients with psychosis through care by community-based adult mental health teams (REFOCUS): a multisite, cluster, randomised, controlled trial. *Lancet Psychiatry* 2015; **2**: 503-14.

38. Crowther A, Taylor A, Toney R, et al. The impact of Recovery Colleges on mental health staff, services and society. *Epidemiology and Psychiatric Sciences* 2019; **28**: 481-8.

39. Vacher G. Utilising Team Recovery Implementation Plan (TRIP): embedding recovery-focused practice in rehabilitation services. *Mental Health and Social Inclusion* 2017; **21**: 240-7.

40. Lewis A, King T, Herbert L, Repper J. Co-Production – Sharing Our Experiences, Reflecting On Our Learning. Nottingham: ImROC; 2017.

41. Slade M, Rennick-Egglestone S, Blackie L, et al. Post-traumatic growth in mental health recovery: qualitative study of narratives. *BMJ Open* 2019; **9**: e029342.

For peer review only - http://bmjopen.bmj.com/site/about/guidelines.xhtml

42. Moran G, Kalha J, Mueller-Stierlin A, et al. Peer support for people with severe mental illness versus usual care: study protocol for a pragmatic multicentre randomised controlled trial (UPSIDES-RCT). *Trials* 2020; **21**: 371.
43. Jennings H, Slade M, Bates P, Munday E, Toney R. Best practice framework for Patient and Public Involvement (PPI) in collaborative data analysis of qualitative mental health research: methodology development and refinement. *BMC Psychiatry* 2018; **18**: 213.

For peer review only

For peer review only - http://bmjopen.bmj.com/site/about/guidelines.xhtml

Table 1: Conceptual framework for PSW principles (n=8) and societal / organisational implementation influences (n=5)

Principle	Definition
1. Mutual	Peer support workers have similar experiences to peer support users
2. Reciprocal	Peer support workers and peer support users both give and receive in the relationship
3. Non-directive	Peer support workers develop solutions together with the peer support user, instead of dictating solutions
4. Recovery focused	Peer support workers support the peer support user on his/her path towards overcoming the problems that they experience
5. Strengths-based	Peer support workers show a positive attitude and identify and build on the strengths and recovery progress of peer support users
6. Inclusive	Peer support workers do not exclude people on the basis of the nature of their problems or beliefs about their level of ability, and help peer support users to find their place in society
7. Progressive	Peer support workers and users advance together towards recovery, this is not a befriending relationship that aims to maintain current progress
8. Safe	Peer support workers and users develop a common basis of trust and safety, which is central to the planning of the service and training of peer workers
**Implementation influence**	**Description of societal / organisational influence**
1. Group versus individual	Peer support can be offered in single sessions and in a group setting
2. Extent to which both parties choose to enter the relationship	Peer support pairs and groups can be formed by the organisation, but also by the peers themselves
3. Extent to which rules govern the relationship	There can be implicit and explicit rules underpinning how the peer support work is conducted
4. Extent to which the parties involved are in the same place in their recovery journey	Depending on the state of recovery, peer support users can become peer support workers and vice versa
5. Extent to which the peer support workers focus on peer support users	Peer support workers can support recovery for peer support users and/or promote a recovery orientation for the staff they work with, the institution they work in, and the society they live in

For peer review only - http://bmjopen.bmj.com/site/about/guidelines.xhtml

Table 2: Focus group participants (n=86)

Site	Focus groups	n	Participant				Gender	
			Potential or actual PSW	Mental health worker	Mental health manager	Policy-maker	Male	Female
Ulm	2	12	1	10	0	1	4	8
Hamburg	2	12	7	5	0	0	4	8
Kampala	4	32	16	14	1	1	10	22
Dar es Salaam	2	16	0	12	4	0	7	9
Be'er Sheva	2	14	2	8	4	0	5	9
**Total**	**12**	**86**	**26**	**49**	**9**	**2**	**30**	**56**

For peer review only

For peer review only

Instructions:

This guide is to be used to facilitate focus group discussion with (1.1) health workers who are using or plan to recruit peer support workers. (Psychiatric nurses, clinicians working in mental health ward / departments) and (1.2) local stakeholders with relevant expertise relating to implementation of peer work (including clinicians and managers who currently, previously or in the future may employ peer support workers, and people who currently, previously or in the future may work as peer support workers).

Interviewer ID ___ ___ ___ ___

Interview date (DD/MM/YYYY): __ __ / __ __ / __ __ __

Location of the interview ____________________________________

Country: ____________________________________

Introduction:

Ensure that the participants are comfortable.

Hi and welcome to this session. My name is ___ and my colleague is___. Thank you for taking your time to talk to us about your experiences with people with severe mental illness and peer support workers. We will discuss topics related to the key characteristics of peer support workers and the challenges they may face. The discussion will take around 60minutes. The interview will be recorded using an audio recorder as we will not have to write down all your answers and we will not miss any of your useful comments. Informed consent will be obtained from all the participants and confidentiality will be assured to all the participants. Before we begin, I would like to know if you have any questions.

Explain you are starting the audio recorder.

Time interview started:

Theme 1: Socio-demographic information of the respondents

Collect data on name, age, education, position and number of years working in this position and give a code to each participant.

Explain again to the participant that:

- I want to learn about your experience, thoughts and perspective on this topic of using peer support workers for improving mental health conditions in your country.
- There is no 'right' or 'wrong' answer.

Say: We are planning to recruit and train peer support workers. Peer providers in mental health are individuals with severe mental illness who are further along in their recovery, who support others with similar conditions by role modelling that recovery is possible, sharing knowledge from experience and using reciprocal empathic relationships. We are thinking of asking these peer support workers to identify and visit individuals with severe mental illness. We would like to get your opinion on how best to implement PSWs intervention in this region. We would like to know key characteristics for PSWs and challenges that could hinder them performing their

For peer review only - http://bmjopen.bmj.com/site/about/guidelines.xhtml

tasks. Please be frank as we want the information to be the right ones for this community.

Theme 2: Social cultural factors and acceptability of PSWs

1. Based on your experiences, how does the community care for people with SMIs? Where do they seek care? Are there other places apart from the hospital? Describe all the places people seek care for SMI.

2. What are your main roles in supporting people with SMIs? What are the main challenges that you are facing in doing your job? What are the solutions to those challenges?

   - Probe to know if mental health programmes are given a priority?
   - Is there a national policy or guideline for mental health conditions? Are these accessible?
   - What has been the main challenge your department has been facing in managing/meeting the needs of people with Severe Mental Illness? Probe for various challenges the department has been facing in regard to SMI programmes? For each challenge mentioned, ask how the department could handle it i.e. any potential solution and/or suggestion on how to address it?

3. With your experience working in this area, could you please tell us what do you know about PSWs and how do they perform their duties?

   - Probe for, how they are recruited, who recruits them, what are they exactly doing, etc?
   - Please, describe any systems for linking people with peers who can serve as role models in this facility or community? (e.g. through contact with local user-run groups).

4. What key qualities would you want a peer support worker to have or NOT to have? We want to find out what sort of person the respondents would trust and value, perform the work well and responsibly.

5. Could you please tell us what are the specific things, you would want a peer support worker to do?

   Probe for specific terminologogies, main activities, duration of activities. care planning

Theme 3: Institution / facility readiness to incorporate PSW

6. In your opinion – for using peer support workers, how could it be implemented / improved at this facility?

For peer review only - http://bmjopen.bmj.com/site/about/guidelines.xhtml

- Probe: training, logistics, management, supervision, monitoring, capacity development, resources etc.

7. What are the facilitators and barriers in providing peer support to people with SMI in your facility or in your region?

    - Probe for resources, skilled personnel, infrastructure, systems support?

8. Is there anything more you would like to add about your experiences with, or views on, using peer support workers?

Time interview end:

Thank the participant for his / her time. Remind them that the information will be kept confidential.

Interviewer comments on how the interview went:

For peer review only

Standards for Reporting Qualitative Research Checklist

Item	Topic	Where addressed
**Title and abstract**		
S1	**Title**<br>Concise description of the nature and topic of the study Identifying the study as qualitative or indicating the approach (e.g., ethnography, grounded theory) or data collection methods (e.g., interview, focus group) is recommended	Title
S2	**Abstract**<br>Summary of key elements of the study using the abstract format of the intended publication; typically includes background, purpose, methods, results, and conclusions	Abstract
**Introduction**		
S3	**Problem formulation** Description and significance of the problem/phenomenon studied; review of relevant theory and empirical work; problem statement	Introduction
S4	Purpose or research question Purpose of the study and specific objectives or questions	Introduction final paragraph
**Methods**		
S5	**Qualitative approach and research paradigm**<br>Qualitative approach (e.g., ethnography, grounded theory, case study, phenomenology, narrative research) and guiding theory if appropriate; identifying the research paradigm (e.g., postpositivist, constructivist/interpretivist) is also recommended; rationale	Methods Design<br>Method Procedures paragraph 2<br>Methods Analysis
S6	**Researcher characteristics and reflexivity**<br>Researchers' characteristics that may influence the research, including personal attributes, qualifications/experience, relationship with participants, assumptions, and/or presuppositions; potential or actual interaction between researchers' characteristics and the research questions, approach, methods, results, and/or transferability	Methods Analysis<br>Discussion paragraph 2
S7	**Context**	Methods Setting

For peer review only

	Setting/site and salient contextual factors; rationale	
S8	**Sampling strategy**<br>How and why research participants, documents, or events were selected; criteria for deciding when no further sampling was necessary (e.g., sampling saturation); rationale	Methods Participants<br>Methods Procedures final paragraph<br>Methods Sampling
S9	**Ethical issues pertaining to human subjects** Documentation of approval by an appropriate ethics review board and participant consent, or explanation for lack thereof; other confidentiality and data security issues	Methods Ethics approval
S10	**Data collection methods** Types of data collected; details of data collection procedures including (as appropriate) start and stop dates of data collection and analysis, iterative process, triangulation of sources/methods, and modification of procedures in response to evolving study findings; rationale. The rationale should briefly discuss the justification for choosing that theory, approach, method, or technique rather than other options available, the assumptions and limitations implicit in those choices, and how those choices influence study conclusions and transferability. As appropriate, the rationale for several items might be discussed together.	Methods Procedures<br>Methods Analysis
S11	**Data collection instruments and technologies**<br>Description of instruments (e.g., interview guides, questionnaires) and devices (e.g., audio recorders) used for data collection; if/how the instrument(s) changed over the course of the study	Methods Procedures paragraphs 1 and 2
S12	**Units of study**<br>Number and relevant characteristics of participants, documents, or events included in the study; level of participation (could be reported in results)	Results Table 1
S13	**Data processing**<br>Methods for processing data prior to and during analysis, including transcription, data entry, data management and security, verification of data integrity, data coding, and anonymization/deidentification of excerpts	Methods Procedures final paragraph
S14	**Data analysis**<br>Process by which inferences, themes, etc., were identified and developed, including the researchers involved in data analysis; usually references a specific paradigm or approach; rationale	Methods Analysis

For peer review only

S15	**Techniques to enhance trustworthiness** Techniques to enhance trustworthiness and credibility of data analysis (e.g., member checking, audit trail, triangulation); rationale	Methods Analysis Discussion strengths and limitations paragraph
**Results/findings**		
S16	**Synthesis and interpretation** Main findings (e.g., interpretations, inferences, and themes); might include development of a theory or model, or integration with prior research or theory	Results
S17	**Links to empirical data** Evidence (e.g., quotes, field notes, text excerpts, photographs) to substantiate analytic findings	Results
**Discussion**		
S18	**Integration with prior work, implications, transferability, and contribution(s) to the field** Short summary of main findings; explanation of how findings and conclusions connect to, support, elaborate on, or challenge conclusions of earlier scholarship; discussion of scope of application/generalizability; identification of unique contribution(s) to scholarship in a discipline or field	Discussion
S19	**Limitations** Trustworthiness and limitations of findings	Discussion paragraph 2
**Other**		
S20	**Conflicts of interest** Potential sources of influence or perceived influence on study conduct and conclusions; how these were managed	Competing Interests statement
S21	**Funding** Sources of funding and other support; role of funders in data collection, interpretation, and reporting	Funding statement

For peer review only
